# Novel amide-functionalized chloramphenicol base bifunctional organocatalysts for enantioselective alcoholysis of *meso*-cyclic anhydrides

**DOI:** 10.3762/bjoc.14.19

**Published:** 2018-01-31

**Authors:** Lingjun Xu, Shuwen Han, Linjie Yan, Haifeng Wang, Haihui Peng, Fener Chen

**Affiliations:** 1 Department of Chemistry, Fudan University, Shanghai 200433, PR China

**Keywords:** alcoholysis desymmetrization, bifunctional organocatalysis, chloramphenicol base

## Abstract

A family of novel chloramphenicol base-amide organocatalysts possessing a NH functionality at C-1 position as monodentate hydrogen bond donor were developed and evaluated for enantioselective organocatalytic alcoholysis of *meso*-cyclic anhydrides. These structural diversified organocatalysts were found to induce high enantioselectivity in alcoholysis of anhydrides and was successfully applied to the asymmetric synthesis of (*S*)-GABOB.

## Introduction

**Figure 1:**
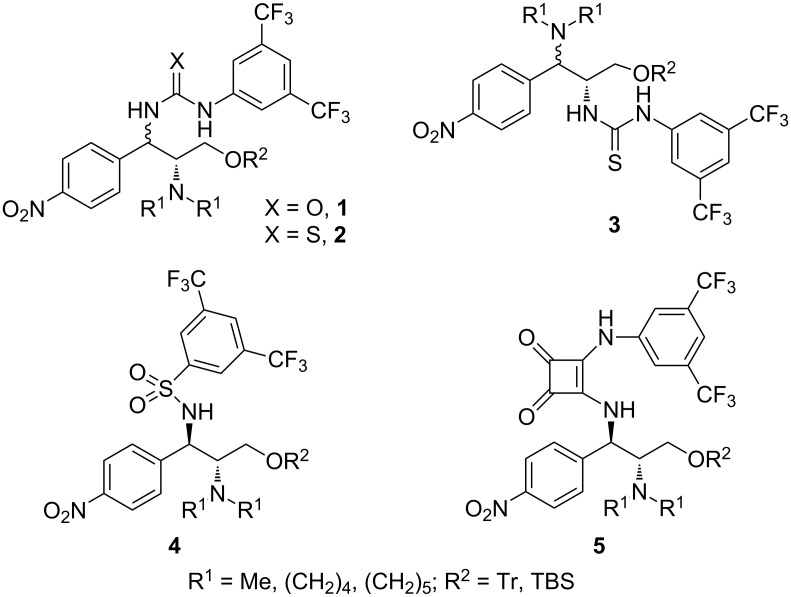
Chloramphenicol-base-derived bifunctional organocatalysts.

Over the past decade, remarkable advances in the utilization of natural products as chiral structural motifs for the design of bifunctional organocatalysts have been achieved. A high stereocontrol in alcoholytic catalytic asymmetric desymmetrizations of *meso*-cyclic anhydrides is of special interest [1–7]. Major families originating from natural products include cinchona alkaloids [8–17] and proteinogenic α-amino acids such as proline [18–20] and valine [21] etc. [22–29], while fully synthetic catalysts are rare, only involving Brønsted acid/base catalysts [30], cyclohexanediamine catalysts [31–32], and diphenylethylenediamine catalysts [33–34]. Significant progress in our laboratory has been made in the development of chiral bifunctional urea **1** [35], thiourea **2** and **3** [36–37], sulfonamide **4** [38–40] and squaramide **5** [38–40] catalysts derived from chloramphenicol base (Figure 1), which showed excellent reactivity and enantioselectivity for this asymmetric transformation. A typical example of the great utility of chloramphenicol base scaffold in industrial production of enantiopure compounds is the synthesis of a precursor of biotin (vitamin H) by the chloramphenicol base-promoted asymmetric methanolysis of *meso*-cyclic anhydrides [41–43].

**Figure 2:**
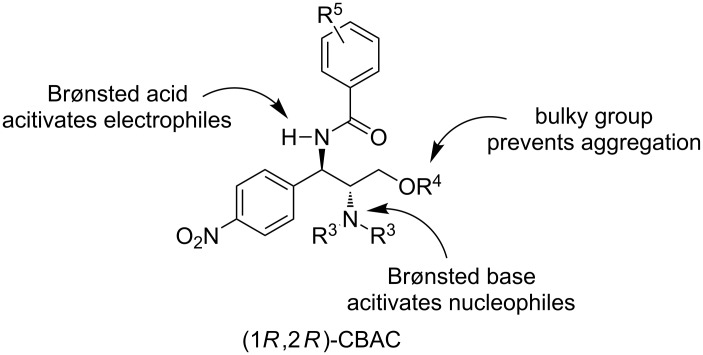
Design of new chloramphenicol base amide organocatalysts.

Despite extensive studies on chloramphenicol-base-derived bifunctional derivatives catalyzed asymmetric alcoholysis of anhydride, the exploration of new effective and easily accessible fully synthetic organocatalysts through further modification of this privilege motif are always needed. Our present design is inspired by cinchona-derived sulfonamides, first developed by Song and co-workers [44–46]. Because of its easy availability and unique stereochemical aspect, we were therefore intrigued by the possibility of modifying the chloramphenicol base structural backbone by virtue of substituting the hydroxy group at C-1 position by a simple amide moiety as monodentate hydrogen bond donor to activate the electrophile (anhydride), whilst retaining the tertiary amine functionality to activate the nucleophile (alcohol, Figure 2). As part of our ongoing research program on chloramphenicol base organocatalysis, herein, we report a new class of chloramphenicol-derived bifunctional amide organocatalysts and their application for enantioselective alcoholysis of *meso*-cyclic anhydrides.

In the precedented urea- or thiourea-based organocatalytic methanolysis of anhydrides, one big problem is the homo-aggregation of the catalysts via hydrogen bonding, which decreased the reactivity and enantioselectivity and required diluted concentrations with low temperatures [47]. With the new chloramphenicol base amide organocatalysts we envisioned that the presence of a bulky oxygen group could avoid the intermolecular aggregation of the catalyst, while keeping the desired bifunctional Brønsted acid/base reactivity for enantioselective desymmetrization of anhydrides [48–49].

## Results and Discussion

**Scheme 1:**
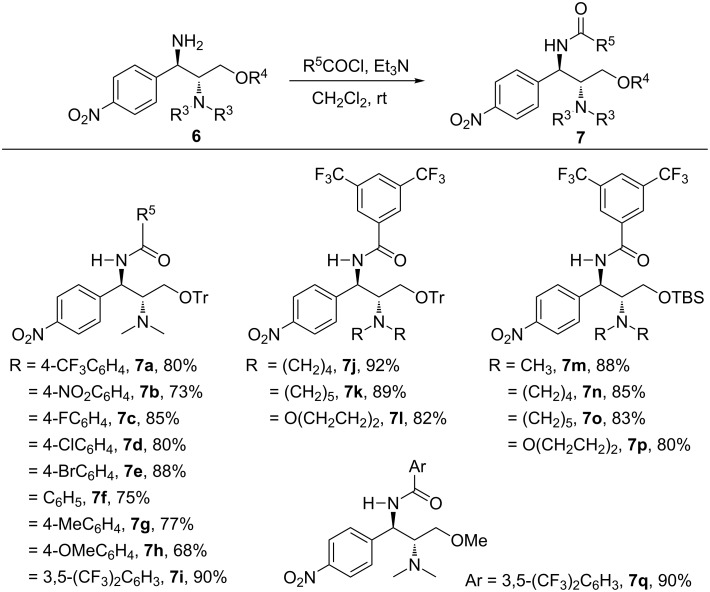
Synthesis of bifunctional amide catalysts **7a–q**.

A series of new chloramphenicol based-amide bifunctional catalysts **7a**–**q** (Scheme 1) were synthesized from the appropriate optically pure (1*R*,2*R*)-diamine **6**, prepared via several steps from chloramphenicol base [50]. In general, the synthesis is straightforward and well-compatible to electronically-diversified amides. Particularly, electron-deficient amides performed better. With 3,5-(CF_3_)_2_-disubstituted amide, the corresponding pruduct **7i** was achieved with 90% yield. Different protecting groups for the nitrogen at the C-2 position and oxygen at C-3 are also investigated to yield the desired chiral catalysts using a simple procedure.

With various bifunctional catalysts in hand, assessment of their catalytic behavior in enantioselective alcoholysis of *meso*-cyclic anhydride **8a** was carried out in various conditions with 10 equivalents of methanol. Resultingly, this novel Brønsted acid/base catalysts **7a**–**q** proved to be sufficiently active and gave the monoester product with high yield and superior enantioselectivity, suggesting the unique reactivity of this amide-based chloramphenicol scaffold.

Electronic effects were first investigated. Generally, electron deficient catalysts gave better reactivity and enantioselectivity than electron-rich variants (Table 1, entries 1–9). Among them, **7i** performed best with 95% yield and 92% ee in 17 h (Table 1, entry 9). The surprising reactivity with this electron-poor **7i** suggested that the p*K*a is crucial for this catalysis. Further modifications of the chloramphenicol skeleton with various protecting groups on the nitrogen at C-2 position and oxygen at C-3 did not improve the reaction but with lowered enantioselectivity (Table 1, entries 10–16). Notably, with methyl-protected catalyst **7q**, this reaction proceeded with longer reaction time but with decreased enantioselectivity, suggesting the effects of a bulky ether group (Table 1, entry 17). Solvent effects were then evaluated and aprotic solvent performed better than protic solvents (Table 1, entries 18 and 19), which is in accordance with literature precedence [45]. Furthermore, changing concentration or low down the temperature of the reaction didn’t alter the yield or enantioselectivity, suggesting the concentration/temperature independence of this catalytic system (Table 1, entries 20–23). These results implicated that there is no significant aggregation of catalysts in this system.

**Table 1:** Conditions screening for asymmetric methanolysis of *meso*-cyclic anhydrides.

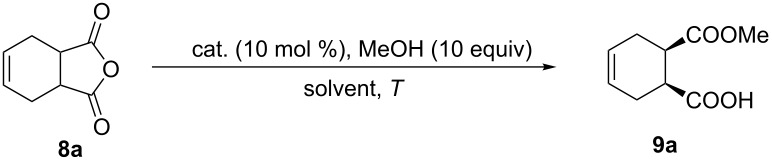							

Entry	Cat.	Solvent	Conc. (M)	*T* (°C)	Time (h)	Yield^a^ (%)	ee^b,c^ (%)

1	**7a**	MTBE	0.05	20	34	91	84
2	**7b**	MTBE	0.05	20	36	94	80
3	**7c**	MTBE	0.05	20	40	95	77
4	**7d**	MTBE	0.05	20	46	94	80
5	**7e**	MTBE	0.05	20	48	99	83
6	**7f**	MTBE	0.05	20	58	100	78
7	**7g**	MTBE	0.05	20	96	99	72
8	**7h**	MTBE	0.05	20	48	100	76
9	**7i**	MTBE	0.05	20	17	95	92
10	**7j**	MTBE	0.05	20	20	85	80
11	**7k**	MTBE	0.05	20	58	99	71
12	**7l**	MTBE	0.05	20	216	100	58
13	**7m**	MTBE	0.05	20	28	95	84
14	**7n**	MTBE	0.05	20	24	91	76
15	**7o**	MTBE	0.05	20	192	97	82
16	**7p**	MTBE	0.05	20	240	98	68
17	**7q**	MTBE	0.05	20	58	93	54
18	**7i**	Toluene	0.05	20	14	96	79
19	**7i**	MeOH	0.05	20	18	91	10
20	**7i**	MTBE	0.0125	20	86	99	94
21	**7i**	MTBE	0.1	20	12	95	87
22	**7i**	MTBE	0.05	0	120	99	95
23	**7i**	MTBE	0.05	−20	384	92	96

^a^Isolated yield. ^b^Determined by HPLC after conversion to amide derivatives with (*S*)-1-phenylethylamine. ^c^The absolute configuration was determined by comparing with literature report.

This bifunctional Brønsted acid/base catalyst **7i** proved to be highly active toward asymmetric methanolysis of various *meso*-cyclic anhydrides. As shown in Table 2, various cyclic anhydrides were conveniently converted into the corresponding monoester products in high yields and enantioselectivities. Notably, bicyclic anhydrides (Table 2, entries 1 and 2) and tricyclic anhydrides (Table 2, entries 3 and 4) were more reactive than mono-cyclic anhydrides (Table 2, entries 5–12). In the case of mono-substituted cyclic substrates, including alkyl- (Table 2, entries 5–7), ether- (entry 8), aryl-substitution (entries 9–11), moderate enantioselectivities were detected. Moreover, the chiral mono-substituted products are versatile key building blocks for the synthesis of a variety of the industrially important pharmaceuticals, such as γ-aminobutyric acid (GABA) and γ-amino-β-hydroxybutyric acid (GABOB) analogues (baclofen HCl and pregabalin) [51–54], HMG-CoA reductase inhibitors (“statins”) [55–57], etc.

**Table 2:** Asymmetric methanolysis of *meso*-cyclic anhydrides.^a,b^.

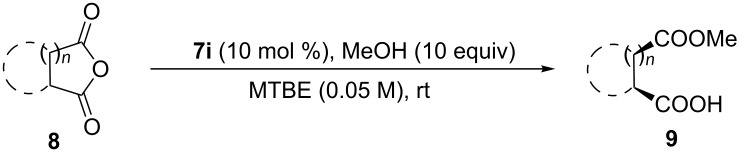					

Entry	Anhydride	Product	Time (h)	Yield^c^ (%)	ee^d^ (%)

1	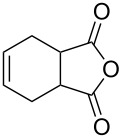 **8a**	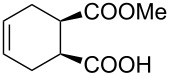 **9a**	17	98	95
2	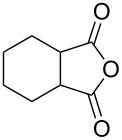 **8b**	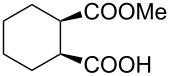 **9b**	60	97	90
3	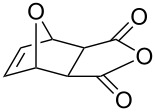 **8c**	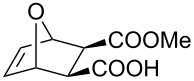 **9c**	66	94	73
4	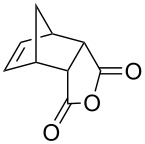 **8d**	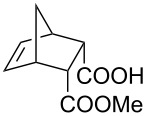 **9d**	66	95	84
5 *^e^*	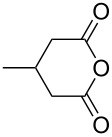 **8e**	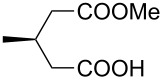 **9e**	84	87	81
6 *^e^*	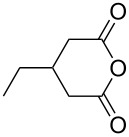 **8f**	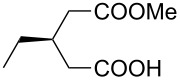 **9f**	84	91	80
7 *^e^*	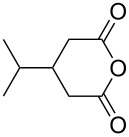 **8g**	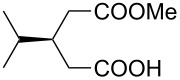 **9g**	84	90	94
8*^e^*	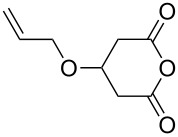 **8h**	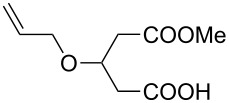 **9h**	84	97	81
9	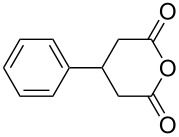 **8i**	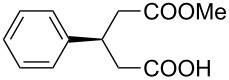 **9i**	60	89	77
10	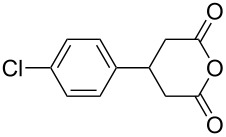 **8j**	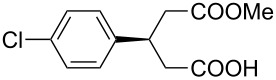 **9j**	72	87	78
11	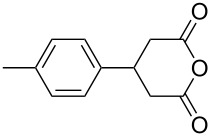 **8k**	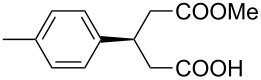 **9k**	84	92	79
12	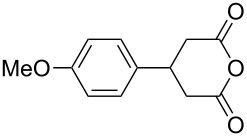 **8l**	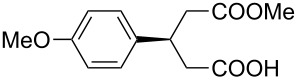 **9l**	96	91	80

^a^Unless otherwise noted, reactions were carried out with anhydride (0.5 mmol), MeOH (5.0 mmol), and **7i** (10 mol %) in MTBE (10 mL) at rt. ^b^Absolute configuration was determined by comparing with literature report. ^c^Isolated yield. ^d^Determined by HPLC after after derivatization. ^e^In MTBE (40 mL) at 0 °C.

The generality and scope of this methodology was further demonstrated in the alcoholysis of **8a** with different alcohols (Table 3). Excellent yields with high enantioselectivities were obtained in all cases. The sterically more bulky 2-propanol worked well in this reaction. Allylic alcohol, benzyl alcohol and cinnamyl alcohol were also well compatible in this bifunctional organocatalysis conditions to furnish the desired products.

**Table 3:** Asymmetric alcoholysis of anhydride **8a** with various alcohols.^a^

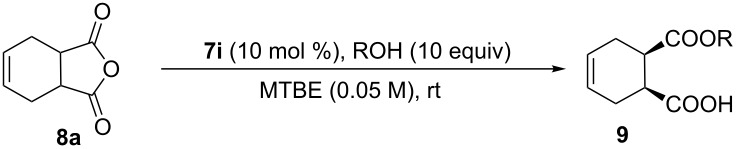					

Entry	ROH	Monoester	Time (h)	Yield^b^ (%)	ee^c,d^ (%)

1	methanol	**9a**	17	98	95
2	ethanol	**9m**	96	97	93
3	2-propanol	**9n**	98	93	89
4	allylic alcohol	**9o**	84	90	85
5	benzyl alcohol	**9p**	66	98	86
6	cinamyl alcohol	**9q**	66	99	90

^a^Unless otherwise noted, above reactions were carried out with anhydride (0.5 mmol), MeOH (5.0 mmol), and catalyst **7i** (10 mol %) in MTBE (40 mL) at rt. ^b^Isolated yield. ^c^Determined by HPLC after derivatization. ^d^Absolute configuration was determined by comparing with the literature report.

**Scheme 2:**
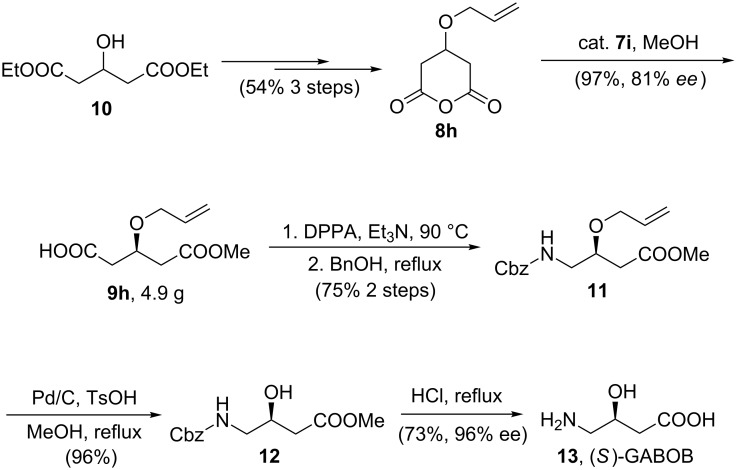
Asymmetric synthesis of (*S*)-GABOB (**13**).

Application of this methodology to the synthesis of (*S*)-GABOB (**13**), a metabolic derivative of the neurotransmitter γ-aminobutanoic acid, was performed to illustrate the synthetic utility [58–59] (Scheme 2). From commercially available diethyl 3-hydroxyglutarate (**10**), anhydride **8h** was prepared in three steps in 54% overall yield. The crucial chloramphenicol base amide **7i** catalyzed enantioselective alcoholysis gave monoester **9h** in multigram scale. The resulting monoester with the desired stereocenter at C-3 was converted to triprotected derivative of GABOB **11** via Curtius rearrangement in 75% yield over two steps. After Pd/C-catalyzed hydrogenative deprotection of hydroxy group in methanol under acidic conditions, the corresponding alcohol **12** was transferred to the product **13** via acid-promoted deprotection. A single recrystallization from ethanol allowed **13** to be isolated with 96% ee and 73% yield.

## Conclusion

In conclusion, we developed a unique bifunctional chiral organocatalyst via introducing a simple amide functionality at C-1 of the easily available chloramphenicol scaffold, which shows good catalytic reactivity and enantioselectivity in the alcoholytic desymmetrization of *meso*-cyclic anhydrides. This method proved to be generally applicable on various anhydrides or alcohols to generate the desired monoesters in good yield and enantioselectivity. The application of this method to the synthesis of (*S*)-GABOB demonstrated the great synthetic potential for pharmaceutically useful chemicals. Further modification of this chloramphenicol scaffold as well as total synthesis of other natural chemicals are right now ongoing in our lab.

## Experimental

### General procedure for the synthesis of **9**

Similarly as described in [37], with stirring under an atmosphere of nitrogen, alcohol (5 mmol, 10 equiv) was added dropwise to a solution of an anhydride **8** (0.5 mmol, 1 equiv) and **7i** (0.05 mmol, 10 mol %) in MTBE (20 mL) at room temperature. The reaction was monitored by using thin-layer chromatography. Once the reaction was completed, the solvent was evaporated under reduced pressure and the residue was dissolved in CH_2_Cl_2_ (10 mL). The solution was successively washed with saturated Na_2_CO_3_ (2 × 5 mL), acidified with excess 2 N HCl, followed by extraction with EtOAc (3 × 10 mL). The combined organic phase were dried over Na_2_SO_4_ and concentrated to afford the corresponding monoester, without further purification by flash chromatography.

## Supporting Information

File 1 Detailed experimental procedures, ^1^H NMR files.
