# Supplementary material for: Novel amide-functionalized chloramphenicol base bifunctional organocatalysts for enantioselective alcoholysis of meso-cyclic anhydrides
Source: Beilstein J Org Chem. 2018 Jan 31;14:309–17. doi: 10.3762/bjoc.14.19 (PMC5815282; doi:10.3762/bjoc.14.19)
Supplement: File 1 — Detailed experimental procedures, 1H NMR files. [file Beilstein_J_Org_Chem-14-309-s001.pdf]

Supporting Information

for

Novel amide-functionalized chloramphenicol base bifunctional organocatalysts for enantioselective alcoholysis of *meso*-cyclic anhydrides

Lingjun Xu, Shuwen Han, Linjie Yan, Haifeng Wang, Haihui Peng* and Fener Chen*

Address: Department of Chemistry, Fudan University, Shanghai 200433, PR China.

Email: Haihui Peng - haihui_peng@fudan.edu.cn; Fener Chen - rfchen@fudan.edu.cn

*Corresponding author

Detailed experimental procedures, ^{1}H NMR files

Content

1 General procedure...……………………………………………………….…….........S2

2. Preparation of the chloramphenicol base amide bifunctional organocatalysts..S2–11

3. Typical procedure for alcoholysis of *meso*-cyclic anhydride..............................S11

4. Scale-up methanolysis of *meso*-cyclic anhydride **8h** (4.26 g scale)…………......S11

5. Characterization of the monoesters…..………...…………………………….S12–18

6. Catalytic asymmetric synthesis of (*S*)-GABOB by **7i**………………….……S19–23

7. References.………………..…………………………………………………...S23

8. Copies of NMR spectra and chiral HPLC spectra……………..........………S24–79

1. General procedure

Unless otherwise specified, all reagents and solvents were purchased from commercial sources and used as received. ^{1}H (400 MHz) and ^{13}C (100 MHz) NMR were recorded on a Bruker Avance 400 spectrometer in $CDCl_3$ or d_6-DMSO using tetramethylsilane (TMS) as internal standards. Coupling constant (*J*) values are given in Hz. Multiplicities are designated by the following abbreviations: s, singlet; d, doublet; t, triplet; q, quartet; br, broad; m, multiplet. Melting points were measured on WRS-1B digital melting-point apparatus. Products were purified by flash column chromatography on silica gel purchased from Qingdao Haiyang Chemical Co. Ltd. Optical rotations were measured by a Rudolph AUTOPOL I Automatic Polarimeter. EIMS were recorded on an Agilent 6890N/5975 spectrometer and ESI-MS were recorded on a Waters Micromass Quattro Micro spectrometer. HRMS were recorded on a Bruker micrOTOF spectrometer. HPLC analysis were performed with Daicel Chiralpak AD-H column (25 cm × 4.6 mm × 5 μm), Chiralpak OD-H column (25 cm × 4.6 mm × 5 μm) and Chiralpak IA-H column (25 cm × 4.6 mm × 5 μm).

2. Preparation of the chloramphenicol base amide bifunctional organocatalysts

General procedure

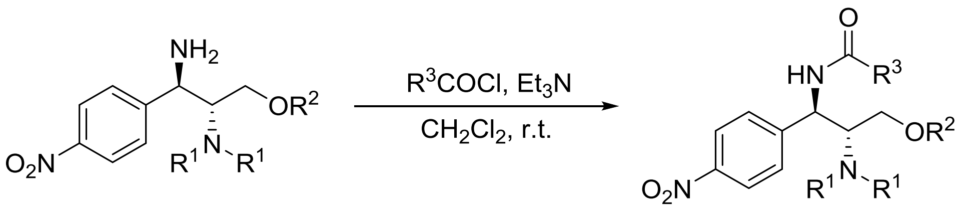

7

To a solution of chloramphenicol base (1 g, 2 mmol) and in CH_2Cl_2 (20 mL) was added the solution of Et_3N (1.15 mL, 8 mmol) in CH_2Cl_2 (10 mL) under N_2 atmosphere. After cooling to 0 °C, R^3COCl (3 mmol) was added dropwise over 20 min. After addition, the reaction mixture was stirred for 3 h at room temperature and then quenched by water (10 mL). The organic phase was washed with $NaHCO_3$ (20 mL), H_2O (20 mL), brine (20mL), dried over Na_2SO_4 and concentrated under reduced

pressure to give a yellow solid. The crude product was purified by flash chromatography using PE/EA 10:1 to give product **7**.

_N_-((1_R_,2_R_)-2-(Dimethylamino)-1-(4-nitrophenyl)-3-(trityloxy)propyl)-4-(tri-fluoromethyl)benzamide (7a)

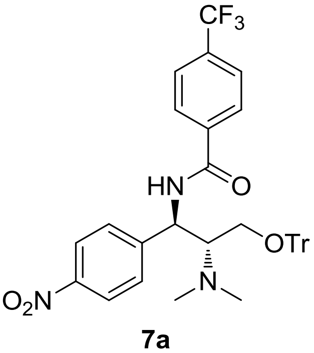

7a

Yellow solid, yield 80%; ^{1}H NMR (400 MHz, $CDCl_3$) δ = 8.20 (s, 1H), 8.06 (d, *J* = 8.6 Hz, 2H), 7.90 (d, *J* = 8.1 Hz, 2H), 7.72 (d, *J* = 8.2 Hz, 2H), 7.37 (d, *J* = 8.5 Hz, 2H), 7.28-7.23 (m, 15H), 4.69 (d, *J* = 10.3 Hz, 1H), 3.30-3.22 (m, 2H), 3.01-2.97 (m, 1H), 2.46 (s, 6H) ppm; ^{13}C NMR (100 MHz, $CDCl_3$) δ = 165.9, 148.8, 147.0, 143.0, 137.1, 133.2, 128.5, 128.2, 127.7, 127.4, 127.1, 125.6, 125.6, 123.6, 87.6, 67.7, 58.17, 54.1, 41.4 ppm; HRMS (ESI$^+$) calcd for $C_{38}H_{34}F_3N_3O_4$ $[M+H]^+$ = 654.2580, found: 654.2574.

_N_-((1_R_,2_R_)-2-(Dimethylamino)-1-(4-nitrophenyl)-3-(trityloxy)propyl)-4-nitro-benzamide (7b)

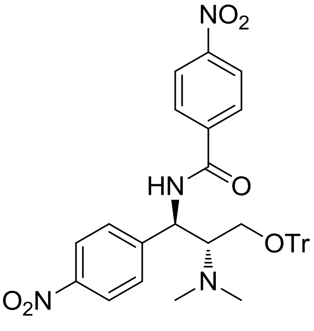

7b

Yellow solid, yield 73%; ^{1}H NMR (400 MHz, $CDCl_3$) δ = 8.30 (d, *J* = 8.7 Hz, 2H), 8.26 (s, 1H), 8.07 (d, *J* = 8.6 Hz, 2H), 7.94 (d, *J* = 8.7 Hz, 2H), 7.37 (d, *J* = 8.6 Hz, 2H), 7.26-7.23 (m, 15H), 4.69 (dd, *J* = 10.2, 1.9 Hz, 1H), 3.31-3.22 (m, 2H), 3.01-2.96 (m, 1H), 2.47 (s, 6H) ppm; ^{13}C NMR (100 MHz, $CDCl_3$) δ = 165.2, 149.7,

148.5, 147.0, 143.0, 139.4, 128.5, 128.2, 128.1, 127.7, 127.2, 123.8, 123.7, 87.6, 67.6, 58.1, 54.2, 41.4 ppm; HRMS (ESI$^+$) calcd for $C_{37}H_{34}N_4O_6$ $[M+H]^+$ = 631.2557, found: 631.2568.

_N_-((1_R_,2_R_)-2-(Dimethylamino)-1-(4-nitrophenyl)-3-(trityloxy)propyl)-4-fluoro-benzamide (7c)

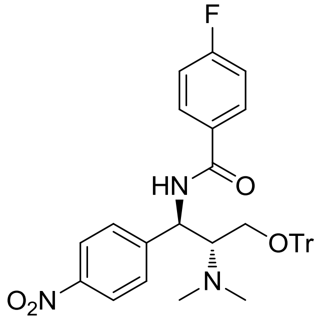

7c

Yellow solid, yield 85%; ^{1}H NMR (400 MHz, $CDCl_3$) δ = 8.12 (s, 1H), 8.06 (d, *J* = 8.6 Hz, 2H), 7.82-7.78 (m, 2H), 7.38 (d, *J* = 8.6 Hz, 2H), 7.25 (m, 15H), 7.11 (t, *J* = 8.6 Hz, 2H), 4.70 (d, *J* = 10.3 Hz, 1H), 3.29-3.22 (m, 2H), 3.02-2.99 (m, 1H), 2.46 (s, 6H) ppm; ^{13}C NMR (100 MHz, $CDCl_3$) δ = 166.2, 149.1, 146.9, 143.0, 129.4, 129.3, 128.5, 128.3, 127.7, 127.1, 123.6, 115.7, 115.4, 87.6, 67.6, 58.1, 53.9, 41.3 ppm; HRMS (ESI$^+$) calcd for $C_{37}H_{34}FN_3O_4$ $[M+H]^+$ = 604.2612, found: 604.2625.

4-Chloro-_N_-((1_R_,2_R_)-2-(dimethylamino)-1-(4-nitrophenyl)-3-(trityloxy)propyl)-benzamide (7d)

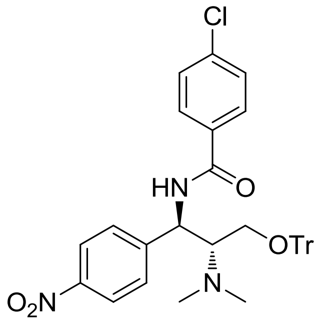

7d

Yellow solid, yield 80%; ^{1}H NMR (400 MHz, $CDCl_3$) δ = 8.13 (s, 1H), 8.06 (d, *J* = 8.6 Hz, 2H), 7.73 (d, *J* = 8.5 Hz, 2H), 7.42 (d, *J* = 8.6 Hz, 2H), 7.37 (d, *J* = 8.6 Hz, 2H), 7.28-7.23 (m, 15H), 4.69 (d, *J* = 8.7 Hz, 1H), 3.29-3.21 (m, 2H), 3.02-2.97 (m, 1H), 2.46 (s, 6H) ppm; ^{13}C NMR (100 MHz, $CDCl_3$) δ = 166.2, 149.0, 146.9, 143.0, 137.9, 132.2, 128.8, 128.5, 128.4, 129.2, 127.7, 127.1, 123.6, 87.6, 67.6, 58.1, 54.0,

41.3 ppm; HRMS (ESI$^+$) calcd for $C_{37}H_{34}ClN_3O_4$ $[M+H]^+$ = 620.2316, found: 620.2305.

4-Bromo-*N*-((1*R*,2*R*)-2-(dimethylamino)-1-(4-nitrophenyl)-3-(trityloxy)propyl)-benzamide (7e)

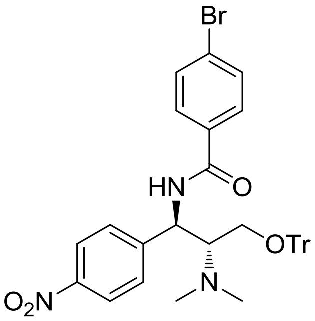

7e

Yellow solid, yield 88%; ^{1}H NMR (400 MHz, $CDCl_3$) δ = 8.09 (s, 1H), 8.05 (d, *J* = 8.6 Hz, 2H), 7.66 (d, *J* = 8.4 Hz, 2H), 7.58 (d, *J* = 8.4 Hz, 2H), 7.36 (d, *J* = 8.5 Hz, 2H), 7.27-7.23 (m, 15H), 4.66 (d, *J* = 10.1 Hz, 1H), 3.29-3.20 (m, 2H), 2.99-2.95 (m, 1H), 2.45 (s, 6H) ppm; ^{13}C NMR (100 MHz, $CDCl_3$) δ = 166.3, 149.0, 146.9, 143.0, 132.7, 131.8, 128.6, 128.5, 128.2, 127.7, 127.1, 126.4, 123.6, 87.6, 67.7, 58.1, 54.0, 41.4 ppm; HRMS (ESI$^+$) calcd for $C_{37}H_{34}BrN_3O_4$ $[M+H]^+$ = 664.1811, found: 664.1809.

***N*-((1*R*,2*R*)-2-(Dimethylamino)-1-(4-nitrophenyl)-3-(trityloxy)propyl)benzamide (7f)**

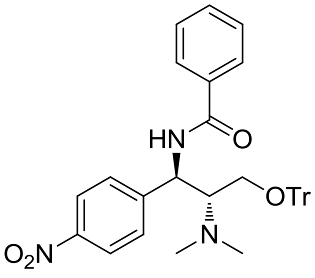

7f

Yellow solid, yield 75%; ^{1}H NMR (400 MHz, $CDCl_3$) δ = 8.15 (s, 1H), 8.06 (d, *J* = 8.6 Hz, 2H), 7.80 (d, *J* = 7.3 Hz, 2H), 7.52 (d, *J* = 7.4 Hz, 1H), 7.46 (t, *J* = 7.6 Hz, 2H), 7.39 (d, *J* = 8.7 Hz, 2H), 7.27-7.23 (m, 15H), 4.72 (dd, *J* = 10.4, 2.3 Hz, 1H), 3.30-3.21 (m, 2H), 3.03-2.99 (m, 1H), 2.47 (s, 6H) ppm; ^{13}C NMR (100 MHz, $CDCl_3$) δ = 167.2, 149.3, 146.8, 143.1, 133.9, 131.6, 128.5, 128.3, 127.7, 127.1, 127.0, 123.6,

87.5, 67.7, 58.2, 53.9, 41.3 ppm; HRMS (ESI$^+$) calcd for $C_{37}H_{35}N_3O_4$ $[M+H]^+$ = 586.2706, found: 586.2705.

***N*-((1*R*,2*R*)-2-(Dimethylamino)-1-(4-nitrophenyl)-3-(trityloxy)propyl)-4-methyl-benzamide (7g)**

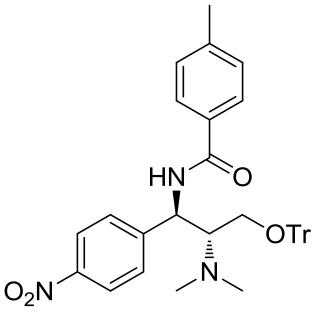

7g

Yellow solid, yield 77%; ^{1}H NMR (400 MHz, $CDCl_3$) δ = 8.12 (s, 1H), 8.05 (d, *J* = 8.5 Hz, 2H), 7.70 (d, *J* = 8.0 Hz, 2H), 7.39 (d, *J* = 8.6 Hz, 2H), 7.27-7.23 (m, 17H), 4.72 (d, *J* = 10.4 Hz, 1H), 3.28 – 3.21 (m, 2H), 3.03 – 3.01 (m, 1H), 2.46 (s, 6H), 2.41 (s, 3H) ppm; ^{13}C NMR (100 MHz, $CDCl_3$) δ = 167.3, 149.4, 146.8, 143.1, 142.1, 131.0, 129.2, 128.5, 128.3, 127.7, 127.1, 127.0, 123.6, 87.5, 67.6, 58.2, 53.8, 41.3, 21.4 ppm; HRMS (ESI$^+$) calcd for $C_{38}H_{37}N_3O_4$ $[M+H]^+$ = 600.2862, found: 600.2859.

***N*-((1*R*,2*R*)-2-(Dimethylamino)-1-(4-nitrophenyl)-3-(trityloxy)propyl)-4-methoxy-benzamide (7h)**

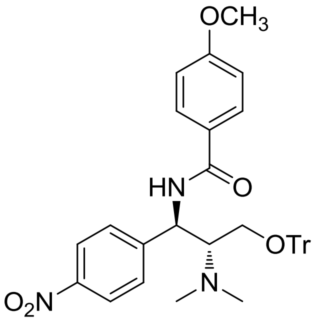

7h

Yellow solid, yield 68%; ^{1}H NMR (400 MHz, $CDCl_3$) δ = 8.02 (d, *J* = 8.6 Hz, 2H), 7.98 (s, 1H), 7.74 (d, *J* = 8.6 Hz, 2H), 7.35 (d, *J* = 8.5 Hz, 2H), 7.24-7.21 (m, 15H), 6.92 (d, *J* = 8.6 Hz, 2H), 4.65 (d, *J* = 10.3 Hz, 1H), 3.84 (s, 3H), 3.26-3.18 (m, 2H), 2.96-2.93 (m, 1H), 2.43 (s, 6H) ppm; ^{13}C NMR (100 MHz, $CDCl_3$) δ = 167.1, 162.4, 148.7, 147.1, 142.9, 129.0, 128.7, 128.6, 127.9, 127.3, 126.1, 123.8, 113.7, 87.7, 66.7,

57.9, 55.3, 53.3, 41.0 ppm; HRMS (ESI$^+$) calcd for $C_{38}H_{37}N_3O_5$ $[M+H]^+$ = 616.2811, found: 616.2804.

***N*-((1*R*,2*R*)-2-(Dimethylamino)-1-(4-nitrophenyl)-3-(trityloxy)propyl)-3,5-bis-(trifluoromethyl)benzamide (7i)**

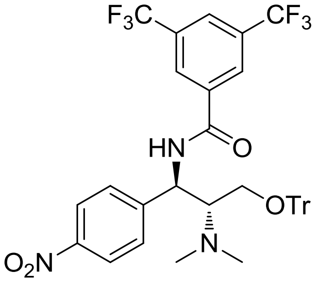

7i

White solid, yield 90%; ^{1}H NMR (400 MHz, $CDCl_3$) δ = 8.26 (br, 1H), 8.20 (s, 2H), 8.06 (d, *J* = 8.6 Hz, 2H), 8.02 (s, 1H), 7.38 (d, *J* = 8.6 Hz, 2H), 7.24-7.23 (m, 15H), 4.70 (d, *J* = 10.7 Hz, 1H), 3.30-3.22 (m, 2H), 3.04-3.03 (m, 1H), 2.47 (s, 6H) ppm; ^{13}C NMR (100 MHz, $CDCl_3$) δ = 164.5, 148.3, 147.1, 143.0, 136.0, 132.4, 132.0, 128.5, 128.3, 127.7, 127.2, 127.2, 123.7, 87.6, 67.6, 60.3, 54.2, 41.3 ppm; HRMS (ESI$^+$) calcd for $C_{39}H_{33}F_6N_3O_4$ $[M+H]^+$ = 722.2454, found: 722.2455.

***N*-((1*R*,2*R*)-1-(4-Nitrophenyl)-2-(pyrrolidin-1-yl)-3-(trityloxy)propyl)-3,5-bis-(trifluoromethyl)benzamide (7j)**

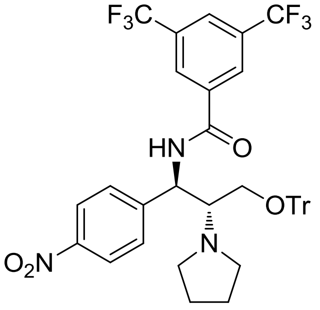

7j

Light yellow solid, yield 92%; ^{1}H NMR (400 MHz, $CDCl_3$) δ = 8.37 (br, 1H), 8.16 (s, 2H), 8.08 (d, *J* = 8.4 Hz, 2H), 7.99 (s, 1H), 7.41 (d, *J* = 8.3 Hz, 2H), 7.21-7.19 (m, 15H), 5.05 (d, *J* = 5.5 Hz, 1H), 3.36-3.33 (m, 1H), 3.26-3.20 (m, 2H), 2.64 (d, *J* = 28.8 Hz, 4H), 1.73-1.69 (m, 4H) ppm; ^{13}C NMR (100 MHz, $CDCl_3$) δ = 164.4, 148.6, 147.2, 143.2, 136.4, 132.5, 132.2, 128.6, 128.1, 128.0, 127.4, 124.3, 123.8, 121.6,

87.8, 65.1, 60.1, 54.6, 49.7, 23.7 ppm; HRMS (ESI$^+$) calcd for $C_{41}H_{35}F_6N_3O_4$ $[M+H]^+$ = 748.2610, found: 748.2592.

***N*-((1*R*,2*R*)-1-(4-Nitrophenyl)-2-(piperidin-1-yl)-3-(trityloxy)propyl)-3,5-bis-(trifluoromethyl)benzamide (7k)**

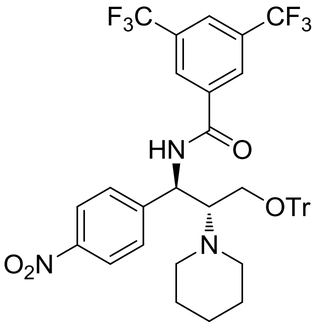

7k

Light yellow solid, yield 89%; ^{1}H NMR (400 MHz, $CDCl_3$) δ = 8.72 (s, 1H), 8.29 (s, 2H), 8.07-8.02 (m, 3H), 7.36 (d, J = 8.2 Hz, 2H), 7.25-7.21 (m, 15H), 4.60 (d, *J* = 10.4 Hz, 1H), 3.34-3.30 (m, 1H), 3.24 (d, *J* = 10.3 Hz, 1H), 2.94 (s, 1H), 2.80-2.76 (m, 2H), 2.60 (s, 2H), 1.63 (s, 2H), 1.51 (s, 4H) ppm; ^{13}C NMR (100 MHz, $CDCl_3$) δ = 163.8, 148.7, 147.2, 143.2, 136.0, 132.6, 132.4, 128.7, 128.5, 127.9, 127.3, 125.2, 124.5, 123.9, 87.8, 69.1, 58.8, 53.6, 27.4, 27.0, 24.5 ppm; HRMS (ESI$^+$) calcd for $C_{42}H_{37}F_6N_3O_4$ $[M+H]^+$ = 762.2767, found: 762.2731.

***N*-((1*R*,2*R*)-2-Morpholino-1-(4-nitrophenyl)-3-(trityloxy)propyl)-3,5-bis(tri-fluoromethyl)benzamide (7l)**

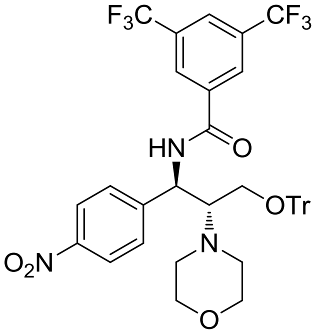

7l

Yellow solid, yield 82%; ^{1}H NMR (400 MHz, $CDCl_3$) δ = 8.38 (s, 1H), 8.26 (s, 2H), 8.07 (s, 1H), 8.04 (s, 2H), 7.35 (d, *J* = 8.4 Hz, 2H), 7.25-7.21 (m, 15H), 4.70 (d, *J* = 10.4 Hz, 1H), 3.76-3.73 (m, 2H), 3.73-3.68 (m, 2H), 3.35-3.31 (m, 1H), 3.28-3.26 (m,

1H), 3.00-2.98 (m, 1H), 2.85 (m, 2H), 2.72-2.71 (m, 2H) ppm; ^{13}C NMR (100 MHz, $CDCl_3$) δ = 163.8, 148.1, 147.3, 143.1, 135.8, 132.4, 128.6, 128.4, 127.9, 127.4, 127.2, 124.3, 123.9, 88.0, 68.4, 68.0, 58.9, 53.3, 27.0 ppm; HRMS (ESI$^+$) calcd for $C_{41}H_{35}F_6N_3O_5$ $[M+Na]^+$ = 786.2379, found: 786.2349.

***N*-((1*R*,2*R*)-3-((*tert*-Butyldimethylsilyl)oxy)-2-(dimethylamino)-1-(4-nitrophenyl)-propyl)-3,5-bis(trifluoromethyl)benzamide (7m)**

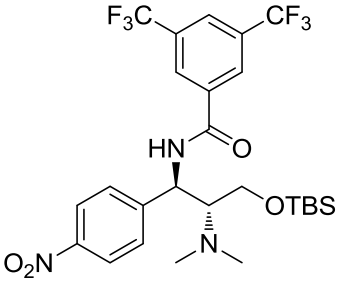

7m

White solid, yield 88%; ^{1}H NMR (400 MHz, $CDCl_3$) δ = 8.23-8.20 (m, 4H), 8.00 (s, 1H), 7.64 (d, *J* = 8.3 Hz, 2H), 5.10 (d, *J* = 9.5 Hz, 1H), 3.83 (d, *J* = 11.4 Hz, 1H), 3.37 (dd, *J* = 11.4, 4.1 Hz, 1H), 2.51 (s, 6H), 1.26 (s, 1H), 0.91 (s, 9H), 0.01 (s, 6H) ppm; ^{13}C NMR (100 MHz, $CDCl_3$) δ = 164.8, 147.5, 136.3, 132.5, 132.2, 128.6, 127.5, 125.3, 123.9, 121.6, 68.2, 56.7, 52.9, 41.8, 25.9, 18.2, -5.5 ppm; HRMS (ESI$^+$) calcd for $C_{26}H_{33}F_6N_3O_4Si$ $[M+H]^+$ = 594.2223, found: 594.2209.

***N*-((1*R*,2*R*)-3-((*tert*-Butyldimethylsilyl)oxy)-1-(4-nitrophenyl)-2-(pyrrolidin-1-yl)-propyl)-3,5-bis(trifluoromethyl)benzamide (7n)**

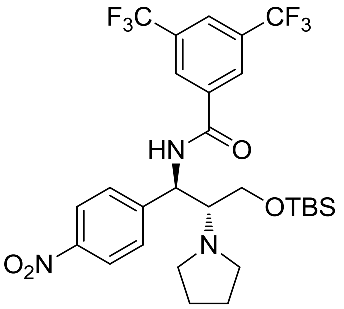

7n

Yellow solid, yield 85%; ^{1}H NMR (400 MHz, $CDCl_3$) δ = 8.39 (s, 1H), 8.24 (s, 2H), 8.20 (d, *J* = 8.1 Hz, 2H), 8.03 (s, 1H), 7.60 (d, *J* = 8.0 Hz, 2H), 5.29 (s, 1H), 3.71-3.63 (m, 2H), 3.04 (s, 1H), 2.80 (s, 2H), 2.68 (s, 2H), 1.83-1.77 (m, 4H), 0.88 (s, 9H), 0.01 (s, 6H) ppm; ^{13}C NMR (100 MHz, $CDCl_3$) δ = 164.1, 148.5, 147.2, 136.3, 132.4,

132.1, 127.9, 127.3, 123.6, 121.5, 66.5, 59.8, 53.5, 50.2, 25.8, 23.5, 18.1, -5.6 ppm; HRMS (ESI$^+$) calcd for $C_{28}H_{35}F_6N_3O_4Si$ $[M+H]^+$ = 620.2379, found: 620.2377.

***N*-((1*R*,2*R*)-3-((*tert*-Butyldimethylsilyl)oxy)-1-(4-nitrophenyl)-2-(piperidin-1-yl)-propyl)-3,5-bis(trifluoromethyl)benzamide (7o)**

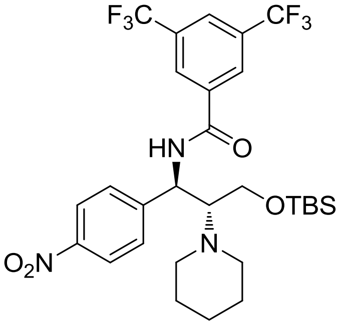

7o

Light yellow solid, yield 83%; ^{1}H NMR (400 MHz, $CDCl_3$) δ = 8.68 (s, 1H), 8.31 (s, 2H), 8.20 (d, *J* = 8.1 Hz, 2H), 8.03 (s, 1H), 7.60 (d, *J* = 8.1 Hz, 2H), 4.94 (d, *J* = 10.3 Hz, 1H), 3.79 (d, *J* = 11.2 Hz, 1H), 3.42 (dd, *J* = 11.2, 4.3 Hz, 1H), 2.86-2.81 (m, 2H), 2.74 (d, *J* = 8.8 Hz, 1H), 2.62 (s, 2H), 1.66 (s, 2H), 1.54 (s, 4H), 0.90 (s, 9H), 0.01 (s, 6H) ppm; ^{13}C NMR (100 MHz, $CDCl_3$) δ = 164.0, 149.0, 147.4, 136.1, 132.6, 132.2, 128.4, 127.3, 125.3, 123.9, 121.7, 70.0, 57.3, 52.2, 27.5, 25.9, 24.6, 18.1, -5.5 ppm; HRMS (ESI$^+$) calcd for $C_{29}H_{37}F_6N_3O_4Si$ $[M+H]^+$ = 634.2536, found: 634.2521.

***N*-((1*R*,2*R*)-3-((*tert*-Butyldimethylsilyl)oxy)-2-morpholino-1-(4-nitrophenyl)-propyl)-3,5-bis(trifluoromethyl)benzamide (7p)**

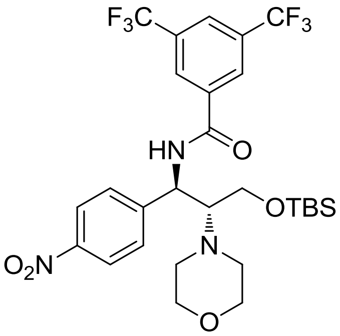

7p

Light yellow solid, yield 80%; ^{1}H NMR (400 MHz, $CDCl_3$) δ = 8.35 (s, 1H), 8.28 (s, 2H), 8.21 (d, *J* = 8.0 Hz, 2H), 8.04 (s, 1H), 7.61 (d, *J* = 8.1 Hz, 2H), 5.07 (d, *J* = 10.2 Hz, 1H), 3.84 (d, *J* = 11.4 Hz, 1H), 3.76 (d, *J* = 6.0 Hz, 2H), 3.68 (s, 2H), 3.41-3.38 (m, 1H), 2.87 (s, 2H), 2.79 (d, *J* = 10.1 Hz, 1H), 2.74-2.70 (m, 2H), 0.91 (s, 9H), 0.02

(s, 6H) ppm; ^{13}C NMR (100 MHz, $CDCl_3$) δ = 164.1, 148.3, 147.5, 136.0, 132.7, 132.3, 128.5, 127.2, 123.9, 121.6, 69.2, 68.0, 57.2, 52.0, 50.4, 25.9, 18.1, -5.5 ppm; HRMS (ESI$^+$) calcd for $C_{28}H_{35}F_6N_3O_5Si$ $[M+Na]^+$ = 658.2148, found: 658.2124.

3. Typical procedure for alcoholysis of *meso*-cyclic anhydride

An alcohol (5 mmol) was added dropwise at room temperature under nitrogen to a stirred solution of an anhydride **8** (0.5 mmol) and **7i** (36.1 mg, 0.05 mmol) in MTBE (20 mL). The reaction was monitored by using thin-layer chromatography. Once anhydride consumption was complete, the solvent was evaporated under reduced pressure and the residue was dissolved in CH_2Cl_2 (10 mL). The solution was washed with saturated Na_2CO_3 (2 × 5 mL) and the combined aqueous phase were acidified with excess 2 N HCl, followed by extraction with EtOAc (3 × 10 mL). The combined organic phases were dried over Na_2SO_4 and concentrated to afford the corresponding monoester, without further purification by flash chromatography.

4. Scale-up methanolysis of *meso*-cyclic anhydride 8h (4.26 g scale)

MeOH (10.1 mL, 250 mmol) was added dropwise at 0 °C under nitrogen to a stirred solution of *meso*-cyclic anhydride **8h** (4.26 g, 25 mmol) and **7i** (1.80 g, 2.5 mmol) in MTBE (2 L). The reaction was monitored by using thin-layer chromatography. After 96 h, the anhydride consumption was complete. The solvent was evaporated under reduced pressure and the residue was dissolved in CH_2Cl_2 (300 mL). The solution was washed with saturated Na_2CO_3 (3 × 150 mL) and the combined aqueous layers were acidified with excess 2 N HCl, followed by extraction with EtOAc (3 × 300 mL). The combined organic layers were dried over Na_2SO_4 and concentrated to afford the corresponding monoester **9h**, without further purification by flash chromatography. Monoester **9h**, light yellow oil, yield 97%, 81% ee; $[\alpha]_D^{25}$ = -3.5 (*c* = 1.0 in $CHCl_3$); Chiral HPLC (Chiralcel AD-H column), Hexane/*i*-PrOH = 95/5, Flow rate: 0.6 mL/min, UV detection at 220 nm, T = 30 °C, retention time: t(minor) = 50.6 min, t(major) = 56.1 min.

5. Characterization of the monoesters

All monoesters except **9h** are known compounds and their NMR spectra data were identical to those reported in the literature.[2-7] The enantiomeric excess of the monoester was determined by chiral HPLC, analysis of the diastereoisomeric mixture of the corresponding amide ester derived from (*S*)-1-phenylethylamine according to the reported procedure.[2]

Thionyl chloride (0.6 mmol, 45 μL) was added to a solution of the momoester (0.5 mmol) in dry toluene (10 mL) at 0 °C. The mixture was stirred at 0 °C for 30 minutes, and triethylamine (1.5 mmol, 0.14 mL) and (*S*)-1- phenylethylamine (0.55 mmol, 71 μL) were added successively. The mixture was stirred at 0 °C for 1 h and at room temperature for an additional 1 h. The residue was then dissolved in ethyl acetate (50 mL). The organic solution was washed with HCl (2 N, 50 mL), saturated aq $NaHCO_3$ (50 mL), water (50 mL) and brine (50 mL). The organic phase was dried over Na_2SO_4 and concentrated in vacuo to give the diastereoisomeric mixture. The enantiomeric excess was determined by comparison of chiral HPLC.

(1*S*,6*R*)-6-(Methoxycarbonyl)cyclohex-3-enecarboxylic acid (9a)

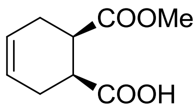

9a

White solid, yield 98%, 95% *ee*; $[\alpha]_D^{25}$ = -4.3 (*c* = 1.0 in $CHCl_3$) (lit.[2] $[\alpha]_D^{20}$ = -4.9 (*c* = 1.5 in $CHCl_3$)); Chiral HPLC (Chiralcel OD-H column), Hexane/*i*-PrOH = 93/7, Flow rate: 0.5 mL/min, UV detection at 220 nm, T = 30°C, retention time: t(major) = 33.1 min, t(minor) = 45.3 min; ^{1}H NMR (400 MHz, $CDCl_3$) δ = 4.93 (dd, *J* = 24.1, 3.6 Hz, 2H), 3.67 (s, 3H), 3.05-2.99 (m, 2H), 1.84-1.82 (m, 2H), 1.56-1.51 (m, 2H) ppm; ^{13}C NMR (100 MHz, $CDCl_3$) δ = 179.7, 173.7, 125.1, 125.0, 51.9, 39.6, 39.4, 25.7, 25.5 ppm.

(1*S*,2*R*)-2-(Methoxycarbonyl)cyclohexanecarboxylic acid (9b)

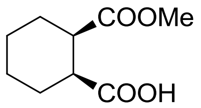

9b

Colorless oil, yield 97%, 90% *ee*; $[\alpha]_D^{25}$ = +3.6 (*c* = 1.0 in $CHCl_3$) (lit.[2] $[\alpha]_D^{20}$ = +3.5 (*c* = 1.43 in $CHCl_3$)); Chiral HPLC (Chiralcel AD-H column), Hexane/*i*-PrOH = 93/7, Flow rate: 0.5 mL/min, UV detection at 220 nm, T = 30°C, retention time: t(minor) = 23.2 min, t(major) = 29.9 min; ^{1}H NMR (400MHz, DMSO): δ = 3.67 (s, 3H), 2.84 (s, 2H), 2.00 (br, 2H), 1.78 (br, 2H), 1.54-1.47 (m, 2H), 1.42-1.39 (m, 2H) ppm; ^{13}C NMR (100 MHz, DMSO) δ = 180.2, 174.2, 51.8, 42.6, 42.4, 26.3, 26.0, 23.8, 23.7 ppm.

(1*S*,2*R*,3*S*,4*R*)-3-(Methoxycarbonyl)-7-oxabicyclo[2.2.1]hept-5-ene-2-carboxylic acid (9c)

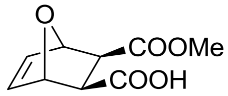

9c

White solid, yield 94%, 73% *ee*; $[\alpha]_D^{25}$ = +6.3 (*c* = 1.0 in $CHCl_3$) (lit.[2] $[\alpha]_D^{20}$ = +8.7 (*c* = 1.08 in $CHCl_3$)); Chiral HPLC (Chiralcel OD-H column), Hexane/*i*-PrOH = 85/15, Flow rate: 0.5 mL/min, UV detection at 220 nm, T = 30°C, retention time: t(major) = 23.8 min, t(minor) = 37.8 min; ^{1}H NMR (400 MHz, $CDCl_3$) δ = 7.69 (br, 1H), 6.49-6.45 (m, 2H), 5.29 (d, *J* = 16.7 Hz, 2H), 3.71 (s, 3H), 2.88-2.83 (m, 2H) ppm; ^{13}C NMR (100 MHz, DMSO) δ = 173.0, 172.5, 137.1, 137.0, 80.4, 80.1, 51.9, 47.0, 46.3 ppm.

(1*R*,2*S*,3*R*,4*S*)-3-(Methoxycarbonyl)bicyclo[2.2.1]hept-5-ene-2-carboxylic acid (9d)

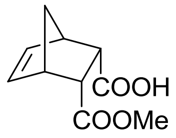

9d

White solid, yield 95%, 84% *ee*; $[\alpha]_D^{25}$ = -5.8 (*c* = 1.0 in $CHCl_3$) (lit.[2] $[\alpha]_D^{20}$ = -7.4 (*c* = 1.53 in $CHCl_3$)); Chiral HPLC (Chiralcel AD-H column), Hexane/*i*-PrOH = 96/4, Flow rate: 0.5 mL/min, UV detection at 220 nm, T = 30°C, retention time: t(minor)

= 47.8 min, t(major) = 50.7 min; ^{1}H NMR (400 MHz, $CDCl_3$) δ = 6.31 (dd, *J* = 5.1, 2.9 Hz, 1H), 6.22 (dd, *J* = 5.3, 2.8 Hz, 1H), 3.59 (s, 3H), 3.35-3.26 (m, 2H), 3.18 (d, *J* = 12.9 Hz, 2H), 1.49 (d, *J* = 8.6 Hz, 1H), 1.34 (d, *J* = 8.6 Hz, 1H) ppm; ^{13}C NMR (100 MHz, $CDCl_3$) δ = 178.3, 172.9, 135.5, 134.3, 51.5, 48.7, 48.2, 48.0, 46.5, 46.1 ppm.

(*R*)-5-Methoxy-3-methyl-5-oxopentanoic acid (9e)

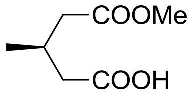

9e

Yellow oil, yield 87%, 81% *ee*; $[\alpha]_D^{25}$ = +3.1 (*c* = 1.0 in $CHCl_3$) (lit.[2] $[\alpha]_D^{20}$ = +1.1 (*c* = 1.36 in $CHCl_3$)); Chiral HPLC (Chiralcel AD-H column), Hexane/*i*-PrOH = 96/4, Flow rate: 0.5 mL/min, UV detection at 220 nm, T = 30°C, retention time: t(minor) = 38.3 min, t(major) = 44.1 min; ^{1}H NMR (400 MHz, $CDCl_3$) δ = 3.67 (s, 3H), 2.50-2.39 (m, 3H), 2.31-2.24 (m, 2H), 1.05 (d, *J* = 6.3 Hz, 3H) ppm; ^{13}C NMR (100 MHz, DMSO) δ = 173.8, 172.8, 51.6, 40.6, 40.3, 27.3, 19.8 ppm.

(*R*)-3-Ethyl-5-methoxy-5-oxopentanoic acid (9f)

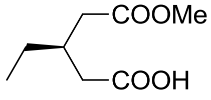

9f

Yellow oil, yield 91%, 80% *ee*; $[\alpha]_D^{25}$ = -1.36 (*c* = 1.0 in $CHCl_3$) (lit.[8] $[\alpha]_D^{20}$ = -0.92 (*c* = 1.0 in $CHCl_3$)); Chiral HPLC (Chiralcel AD-H column), Hexane/*i*-PrOH = 94/6, Flow rate: 0.5 mL/min, UV detection at 220 nm, T = 30°C, retention time: t(minor) = 26.7 min, t(major) = 30.3 min; ^{1}H NMR (400 MHz, $CDCl_3$) δ = 3.67 (s, 3H), 2.39 (t, *J* = 7.3 Hz, 4H), 2.31-2.24 (m, 1H), 1.42 (p, *J* = 7.3 Hz, 2H), 0.92 (t, *J* = 7.4 Hz, 3H) ppm; ^{13}C NMR (100 MHz, DMSO) δ = 174.0, 173.0, 51.6, 38.1, 37.8, 33.4, 26.4, 11.1 ppm.

(*R*)-3-Isopropyl-5-methoxy-5-oxopentanoic acid (9g)

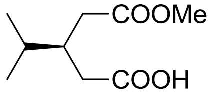

9g

Yellow oil, yield 90%, 94% *ee*; $[\alpha]_D^{25}$ = -2.7 (*c* = 1.0 in $CHCl_3$); Chiral HPLC (Chiralcel IA-H column), Hexane/*i*-PrOH = 94/6, Flow rate: 0.5 mL/min, UV detection at 220 nm, T = 30°C, retention time: t(minor) = 24.4 min, t(major) = 31.0 min; ^{1}H NMR (400 MHz, $CDCl_3$) δ = 10.97 (br, 1H), 2.44 (d, *J* = 12.4 Hz, 3H), 2.37-2.30 (m, 2H), 1.67-1.60 (m, 1H), 1.23 (t, *J* = 6.6 Hz, 2H), 0.91 (d, *J* = 6.4 Hz, 6H) ppm; ^{13}C NMR (100 MHz, $CDCl_3$) δ = 179.4, 178.1, 43.9, 38.9, 29.8, 25.1, 22.4, 20.8 ppm.

(*R*)-3-(Allyloxy)-5-methoxy-5-oxopentanoic acid (9h)

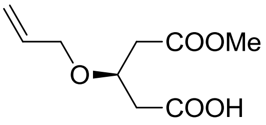

9h

Light yellow oil, yield 97%, 81% *ee*; $[\alpha]_D^{25}$ = -3.5 (*c* = 1.0 in $CHCl_3$); Chiral HPLC (Chiralcel AD-H column), Hexane/*i*-PrOH = 95/5, Flow rate: 0.6 mL/min, UV detection at 220 nm, T = 30°C, retention time: t(minor) = 50.4 min, t(major) = 58.8 min; ^{1}H NMR (400 MHz, $CDCl_3$) δ = 5.91-5.82 (m, 1H), 5.25 (dd, *J* = 17.2, 1.2 Hz, 1H), 5.16 (d, *J* = 10.4 Hz, 1H), 4.21 (p, *J* = 6.2 Hz, 1H), 4.06 (d, *J* = 5.7 Hz, 2H), 3.69 (s, 3H), 2.68-2.57 (m, 4H) ppm; ^{13}C NMR (100 MHz, $CDCl_3$) δ = 176.6, 171.3, 134.2, 117.2, 71.9, 70.9, 51.7, 39.2, 39.1 ppm.

(*R*)-5-Methoxy-5-oxo-3-phenylpentanoic acid (9i)

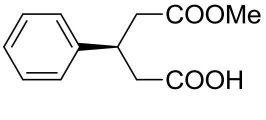

9i

White solid, yield 89%, 77% *ee*; $[\alpha]_D^{25}$ = +7.5 (*c* = 1.0 in $CHCl_3$) (lit.[2] $[\alpha]_D^{20}$ = +8.1 (*c* = 1.62 in $CHCl_3$)); Chiral HPLC (Chiralcel AD-H column), Hexane/*i*-PrOH = 90/10, Flow rate: 0.5 mL/min, UV detection at 220 nm, T = 30°C, retention time: t(minor) = 43.2 min, t(major) = 49.2 min; ^{1}H NMR (400 MHz, $CDCl_3$) δ = 7.31-7.28 (m, 2H), 7.23-7.21 (m, 3H), 3.67-3.61 (m, 1H), 3.58 (s, 3H), 2.80-2.62 (m, 4H) ppm; ^{13}C NMR (100 MHz, $CDCl_3$) δ = 177.7, 172.1, 142.3, 128.7, 127.2, 127.1, 51.6, 40.4, 40.2, 37.9 ppm.

(*R*)-3-(4-Chlorophenyl)-5-methoxy-5-oxopentanoic acid (9j)

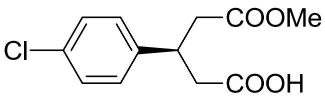

9j

White solid, yield 87%, 78% *ee*; $[\alpha]_D^{25}$ = +3.2 (*c* = 1.0 in $CHCl_3$) (lit.[7] $[\alpha]_D^{25}$ = -8.0 (*c* = 0.88 in $CHCl_3$) for (*S*)-**9j**); Chiral HPLC (Chiralcel OD-H column), Hexane/*i*-PrOH = 92/8, Flow rate: 0.5 mL/min, UV detection at 220 nm, T = 30°C, retention time: t(minor) = 47.7 min, t(major) = 53.3 min; ^{1}H NMR (400 MHz, DMSO) δ = 7.24 (s, 2H), 7.14 (d, J = 8.4 Hz, 2H), 3.63-3.57 (m, 4H), 2.77-2.56 (m, 4H) ppm; ^{13}C NMR (100 MHz, $CDCl_3$) δ = 177.3, 171.8, 140.7, 132.8, 128.8, 128.6, 51.7, 40.2, 40.0, 37.3 ppm.

(*R*)-5-Methoxy-5-oxo-3-(p-tolyl)pentanoic acid (9k)

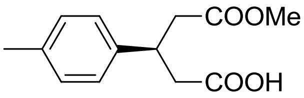

9k

White solid, yield 92%, 79% *ee*; $[\alpha]_D^{25}$ = +5.7 (*c* = 1.0 in $CHCl_3$) (lit.[8] $[\alpha]_D^{20}$ = +5.2 (*c* = 1.0 in $CHCl_3$)); Chiral HPLC (Chiralcel IA-H column), Hexane/*i*-PrOH = 95/5, Flow rate: 1.0 mL/min, UV detection at 220 nm, T = 30°C, retention time: t(minor) = 41.1 min, t(major) = 47.8 min; ^{1}H NMR (400MHz, $CDCl_3$): δ = 7.10 (s, 4H), 3.63–3.56 (m, 4H), 2.78–2.60 (m, 4H), 2.31 (s, 3H) ppm; ^{13}C NMR (100 MHz, $CDCl_3$) δ = 177.6, 172.1, 139.3, 136.6, 129.3, 127.0, 51.6, 40.5, 40.3, 37.5, 21.0 ppm.

(*R*)-5-Methoxy-3-(4-methoxyphenyl)-5-oxopentanoic acid (9l)

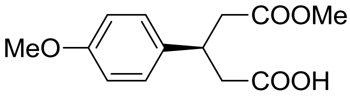

9l

White solid, yield 91%, 80% *ee*; $[\alpha]_D^{25}$ = -4.7 (*c* = 1.0 in EtOH) (lit.[6] $[\alpha]_D^{20}$ = +7.6 (*c* = 0.6 in EtOH) for (*S*)-**9l**); Chiral HPLC (Chiralcel AD-H column), Hexane/*i*-PrOH = 93/7, Flow rate: 0.6 mL/min, UV detection at 220 nm, T = 30°C, retention time: t(minor) = 87.91 min, t(major) = 103.9 min; ^{1}H NMR (400 MHz, $CDCl_3$) δ = 7.13 (d, J = 8.5 Hz, 2H), 6.83 (d, J = 8.5 Hz, 2H), 3.77 (s, 3H), 3.62-3.54 (m, 4H), 2.77-2.58 (m, 4H) ppm; ^{13}C NMR (100 MHz, DMSO) δ = 173.2, 172.2, 158.3, 135.4, 128.8, 114.0, 55.3, 51.6, 40.7, 40.6, 37.7 ppm.

(1*S*,6*R*)-6-(Ethoxycarbonyl)cyclohex-3-enecarboxylic acid (9m)

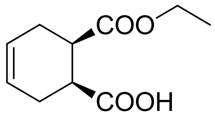

9m

White solid, yield 97%, 93% *ee*; $[\alpha]_D^{25}$ = -1.2 (*c* = 1.0 in $CHCl_3$); Chiral HPLC (Chiralcel IA-H column), Hexane/*i*-PrOH = 94/6, Flow rate: 0.5 mL/min, UV detection at 220 nm, T = 30°C, retention time: t(minor) = 25.7 min, t(major) = 29.5 min; ^{1}H NMR (400 MHz, $CDCl_3$) δ = 11.14 (br, 1H), 5.68 – 5.62 (m, 2H), 4.13 (q, *J* = 7.0 Hz, 2H), 3.03-3.02 (m, 2H), 2.58-2.51 (m, 2H), 2.34 (d, *J* = 18.4 Hz, 2H), 1.21 (t, *J* = 7.1 Hz, 3H); ^{13}C NMR (101 MHz, $CDCl_3$) δ = 179.9, 173.2, 125.2, 125.0, 60.8, 39.7, 39.6, 25.9, 25.5, 14.0 ppm.

(1*S*,6*R*)-6-(Isopropoxycarbonyl)cyclohex-3-enecarboxylic acid (9n)

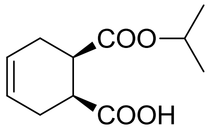

9n

White solid, yield 93%, 89% *ee*; $[\alpha]_D^{25}$ = -7.5 (*c* = 1.0 in $CHCl_3$); Chiral HPLC (Chiralcel IA-H column), Hexane/*i*-PrOH = 94/6, Flow rate: 0.5 mL/min, UV detection at 220 nm, T = 30°C, retention time: t(minor) = 30.4 min, t(major) = 36.1 min; ^{1}H NMR (400 MHz, $CDCl_3$) δ = 11.18 (br, 1H), 5.69-5.62 (m, 2H), 5.00 (dt, *J* = 12.4, 6.2 Hz, 1H), 3.02 (s, 2H), 2.58-2.50 (m, 2H), 2.33 (d, *J* = 16.5 Hz, 2H), 1.19-1.18 (m, 6H) ppm; ^{13}C NMR (100 MHz, $CDCl_3$) δ = 180.2, 172.6, 125.3, 125.0, 68.3, 39.7, 25.9, 25.4, 21.7, 21.6 ppm.

(1*S*,6*R*)-6-((Allyloxy)carbonyl)cyclohex-3-enecarboxylic acid (9o)

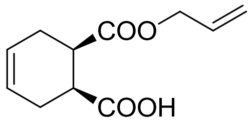

9o

White solid, yield 90%, 85% *ee*; $[\alpha]_D^{25}$ = -0.7 (*c* = 1.0 in $CHCl_3$); Chiral HPLC (Chiralcel IA-H column), Hexane/*i*-PrOH = 94/6, Flow rate: 0.5 mL/min, UV detection at 220 nm, T = 30°C, retention time: t(minor) = 30.3 min, t(major) = 36.2

min; ^{1}H NMR (400 MHz, $CDCl_3$) δ = 11.11 (br, 1H), 5.92-5.82 (m, 1H), 5.69-5.64 (m, 2H), 5.28 (d, *J* = 17.2Hz, 1H), 5.19 (d, *J* = 10.5Hz, 1H), 4.62-4.54 (m, 2H), 3.06 (d, *J* = 5.9 Hz, 2H), 2.62-2.53 (m, 2H), 2.39-2.34 (m, 2H) ppm; ^{13}C NMR (100 MHz, $CDCl_3$) δ = 179.7, 172.9, 132.1, 125.2, 118.1, 65.4, 39.7, 39.6, 25.9, 25.6 ppm.

(1*S*,6*R*)-6-((Benzyloxy)carbonyl)cyclohex-3-enecarboxylic acid (9p)

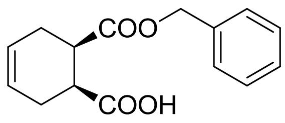

9p

White solid, yield 98%, 86% *ee*; $[\alpha]_D^{25}$ = -1.0 (*c* = 1.0 in $CHCl_3$); Chiral HPLC (Chiralcel AD-H column), Hexane/*i*-PrOH = 95/5, Flow rate: 0.2 mL/min, UV detection at 210 nm, T = 30°C, retention time: t(major) = 69.6 min, t(minor) = 75.6 min; ^{1}H NMR (400 MHz, $CDCl_3$) δ = 10.51 (br, 1H), 7.37-7.29 (m, 5H), 5.69 (s, 2H), 5.19-5.11 (m, 2H), 3.11 (t, *J* = 5.2 Hz, 2H), 2.66-2.57 (m, 2H), 2.42-2.35 (m, 2H) ppm; ^{13}C NMR (100 MHz, $CDCl_3$) δ = 179.8, 173.0, 135.9, 128.5, 128.2, 128.1, 125.2, 125.1, 66.6, 39.7, 39.6, 25.8, 25.6 ppm.

(1*S*,6*R*)-6-((Cinnamyloxy)carbonyl)cyclohex-3-enecarboxylic acid (9q)

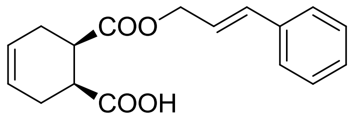

9q

White solid, yield 99%, 90% *ee*; $[\alpha]_D^{25}$ = -3.6 (*c* = 1.0 in $CHCl_3$); Chiral HPLC (Chiralcel AD-H column), Hexane/*i*-PrOH = 94/6, Flow rate: 0.5 mL/min, UV detection at 254 nm, T = 30°C, retention time: t(minor) = 31.1 min, t(major) = 34.6 min; ^{1}H NMR (400 MHz, $CDCl_3$) δ = 7.42-7.29 (m, 5H), 6.64 (d, *J* = 3.2 Hz, 2H), 6.41-6.36 (m, 1H), 5.73 (s, 2H), 4.34-4.33 (m, 2H), 3.14-3.09 (m, 2H), 2.66-2.62 (m, 2H), 2.43-2.39 (m, 2H) ppm; ^{13}C NMR (100 MHz, $CDCl_3$) δ = 177.7, 173.2, 136.7, 131.0, 128.5, 127.6, 126.4, 125.1, 123.1, 65.3, 63.4, 39.7, 39.6, 25.8, 25.6 ppm.

6. Catalytic asymmetric synthesis of (*S*)-GABOB by 7i

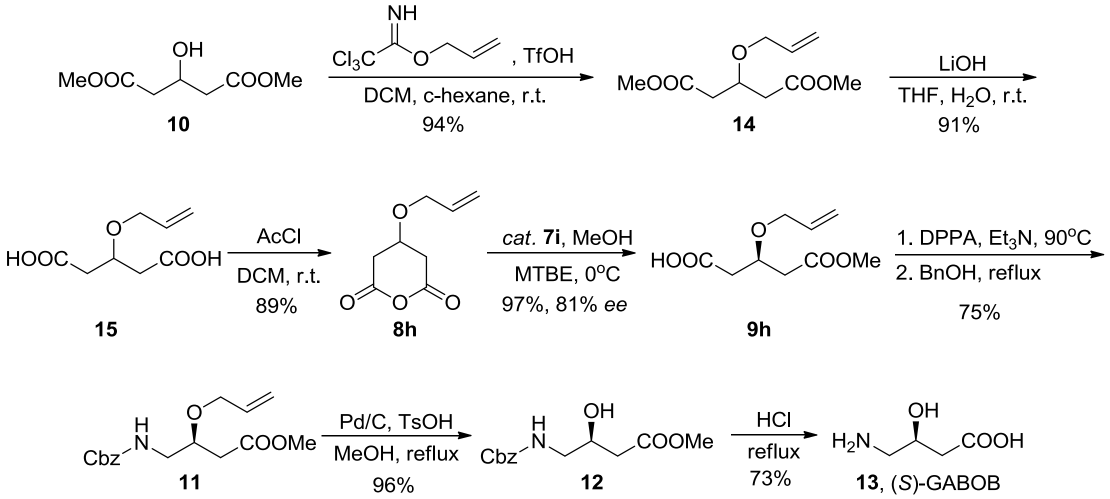

Dimethyl 3-(allyloxy)pentanedioate (14)

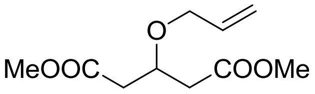

14

Under N_2 atmosphere, to a stirred solution of dimethyl 3-hydroxypentanedioate (40 g, 0.23 mol) and allyl 2,2,2-trichloroacetimidate (91.9 g, 0.46 mol) in 1000 mL of 4:1 c-hexane/CH_2Cl_2 at 0 °C was added TfOH (4 mL, 46 mmol). After addition, the reaction mixture was stirred for 24 h at room temperature and then filtered. The filtrate was washed with $NaHCO_3$ (2 × 200 mL), dried over Na_2SO_4 and concentrated under reduced pressure to give yellow oil. The crude product was purified by flash chromatography using PE/EA 9:1 to give product **14** (45.6 g, 93%) as colourless oil; ^{1}H NMR (400 MHz, $CDCl_3$) δ = 5.90-5.81 (m, 1H), 5.24 (dd, *J* = 17.2, 1.3 Hz, 1H), 5.15 (d, *J* = 10.5 Hz, 1H), 4.21 (p, *J* = 6.3 Hz, 1H), 4.04 (d, *J* = 5.7 Hz, 2H), 3.68 (s, 6H), 2.60 (qd, *J* = 15.5, 6.3 Hz, 4H) ppm; ^{13}C NMR (100 MHz, $CDCl_3$) δ = 170.8, 134.0, 116.5, 71.7, 70.4, 51.1, 38.8 ppm; HRMS (ESI$^+$) calcd for $C_{10}H_{16}O_5$ $[M+Na]^+$ = 239.0895, found: 239.0892.

3-(Allyloxy)pentanedioic acid (15)

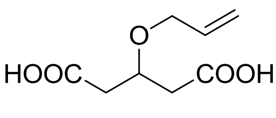

15

To a stirred solution of **14** (45 g, 0.21 mol) in 750 mL of 4:1 THF/H_2O was added LiOH·H_2O (22.03 g, 0.53 mol) at 0 °C. The reaction mixture was stirred at room temperature for 24 h before the THF was evaporated. The aqueous layer was washed with EtOAc (2 × 100 mL), acidified with 2 N HCl, and then extracted with EtOAc (3 × 200 mL). The organic phase was washed with brine (300 mL), dried over Na_2SO_4 and concentrated to afford **15** (35.6 g, 91%) as yellow oil; ^{1}H NMR (400 MHz, $CDCl_3$) δ = 9.23 (br, 2H), 5.93 – 5.83 (m, 1H), 5.27 (dd, *J* = 17.2, 1.5 Hz, 1H), 5.19-5.16 (m, 1H), 4.22 (p, *J* = 6.2 Hz, 1H), 4.08 (d, *J* = 5.7 Hz, 2H), 2.69 (qd, *J* = 15.8, 6.2 Hz, 4H) ppm; ^{13}C NMR (100 MHz, $CDCl_3$) δ = 176.9, 134.1, 117.5, 71.7, 71.0, 39.1 ppm; HRMS (ESI$^+$) calcd for $C_8H_{13}O_5$ $[M+H]^+$ = 189.0763, found: 189.0757.

3-(Allyloxy)glutaric anhydride (8h)

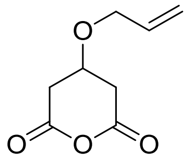

8h

To a stirred suspension of **15** (33 g, 0.18 mol) in 500 mL of CH_2Cl_2 at 0 °C was added AcCl (124 mL, 1.8 mmol). The reaction mixture was stirred at room temperature for 12 h and concentrated. The crude product was dissolved in 500 mL of CH_2Cl_2, washed with $NaHCO_3$ (3 × 300 mL) and brine (300mL), dried over Na_2SO_4 and concentrated to afford **8h** (26.5 g, 89%) as a yellow oil; ^{1}H NMR (400 MHz, $CDCl_3$) δ = 5.89-5.79 (m, 1H), 5.30-5.21 (m, 2H), 4.08-4.03 (m, 3H), 3.09 (dd, *J* = 16.6, 3.5 Hz, 2H), 2.75 (d, J = 18.9 Hz, 2H) ppm; ^{13}C NMR (100 MHz, $CDCl_3$) δ = 165.0, 133.4, 118.1, 69.9, 67.0, 35.7 ppm; HRMS (ESI$^+$) calcd for $C_8H_{10}O_4$ $[M+H]^+$ = 171.0657, found: 171.0652.

(*R*)-3-(Allyloxy)-5-methoxy-5-oxopentanoic acid (9h)

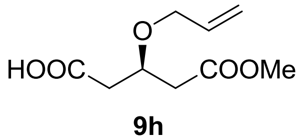

9h

MeOH (10.1 mL, 250 mmol) was added dropwise at 0 °C under nitrogen to a stirred solution of *meso*-cyclic anhydride **8h** (4.26 g, 25 mmol) and **7i** (1.80 g, 2.5 mmol) in

MTBE (2 L). The reaction was monitored by using thin-layer chromatography. After 96 h, anhydride consumption was complete, the solvent was evaporated under reduced pressure and the residue was dissolved in CH_2Cl_2 (300 mL). The solution was washed with saturated Na_2CO_3 (3 × 150 mL) and the combined aqueous layers were acidified with excess 2 N HCl, followed by extraction with EtOAc (3 × 300 mL). The combined organic layers were dried over Na_2SO_4 and concentrated to afford momoester **9h** (4.9 g, yield 97%, 81% *ee*) as light yellow oil; $[\alpha]_D^{25}$ = -3.5 (*c* = 1.0 in $CHCl_3$); Chiral HPLC (Chiralcel AD-H column), Hexane/*i*-PrOH = 95/5, Flow rate: 0.6 mL/min, UV detection at 220 nm, *T* = 30 °C, retention time: t(minor) = 50.6 min, t(major) = 56.1 min; Chiral HPLC (Chiralcel AD-H column), Hexane/i-PrOH=95/5, Flow rate: 0.6 mL/min, UV detection at 220 nm, t(minor) = 50.6 min, t(major) = 56.1 min; ^{1}H NMR (400 MHz, $CDCl_3$) δ = 5.91-5.82 (m, 1H), 5.25 (dd, *J* = 17.2, 1.2 Hz, 1H), 5.16 (d, *J* = 10.4 Hz, 1H), 4.21 (p, *J* = 6.2 Hz, 1H), 4.06 (d, *J* = 5.7 Hz, 2H), 3.69 (s, 3H), 2.68-2.57 (m, 4H) ppm; ^{13}C NMR (100 MHz, $CDCl_3$) δ = 176.6, 171.3, 134.2, 117.2, 71.9, 70.9, 51.7, 39.2, 39.1 ppm; HRMS (ESI^+) calcd for $C_9H_{14}O_5$ $[M+Na]^+$ = 225.0739, found: 225.0733.

(*S*)-Methyl 3-(allyloxy)-4-(((benzyloxy)carbonyl)amino)butanoate (11)

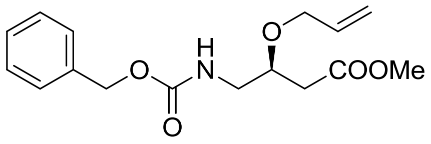

11

Diphenylphosphoryl azide (6.5 mL, 30 mmol) was added to a dry toluene solution (100 mL) of **9h** (5 g, 25 mmol) and triethylamine (4.2 mL, 30 mmol) at room temperature. The reaction mixture was stirred for 30 min and then it was slowly warmed to 90 ^{o}C. When the evolution of nitrogen ceased (30 min), benzyl alcohol (3.1 mL, 30 mmol) was added, and the mixture was heated at reflux overnight. The reaction mixture was washed with $NaNO_2$ (1% aq., 2 × 250 mL), $NaHCO_3$ (2 × 250 mL), H_2O (250 mL), dried over Na2SO4 and concentrated to afford brown oil. The crude product was purified by flash chromatography using PE/EA 10:1 to give product **11** (5.7 g, 75%) as light yellow oil; $[\alpha]_D^{25}$ = +5.4 (*c* = 1.0 in $CHCl_3$); ^{1}H NMR

(400 MHz, $CDCl_3$) δ = 7.35-7.30 (m, 5H), 5.89-5.80 (m, 1H), 5.23 (d, *J* = 17.2 Hz, 1H), 5.15 (d, *J* = 10.2 Hz, 2H), 5.09 (s, 2H), 4.02 (d, *J* = 4.9 Hz, 2H), 3.92-3.90 (m, 1H), 3.67 (s, 3H), 3.42-3.37 (m, 1H), 3.33-3.27 (m, 1H), 2.59-2.45 (m, 2H) ppm; ^{13}C NMR (100 MHz, $CDCl_3$) δ = 171.4, 156.6, 136.5, 134.4, 128.5, 128.2, 128.1, 117.4, 74.4, 70.8, 66.8, 51.8, 43.8, 37.4 ppm; HRMS (ESI^+) calcd for $C_{16}H_{21}NO_5$ $[M+Na]^+$ = 330.1317, found: 330.1306.

(*S*)-Methyl 4-(((benzyloxy)carbonyl)amino)-3-hydroxybutanoate (12)

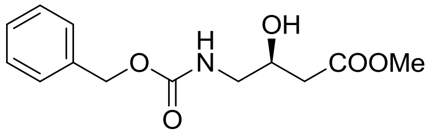

12

To a solution of **11** (0.9 g, 3 mmol) and Pd/C (450 mg) in MeOH (30 mL) was added 4-methylbenzenesulfonic acid (57 mg, 0.3 mmol). The reaction mixture was heated at reflux for 3 h and filtered. The filtrate was concentrated to afford yellow oil. The crude product was purified by flash chromatography using PE/EA 5:1 to give product **12** (0.75 g, 96%) as light yellow oil; $[\alpha]_D^{25}$ = +2.5 (*c* = 1.0 in $CHCl_3$); ^1H NMR (400 MHz, $CDCl_3$) δ = 7.34 (s, 5H), 5.29 (br, 1H), 5.10 (s, 2H), 4.12 (br, 1H), 3.70 (s, 3H), 3.44 (s, 1H), 3.38 (s, 1H), 3.22-3.15 (m, 1H), 2.54-2.44 (m, 2H) ppm; ^{13}C NMR (100 MHz, $CDCl_3$) δ = 172.9, 157.0, 136.4, 128.6, 128.3, 128.2, 67.5, 67.0, 52.0, 45.9, 38.4 ppm.

(*S*)-GABOB (13)

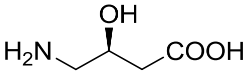

13, (*S*)-GABOB

(*S*)-methyl 4-(((benzyloxy)carbonyl)amino)-3-hydroxybutanoate (**12**, 5 g, 19 mmol) was added to 16 N HCl (60 mL) and then the reaction mixture was heated at reflux overnight. The mixture was concentrated under reduced pressure and the residue was crystallized from EtOH to give white solid, then dissolved in H_2O (5 mL) and added NaOH (0.54 g). The stirred suspention was added H_2O until the solid disappeared, then EtOH (20 mL) was added and filtered, oven dry the filter cake to afford (S)-GABOB (**13**, 1.63 g, 73%) as white solid; 96% *ee*, determined from optical

rotation, $[\alpha]_D^{25}$ = +19.7 (*c* = 1.4 in H_2O) (lit.[9] $[\alpha]_D^{25}$ = +20.56 (*c* = 1.41 in H_2O)), recrystallization from 78% EtOH further increased the optical purity to 99% *ee*, $[\alpha]_D^{25}$ = +20.4 (*c* = 1.5 in H_2O); ^{1}H NMR (400 MHz, D_2O) δ = 4.21-4.15 (m, 1H), 3.15 (dd, *J* = 13.1, 3.0 Hz, 1H), 2.93 (dd, *J* = 13.1, 9.5 Hz, 1H), 2.41 (d, *J* = 6.5 Hz, 2H) ppm; ^{13}C NMR (100 MHz, D_2O) δ = 178.6, 65.5, 44.1, 42.3 ppm; HRMS (ESI$^+$) calcd for $C_4H_9NO_3$ $[M+H]^+$ = 120.0661, found: 120.0654.

7. References

[1] M. N. Patil, R. G. Gonnade, N. N. Joshi, *Tetrahedron* **2010**, *66*, 5036-5041.

[2] A. Peschiulli, Y. Gun’ko, S. J. Connon, *J. Org. Chem.* **2008**, *73*, 2454-2457.

[3] Y. -G. Chen, S. -K. Tian, L. Deng, *J. Am. Chem. Soc.* **2000**, *122*, 9542-9543

[4] S. -X. Wang, F. -E. Chen, *Adv. Synth. Catal.* **2009**, *351*, 547-552.

[5] S. E. Park, E. H. Nam, H. B. Jang, J. S. Oh, S. Some, Y. S. Lee, C. E. Song, *Adv. Synth. Catal.* **2010**, *352*, 2211-2217.

[6] A. Fryszkowska, M. Komar, D. Koszelewskia, R, Ostaszewski, *Tetrahedron: Asymmetry* **2005**,*16*, 2475-2485.

[7] H. -J. Yang, F. -J. Xiong, J. Li, F. -E., Chen, *Chinese. Chem. Lett.* **2013**, *24*, 553-558.

[8] L. -J. Yan, H. F. Wang, W. X. Chen, Y. Tao, K. J. Jin, F. E. Chen, *ChemCatChem.* **2016**, *8*, 2249 – 2253.

[9] T. Ivšić, I. Dokli, A. Rimac, Z. Hameršak, *Eur. J. Org. Chem.* **2014**, 631-638.

8. Copies of NMR spectra and chiral HPLC spectra

_N_-((1_R_,2_R_)-2-(Dimethylamino)-1-(4-nitrophenyl)-3-(trityloxy)propyl)-4-(trifluoromethyl)benzamide (7a)

^{1}H NMR

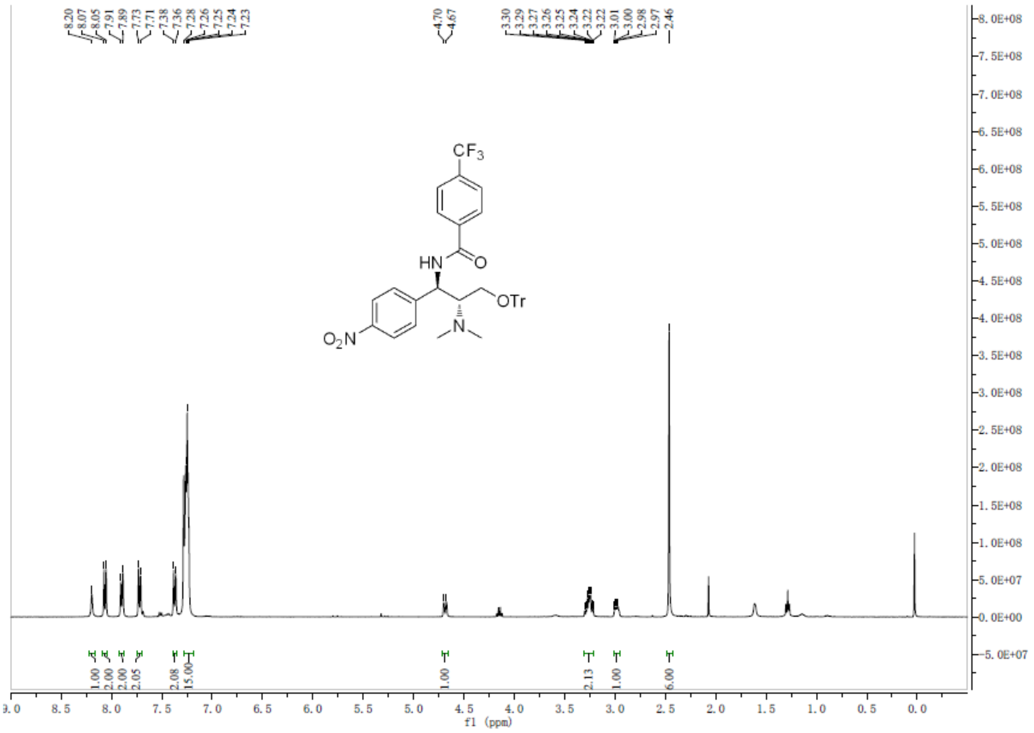

^{13}C NMR

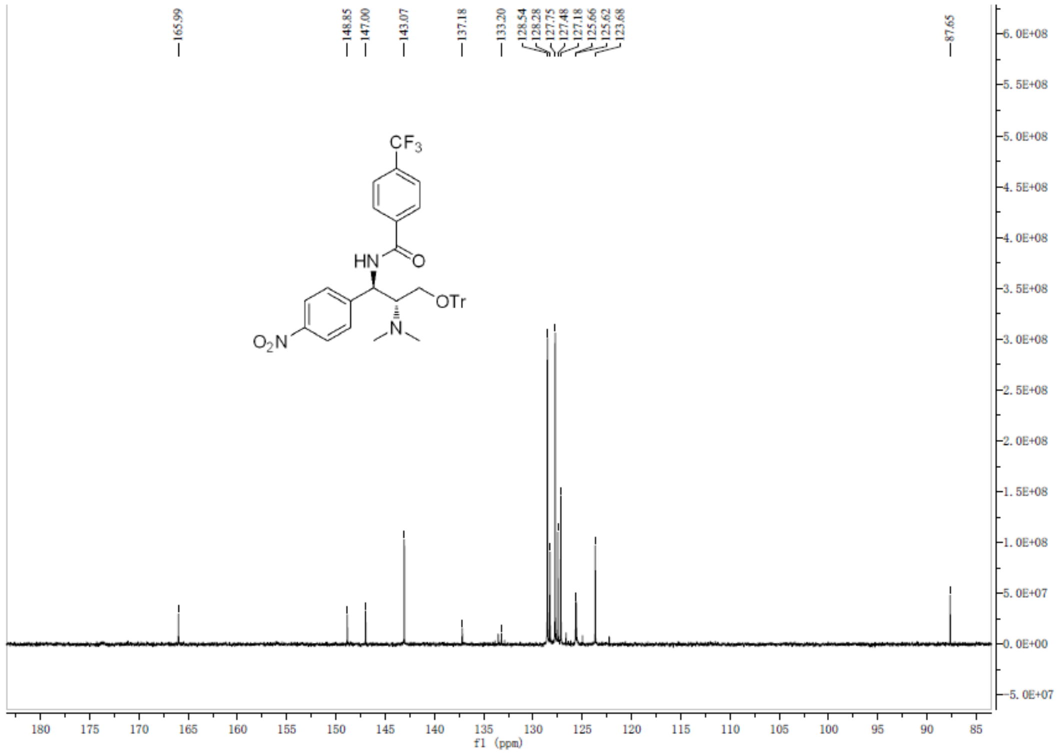

HRMS

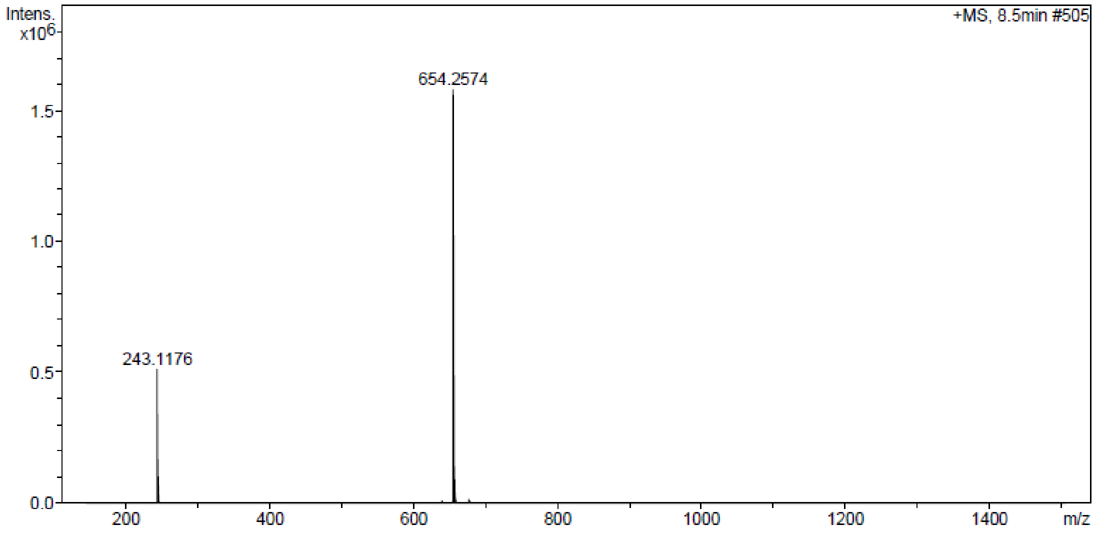

N-((1R,2R)-2-(Dimethylamino)-1-(4-nitrophenyl)-3-(trityloxy)propyl)-4-nitro-benzamide (7b)

^{1}H NMR

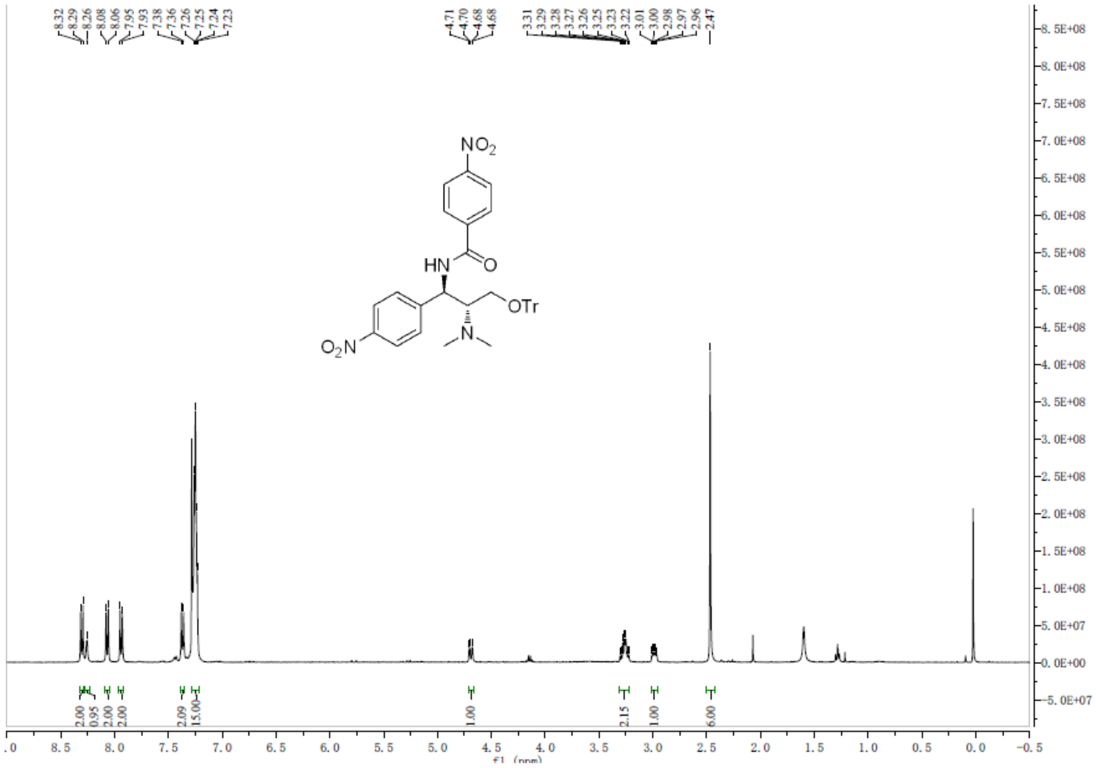

^{13}C NMR

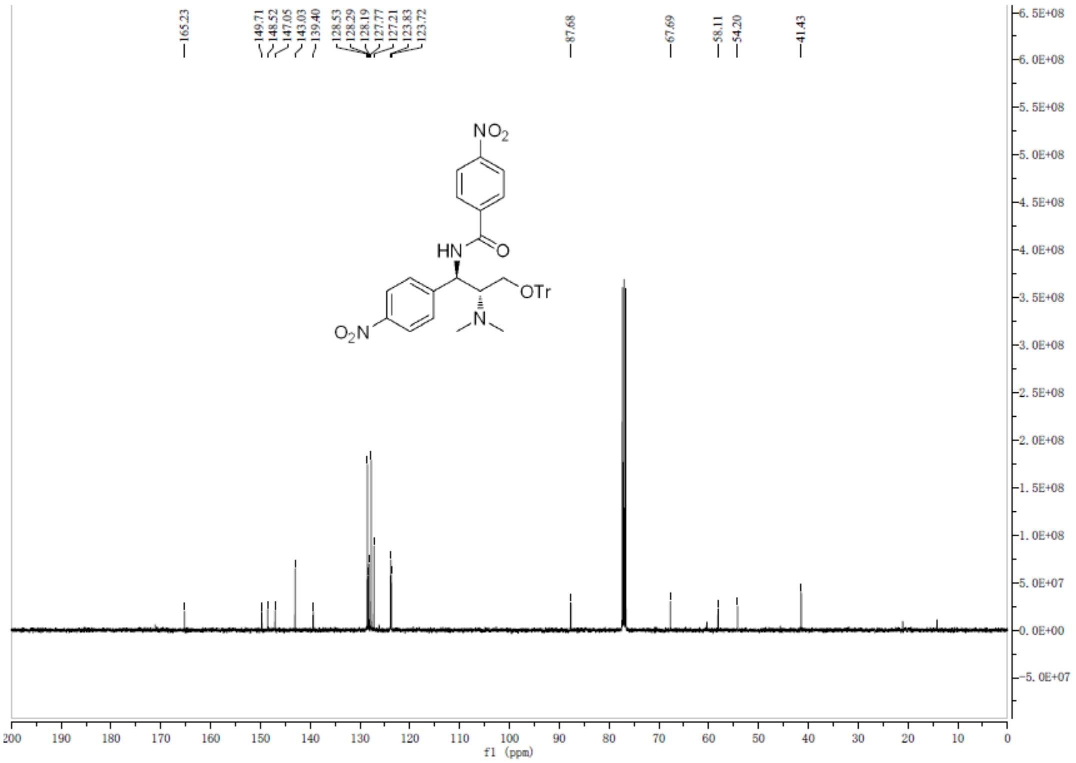
165.23
149.71
148.52
147.05
143.03
139.40
128.53
128.29
128.19
127.77
127.21
123.83
123.72
87.68
67.69
58.11
54.20
41.43
NO2
HN
O
OTr
N
O2N
f1 (ppm)

HRMS

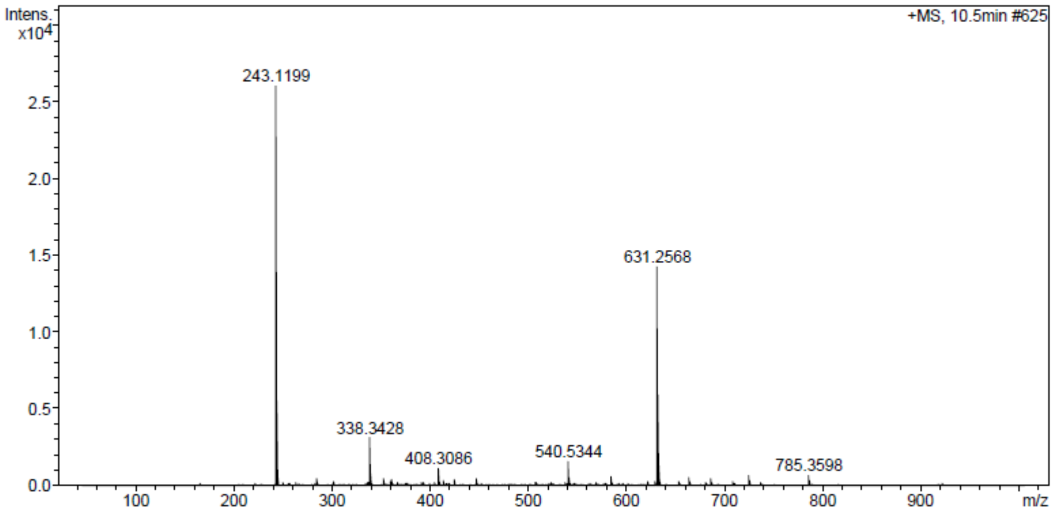
Intens.
x10^4
+MS, 10.5min #625
243.1199
631.2568
338.3428
408.3086
540.5344
785.3598
m/z

N-((1R,2R)-2-(Dimethylamino)-1-(4-nitrophenyl)-3-(trityloxy)propyl)-4-fluoro-benzamide (7c)

^{1}H NMR

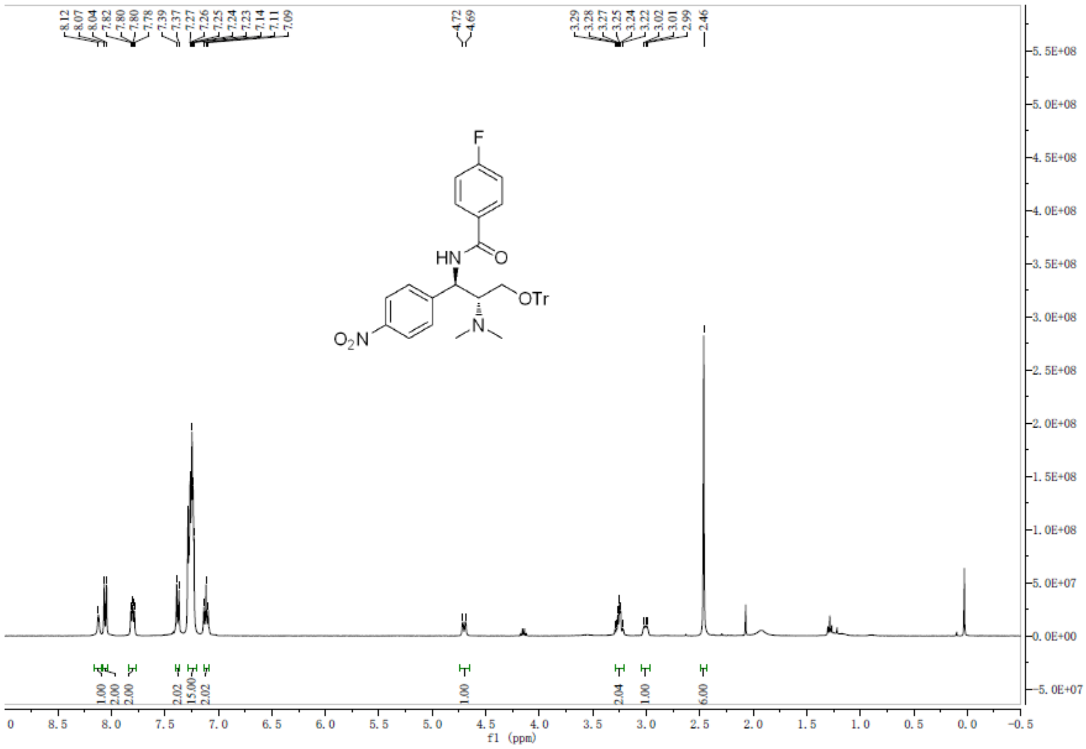

^{13}C NMR

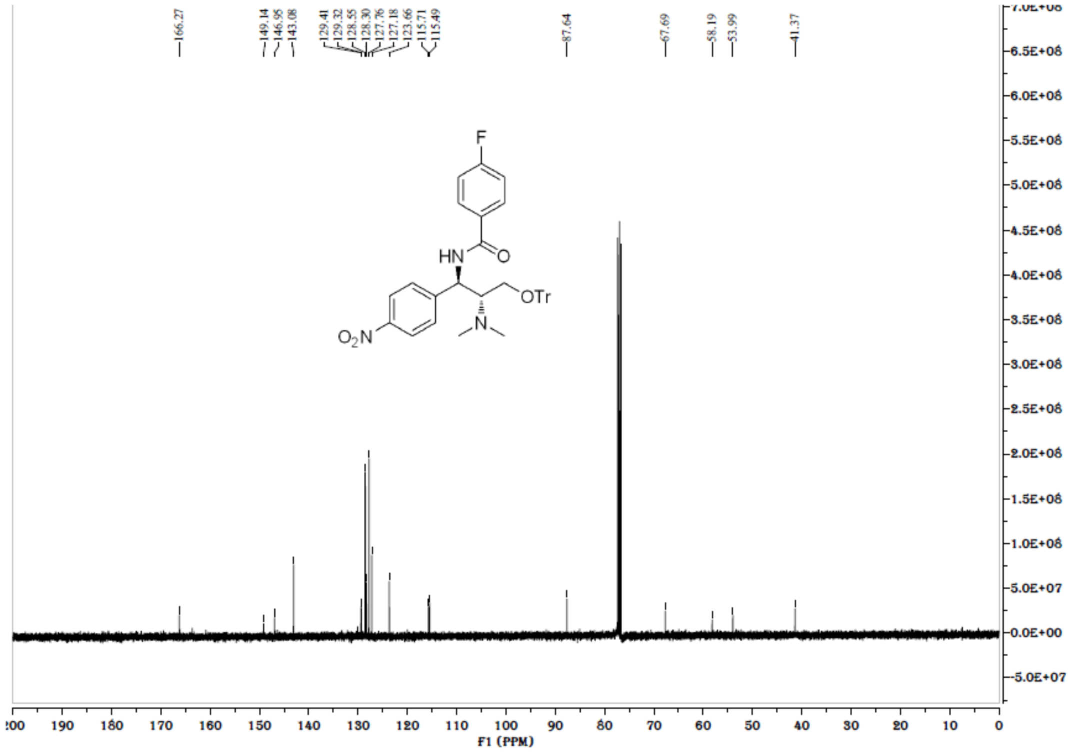

HRMS

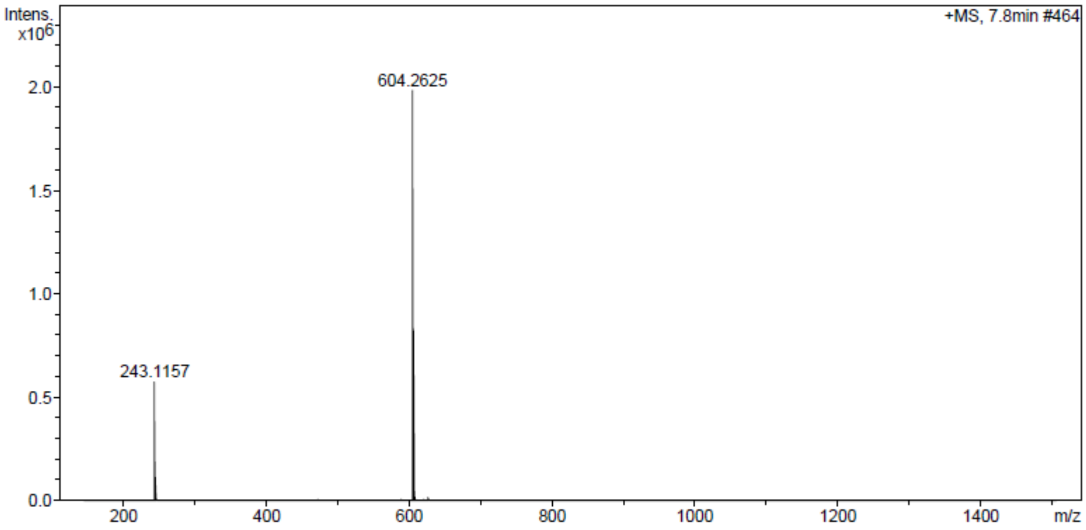

4-Chloro-*N*-((1*R*,2*R*)-2-(dimethylamino)-1-(4-nitrophenyl)-3-(trityloxy)propyl)-benzamide (7d)

^{1}H NMR

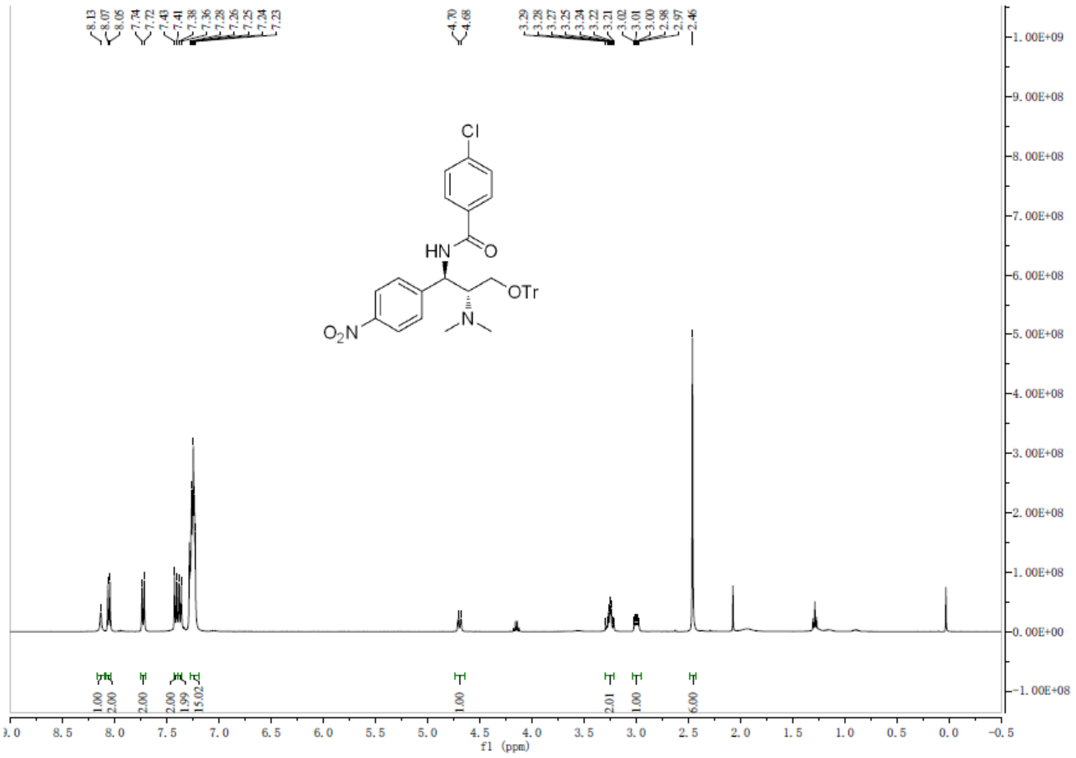

^{13}C NMR

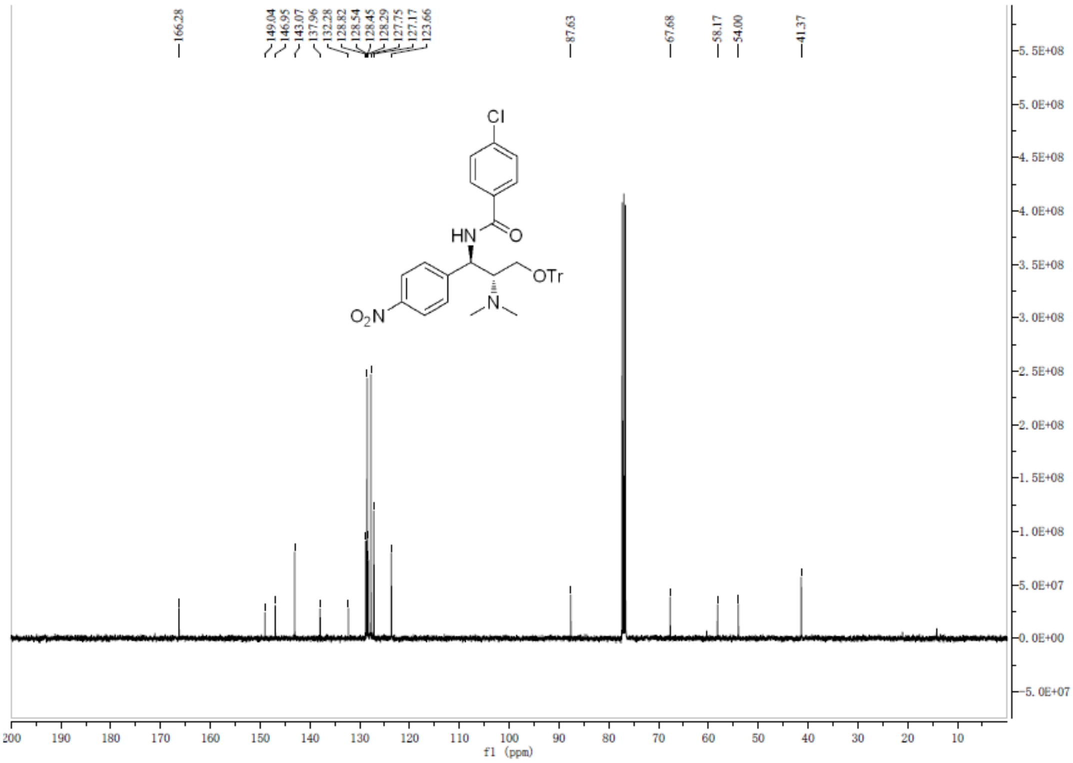
166.28
149.04
146.95
143.07
137.96
132.28
128.82
128.54
128.45
128.29
127.75
127.17
123.66
87.63
67.68
58.17
54.00
41.37
Cl
HN
O
OTr
N
O2N
f1 (ppm)

HRMS

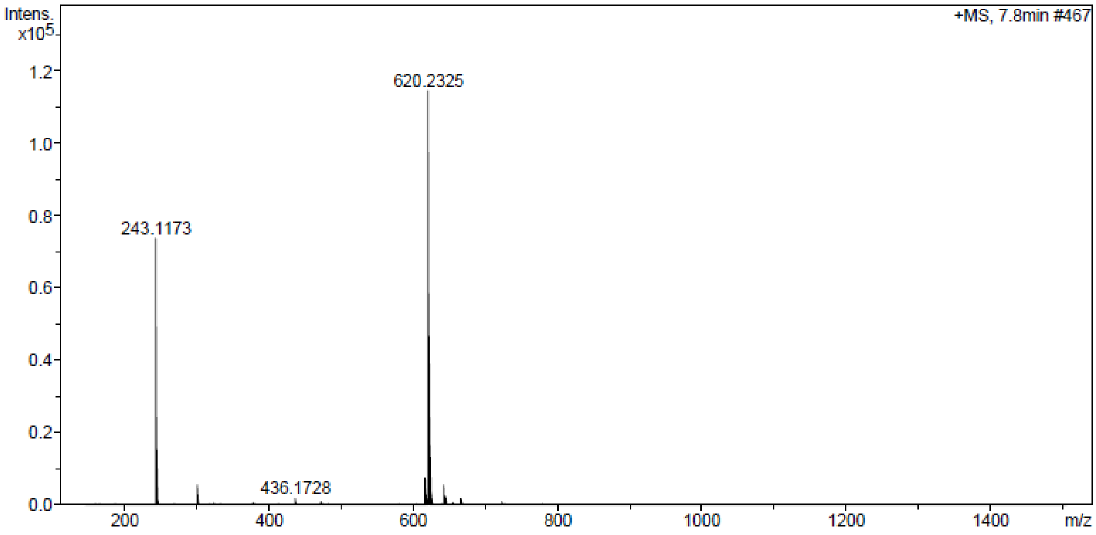
Intens.
x10^5
+MS, 7.8min #467
620.2325
243.1173
436.1728
m/z

4-Bromo-*N*-((1*R*,2*R*)-2-(dimethylamino)-1-(4-nitrophenyl)-3-(trityloxy)propyl)-benzamide (7e)

^{1}H NMR

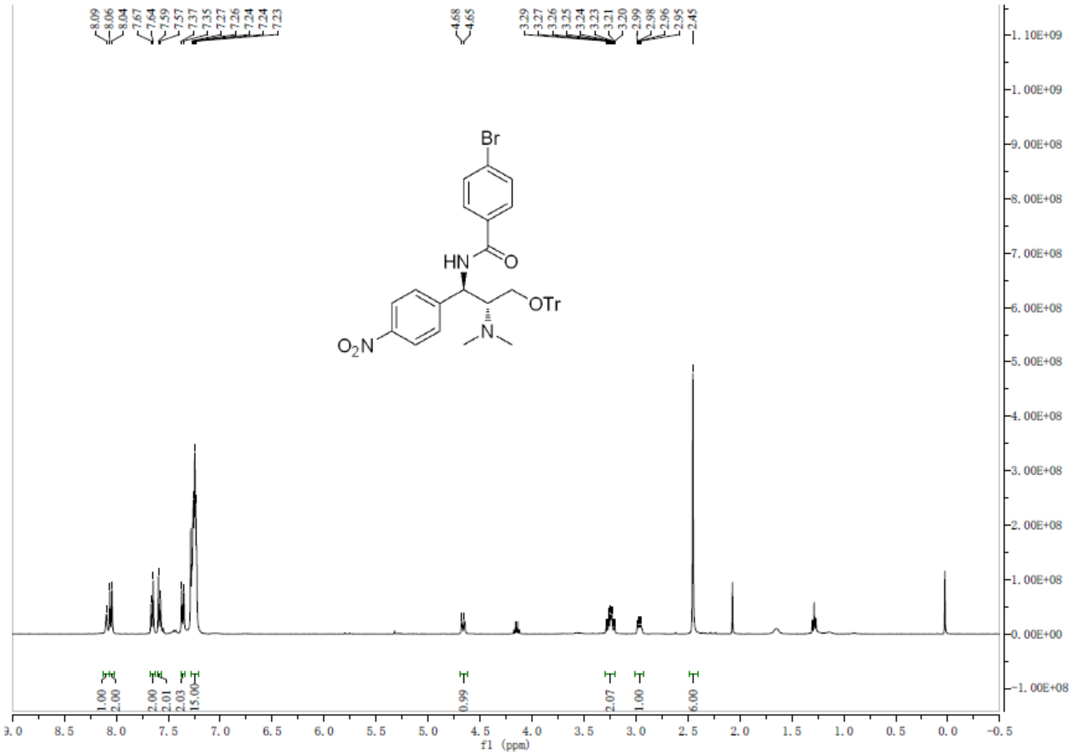

^{13}C NMR

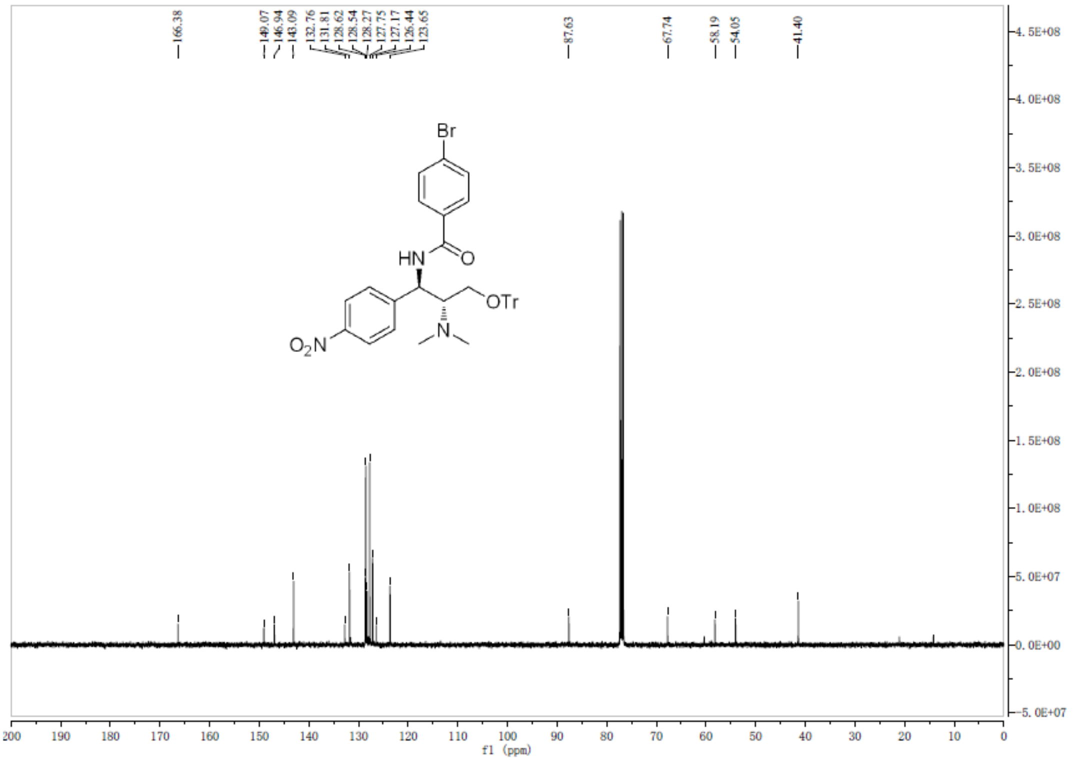

HRMS

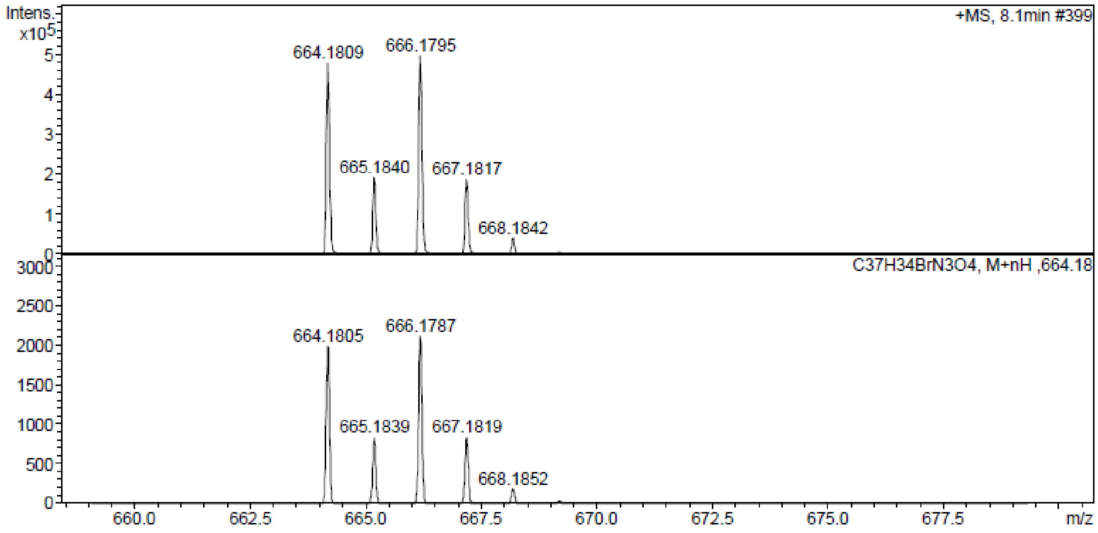

***N*-((1*R*,2*R*)-2-(Dimethylamino)-1-(4-nitrophenyl)-3-(trityloxy)propyl)benzamide (7f)**

^{1}H NMR

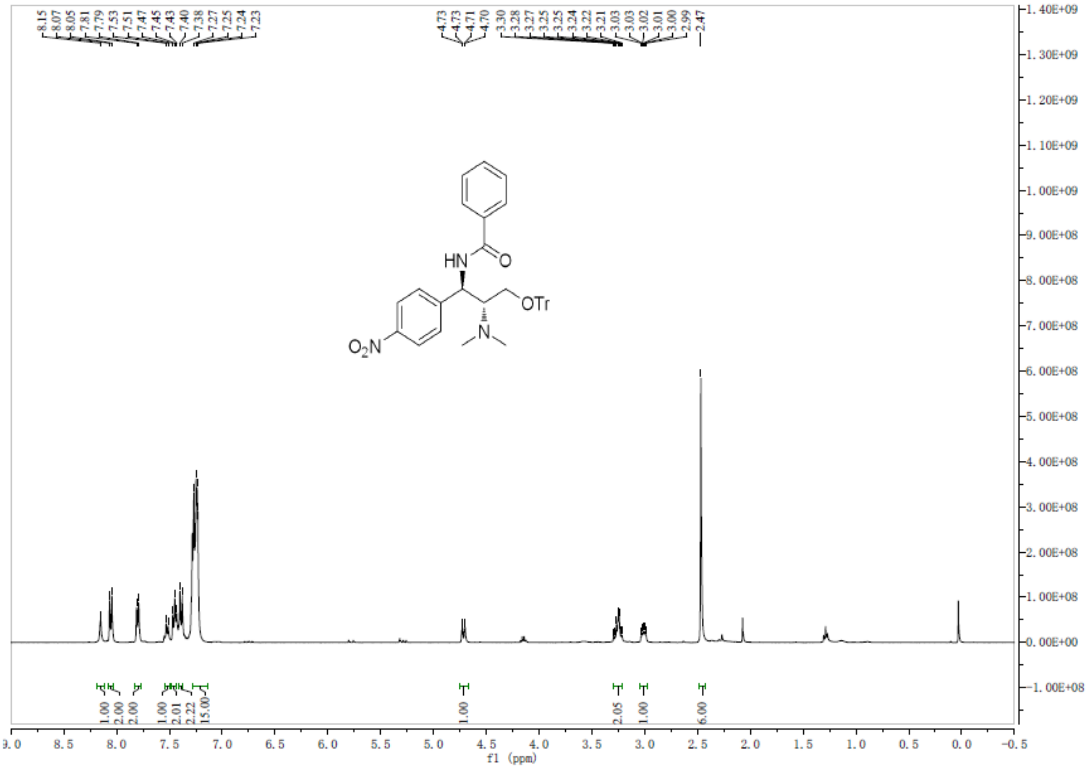

^{13}C NMR

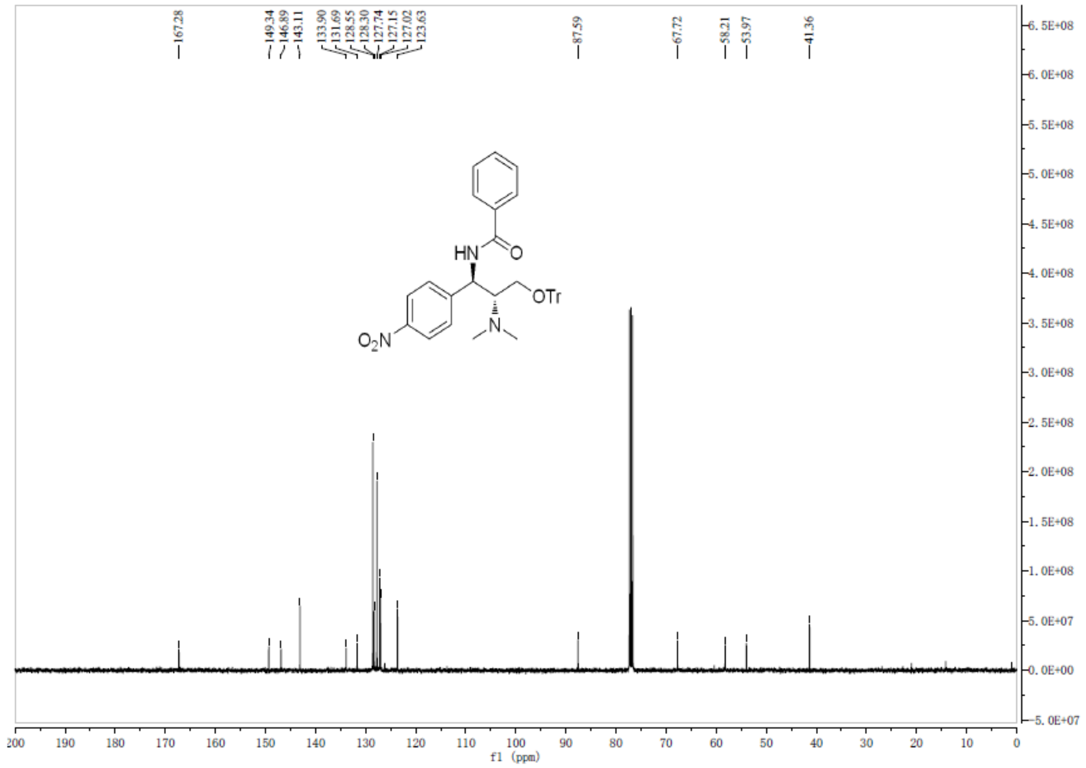

167.28
149.34
146.89
143.11
133.90
131.69
128.55
128.30
127.74
127.15
127.02
123.63
87.59
67.72
58.21
53.97
41.36
HN
O
OTr
O_2N
N
f1 (ppm)

HRMS

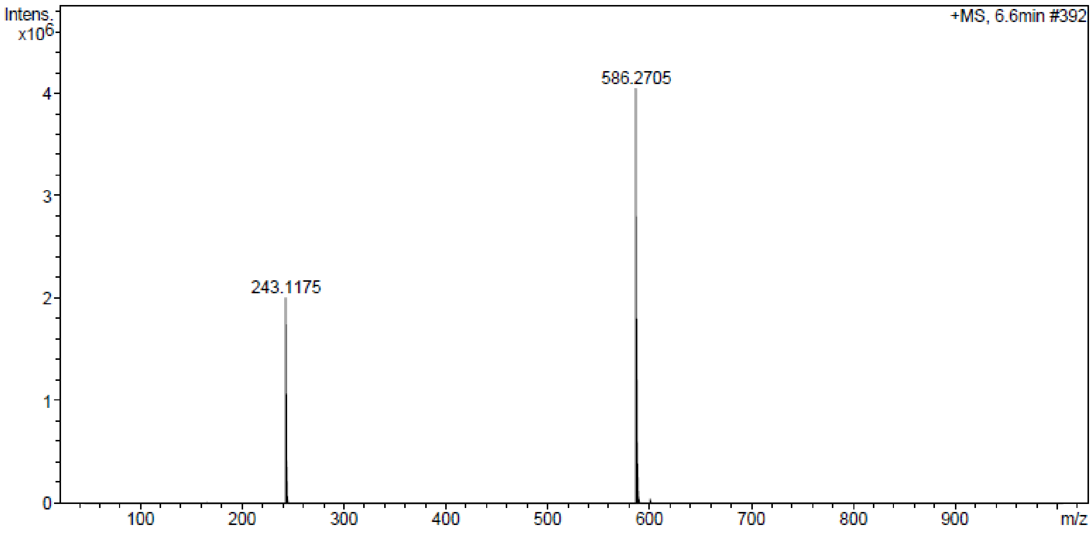

Intens.
x10^6
+MS, 6.6min #392
586.2705
243.1175
m/z

***N*-((1*R*,2*R*)-2-(Dimethylamino)-1-(4-nitrophenyl)-3-(trityloxy)propyl)-4-methyl-benzamide (7g)**

^{1}H NMR

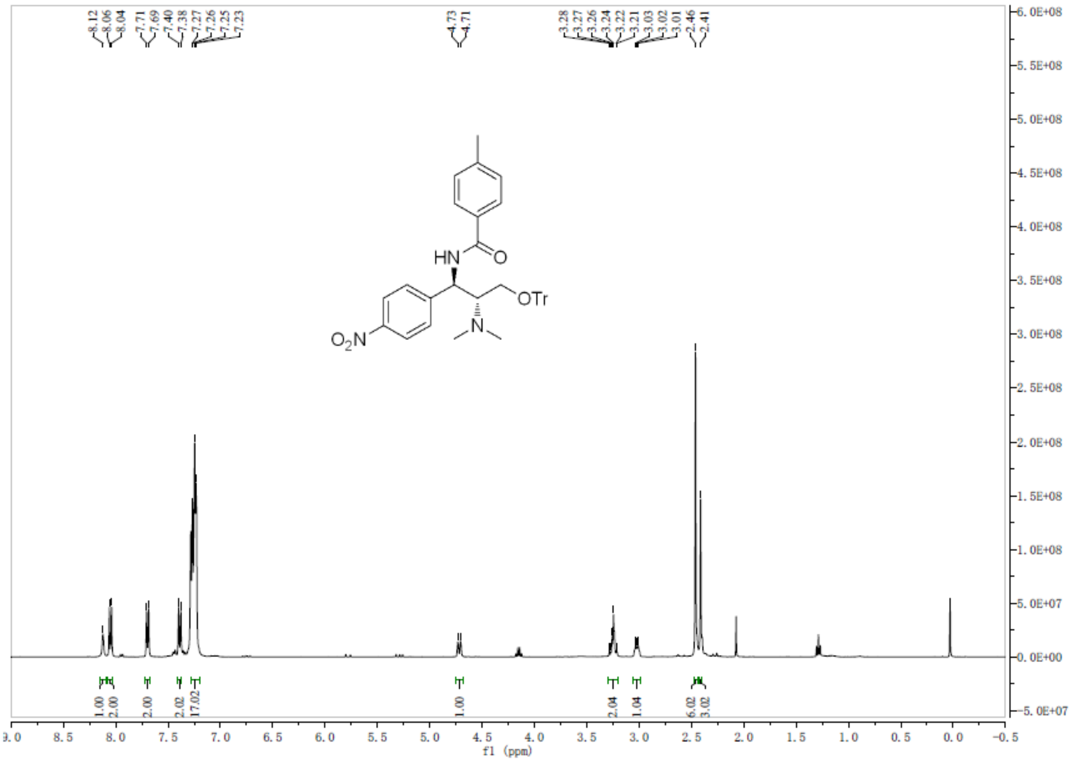

^{13}C NMR

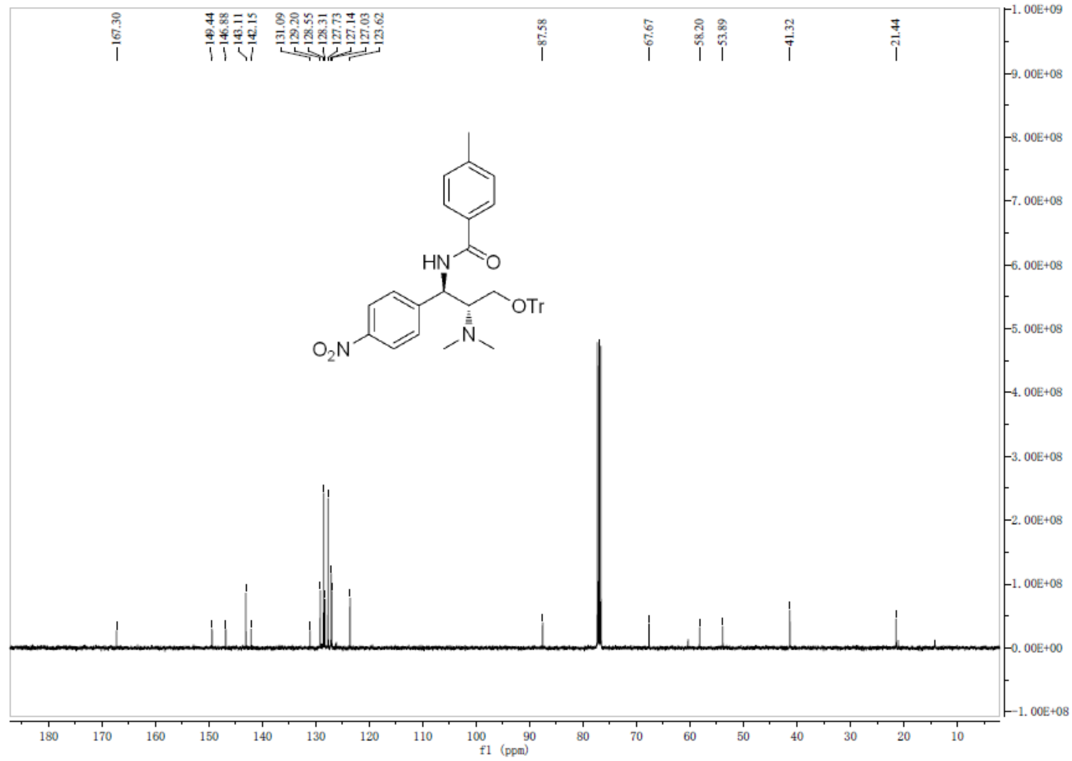

HRMS

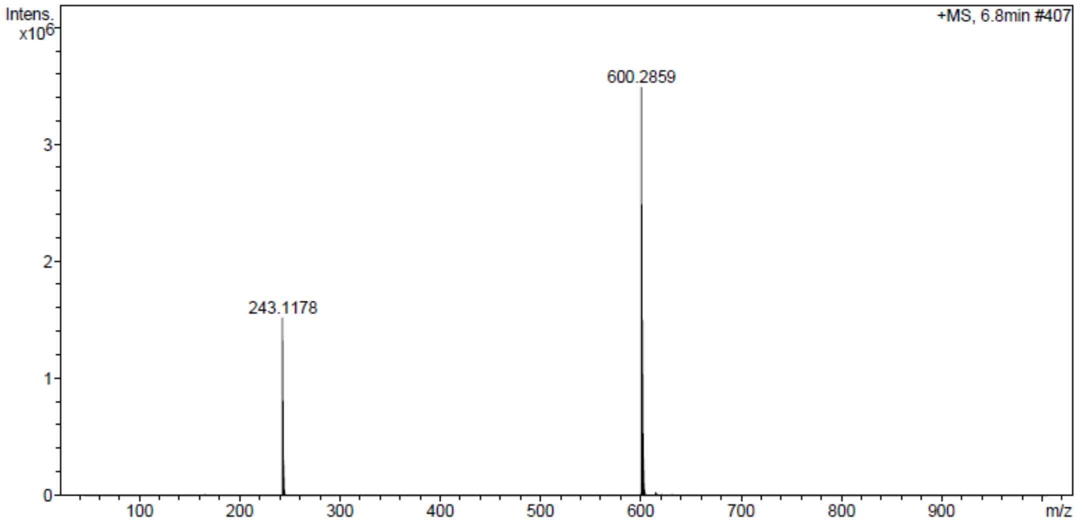

N-((1R,2R)-2-(Dimethylamino)-1-(4-nitrophenyl)-3-(trityloxy)propyl)-4-methoxy-benzamide (7h)

^{1}H NMR

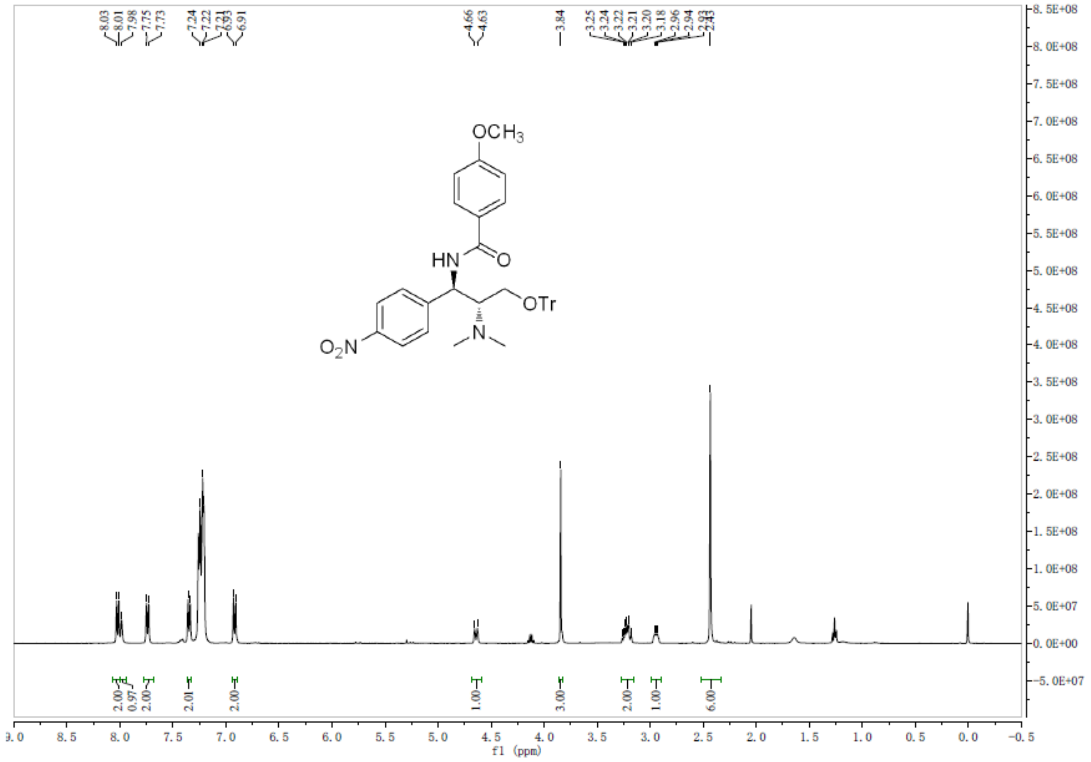

^{13}C NMR

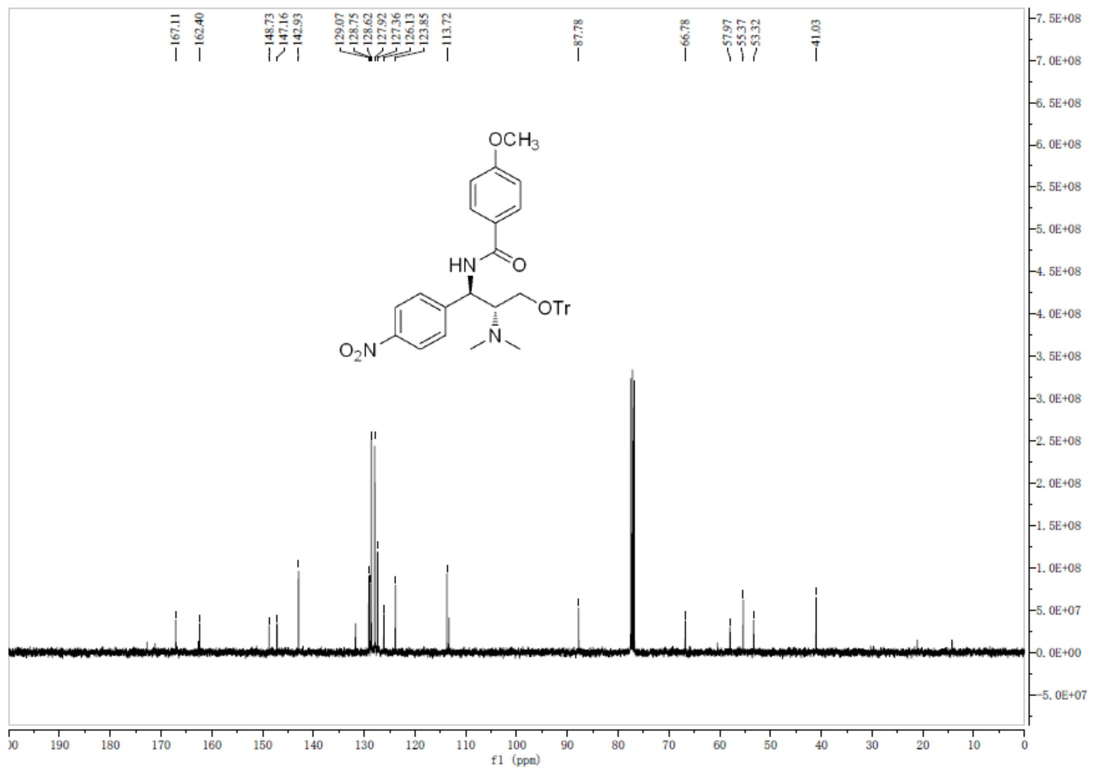

167.11
162.40
148.73
147.16
142.93
129.07
128.75
128.62
127.92
127.36
126.13
123.85
113.72
87.78
66.78
57.97
55.37
53.32
41.03
OCH3
HN
O
OTr
N
O2N
f1 (ppm)

HRMS

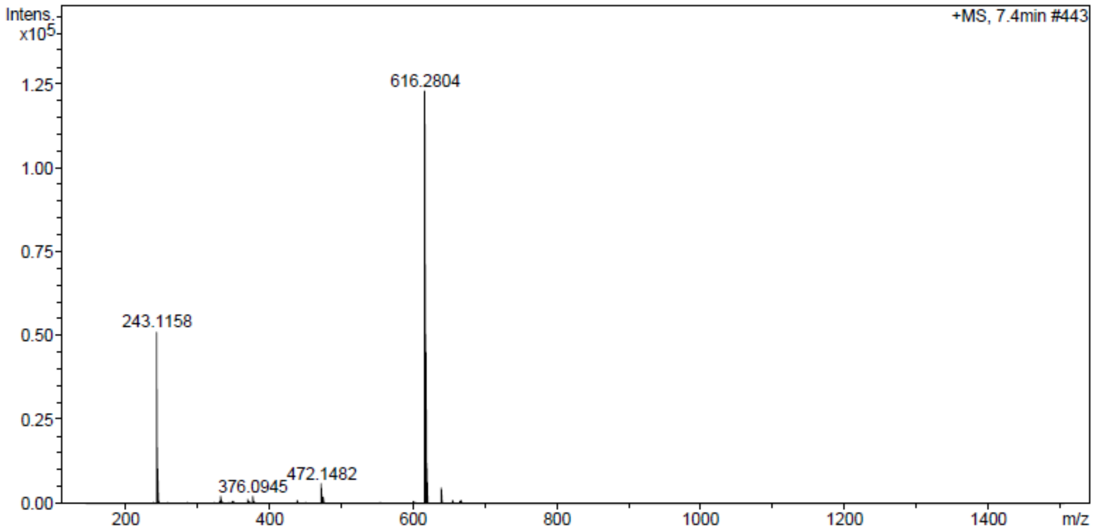

Intens.
x10^5
+MS, 7.4min #443
616.2804
243.1158
376.0945
472.1482
m/z

N-((1R,2R)-2-(Dimethylamino)-1-(4-nitrophenyl)-3-(trityloxy)propyl)-3,5-bis-(trifluoromethyl)benzamide (7i)

^{1}H NMR

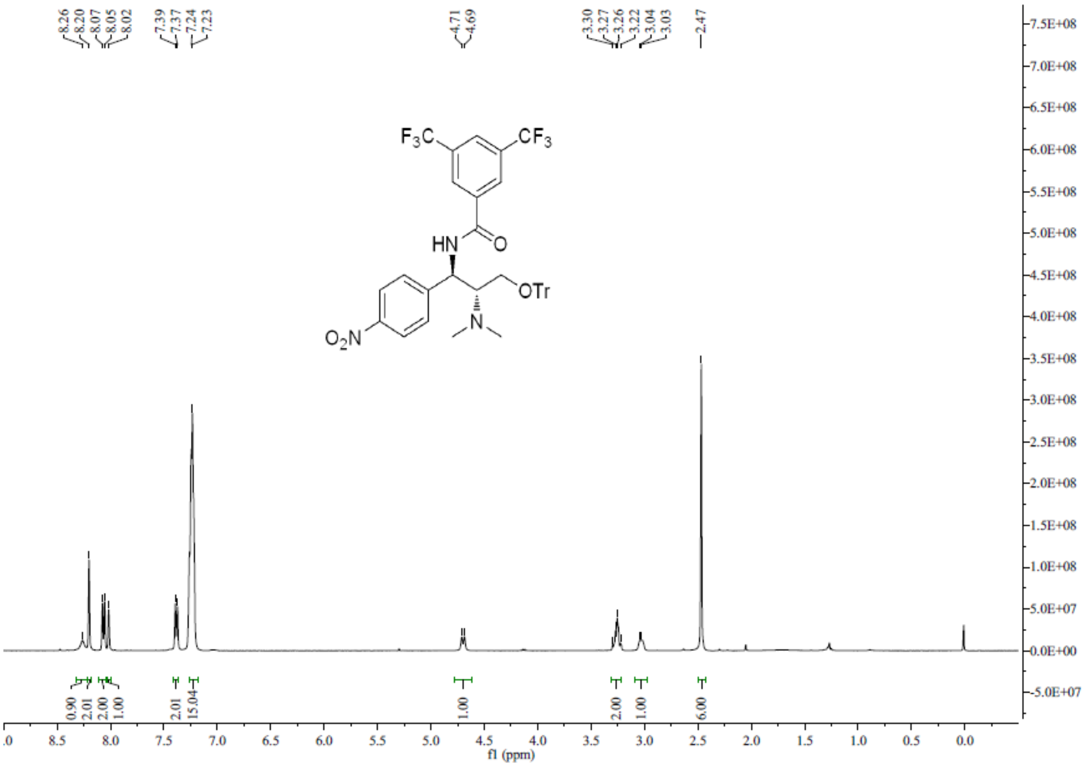

^{13}C NMR

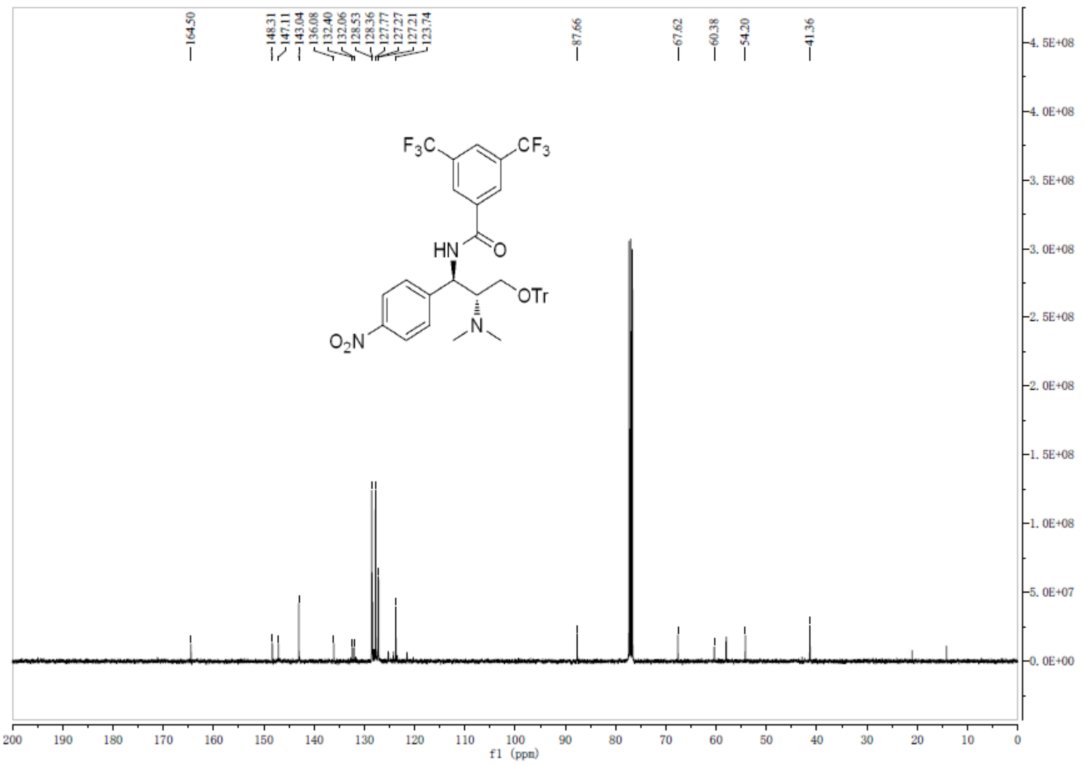

HRMS

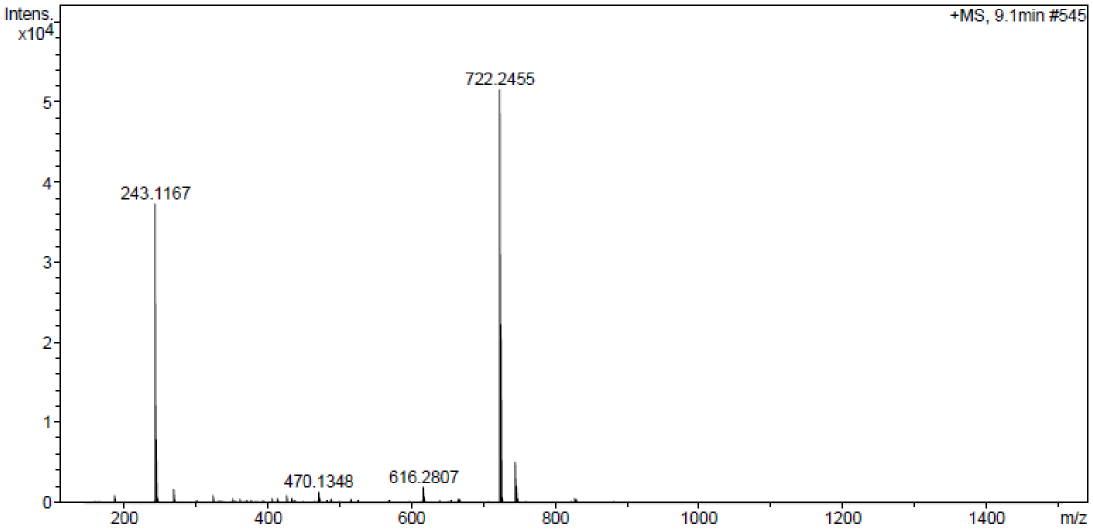

N-((1R,2R)-1-(4-Nitrophenyl)-2-(pyrrolidin-1-yl)-3-(trityloxy)propyl)-3,5-bis-(trifluoromethyl)benzamide (7j)

^{1}H NMR

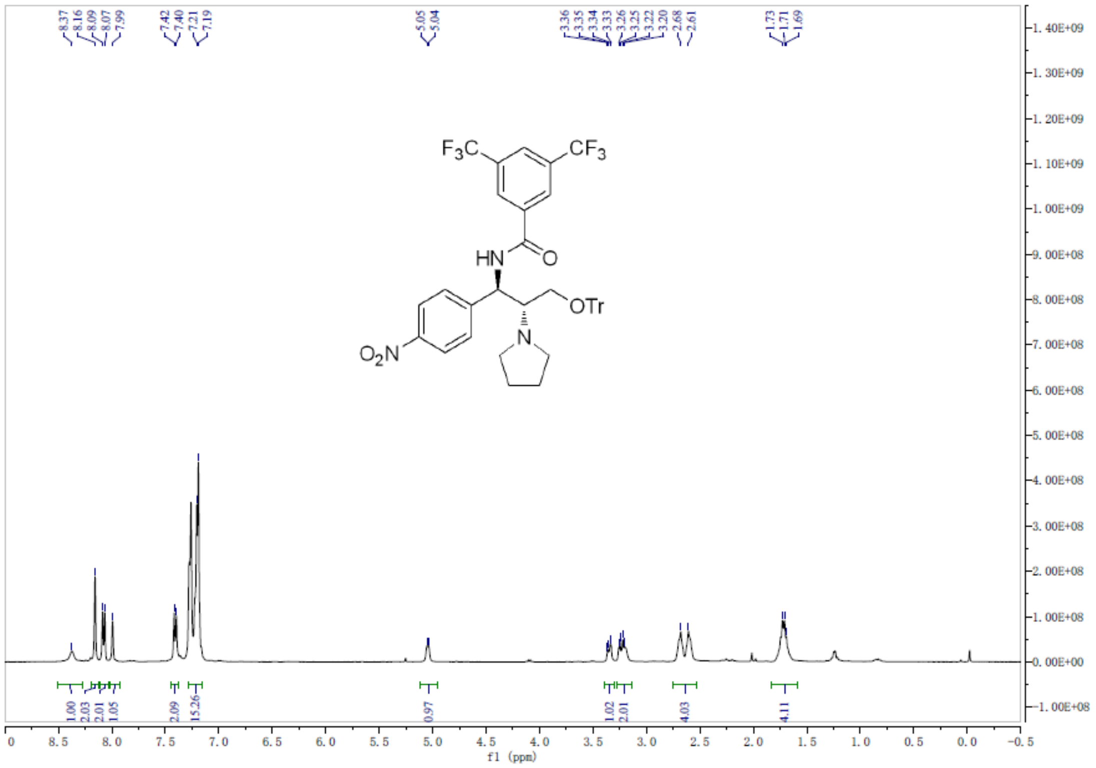

^{13}C NMR

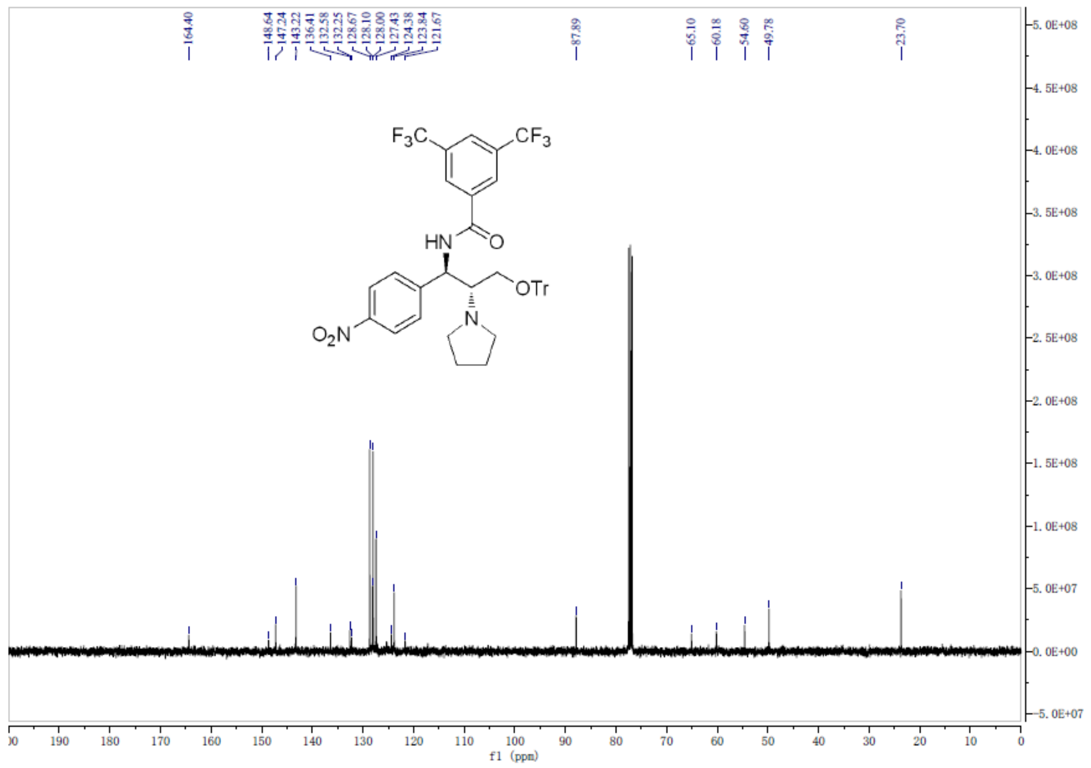
164.40
148.64
147.24
143.22
136.41
132.58
132.25
128.67
128.10
128.00
127.43
124.38
123.84
121.67
87.89
65.10
60.18
54.60
49.78
23.70
F3C
CF3
HN
O
OTr
N
O2N
f1 (ppm)

HRMS

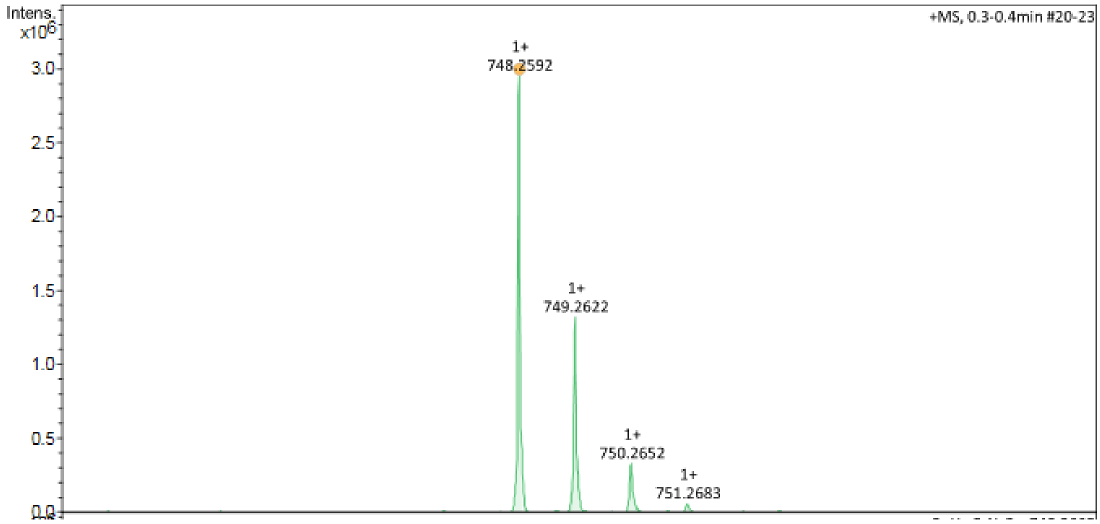
Intens.
x10^6
+MS, 0.3-0.4min #20-23
1+
748.2592
1+
749.2622
1+
750.2652
1+
751.2683

***N*-((1*R*,2*R*)-1-(4-Nitrophenyl)-2-(piperidin-1-yl)-3-(trityloxy)propyl)-3,5-bis-(trifluoromethyl)benzamide (7k)**

^{1}H NMR

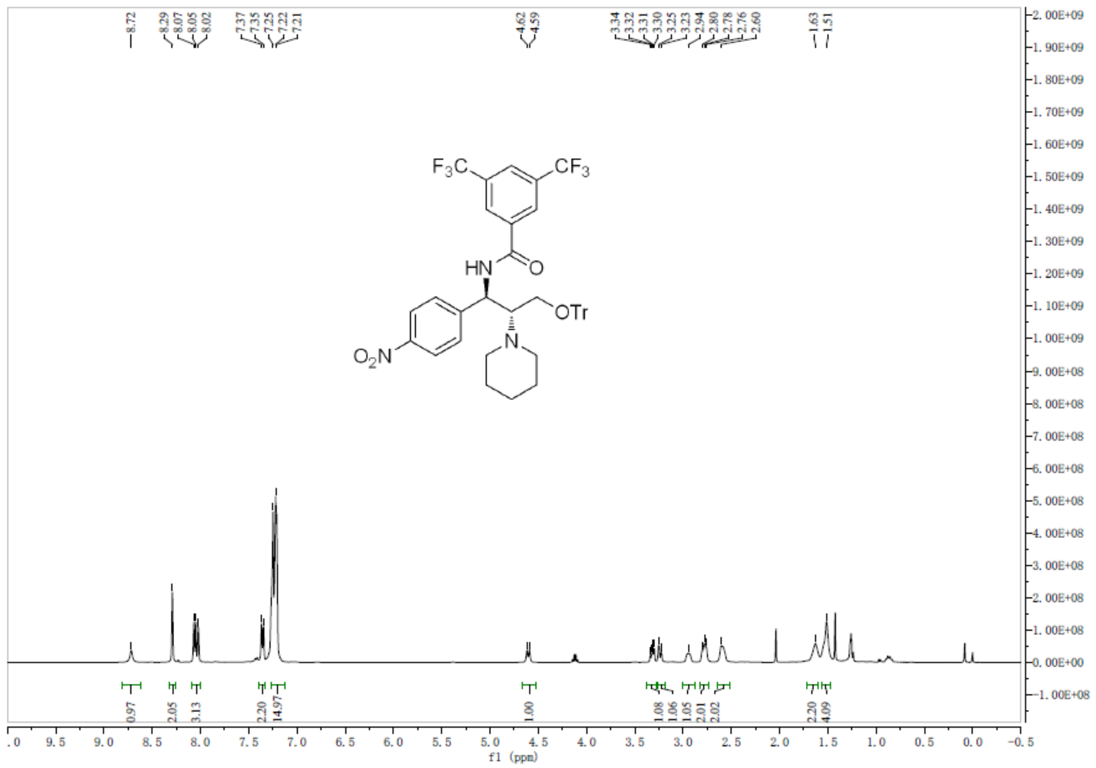

^{13}C NMR

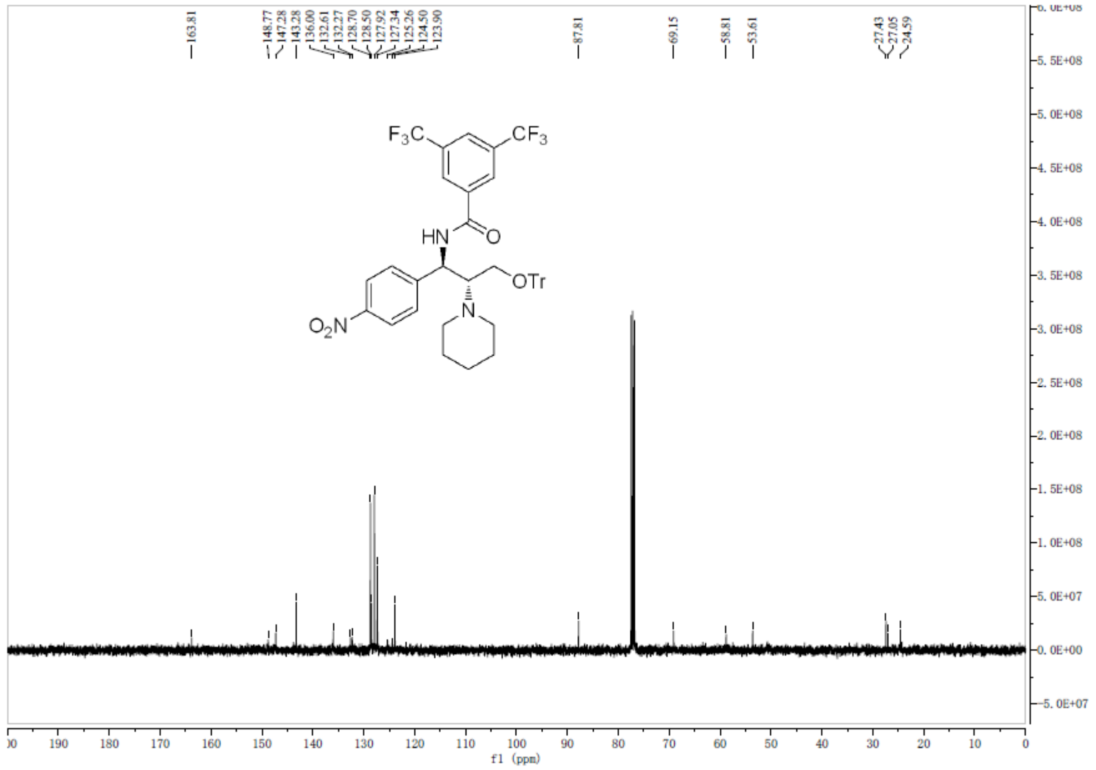

HRMS

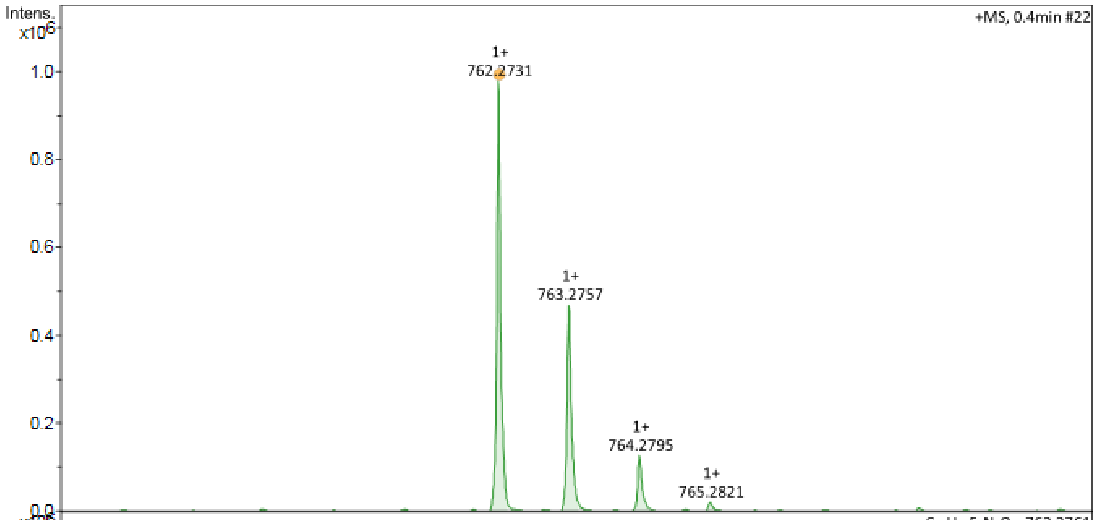

***N*-((1*R*,2*R*)-2-Morpholino-1-(4-nitrophenyl)-3-(trityloxy)propyl)-3,5-bis(trifluoro-methyl)benzamide (7l)**

^{1}H NMR

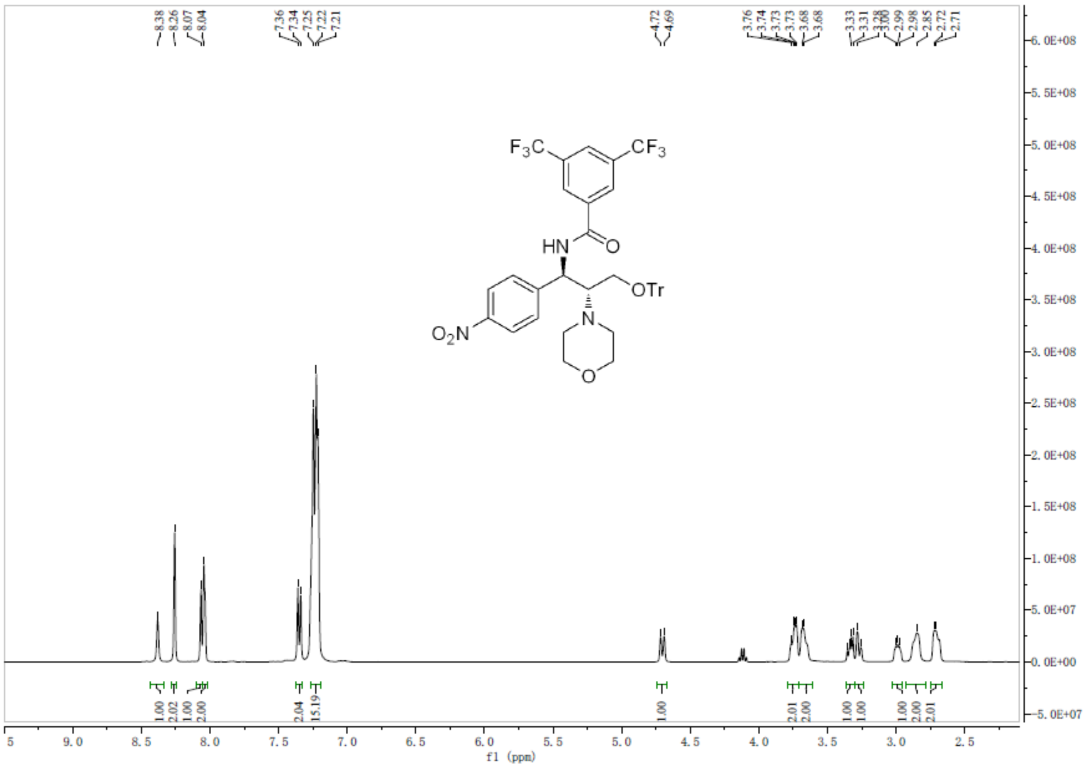

^{13}C NMR

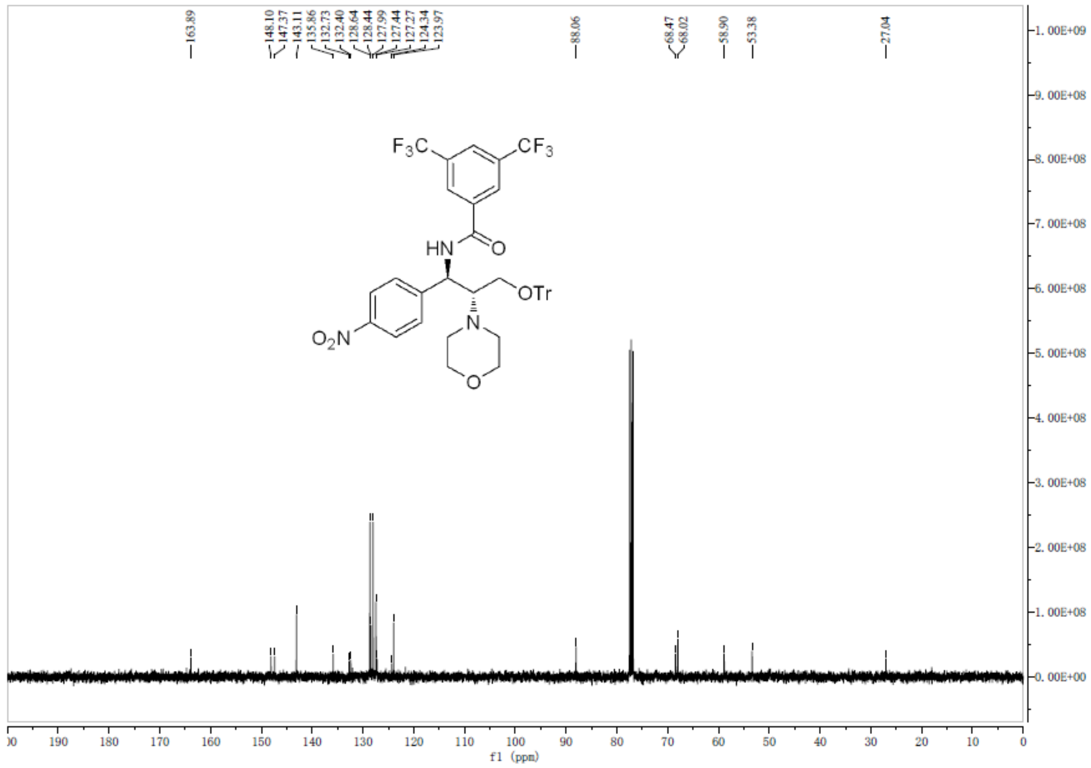

163.89
148.10
147.37
143.11
135.86
132.73
132.40
128.64
128.44
127.99
127.44
127.27
124.34
123.97
88.06
68.47
68.02
58.90
53.38
27.04
F3C
CF3
HN
O
OTr
N
O2N
O
f1 (ppm)

HRMS

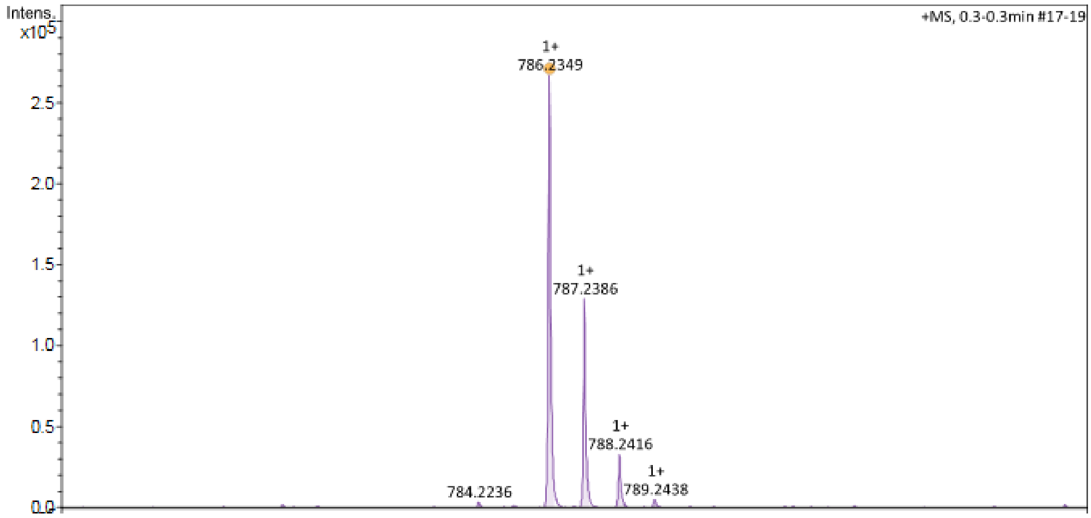

Intens.
x10^5
+MS, 0.3-0.3min #17-19
1+
786.2349
1+
787.2386
1+
788.2416
1+
789.2438
784.2236

N-((1R,2R)-3-((tert-Butyldimethylsilyl)oxy)-2-(dimethylamino)-1-(4-nitrophenyl)-propyl)-3,5-bis(trifluoromethyl)benzamide (7m)

^{1}H NMR

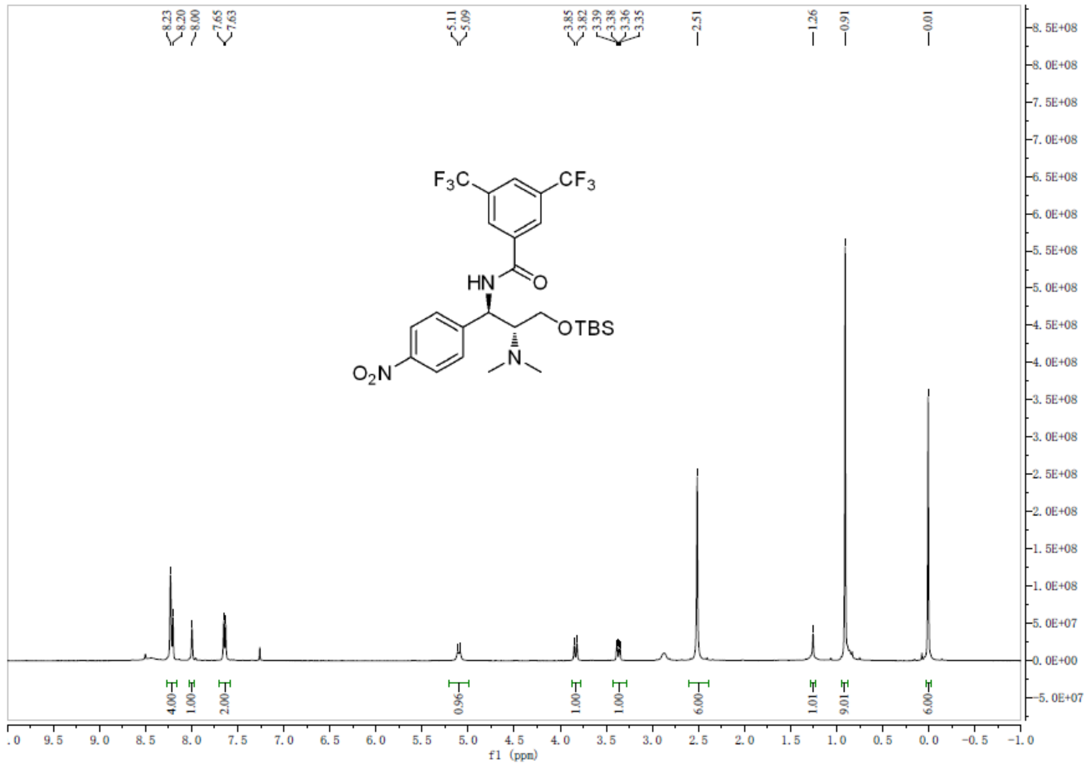

^{13}C NMR

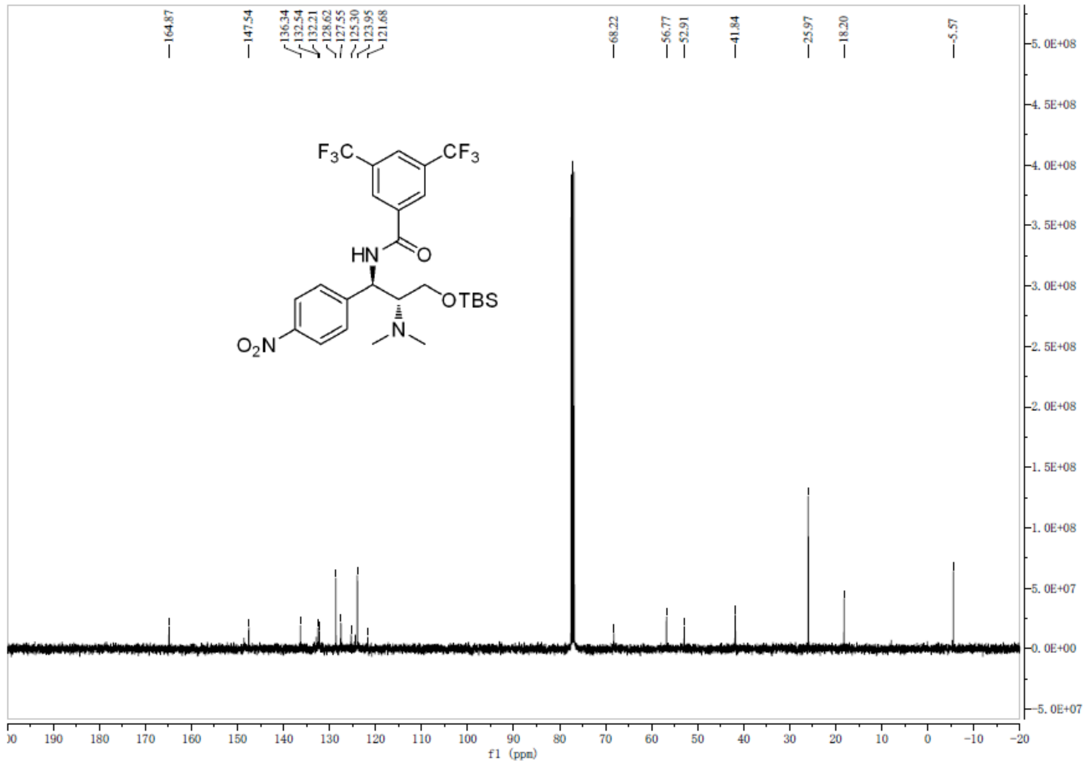

HRMS

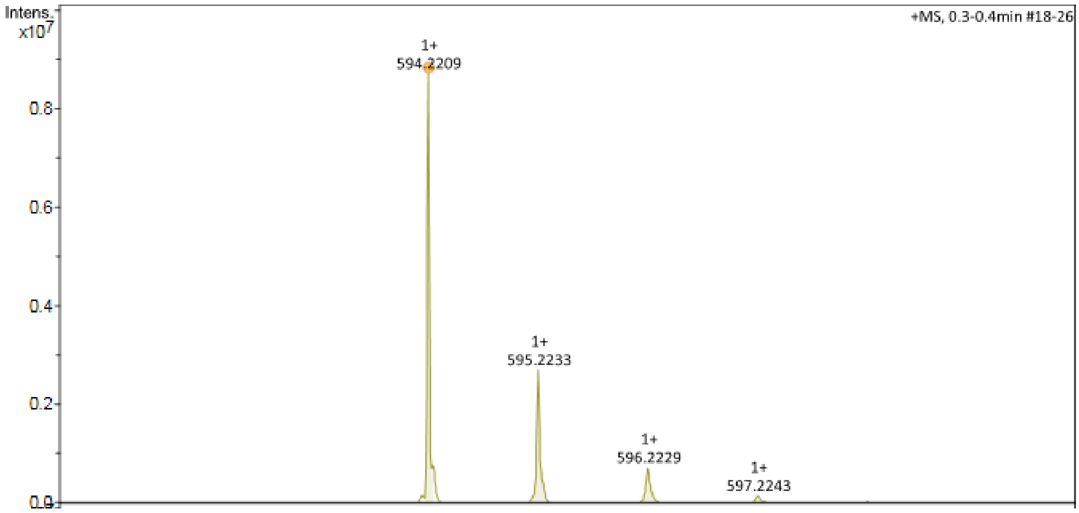

_N_-((1_R_,2_R_)-3-((_tert_-Butyldimethylsilyl)oxy)-1-(4-nitrophenyl)-2-(pyrrolidin-1-yl)-propyl)-3,5-bis(trifluoromethyl)benzamide (7n)

^{1}H NMR

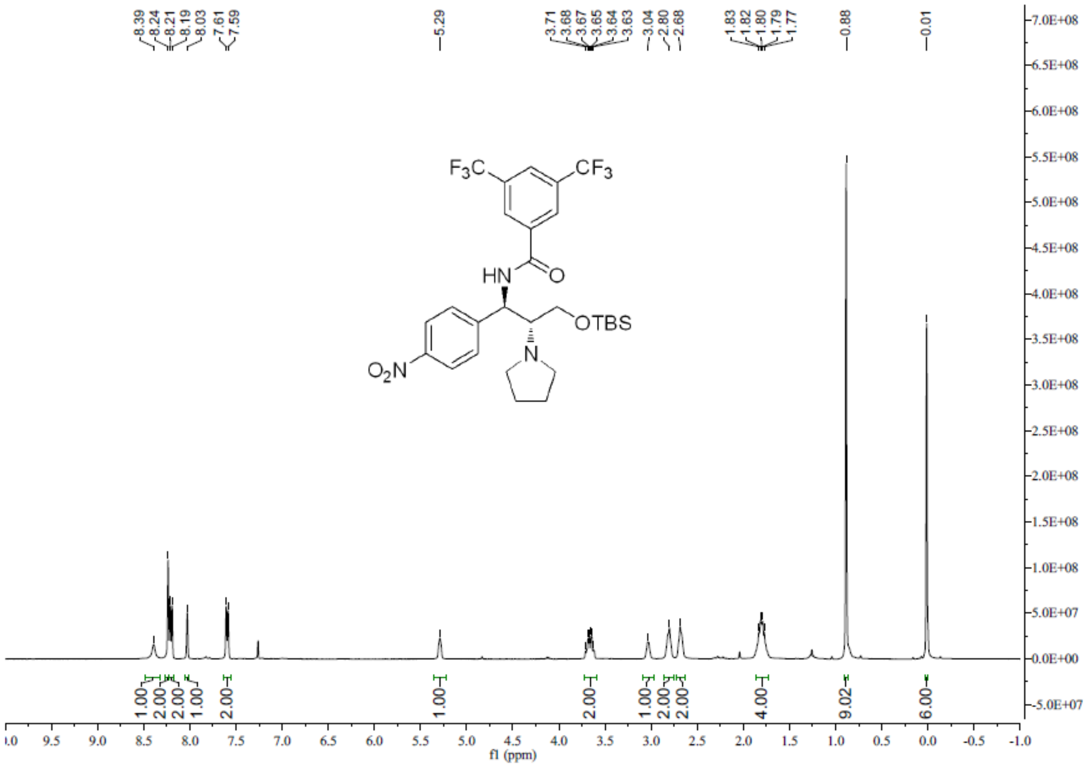

^{13}C NMR

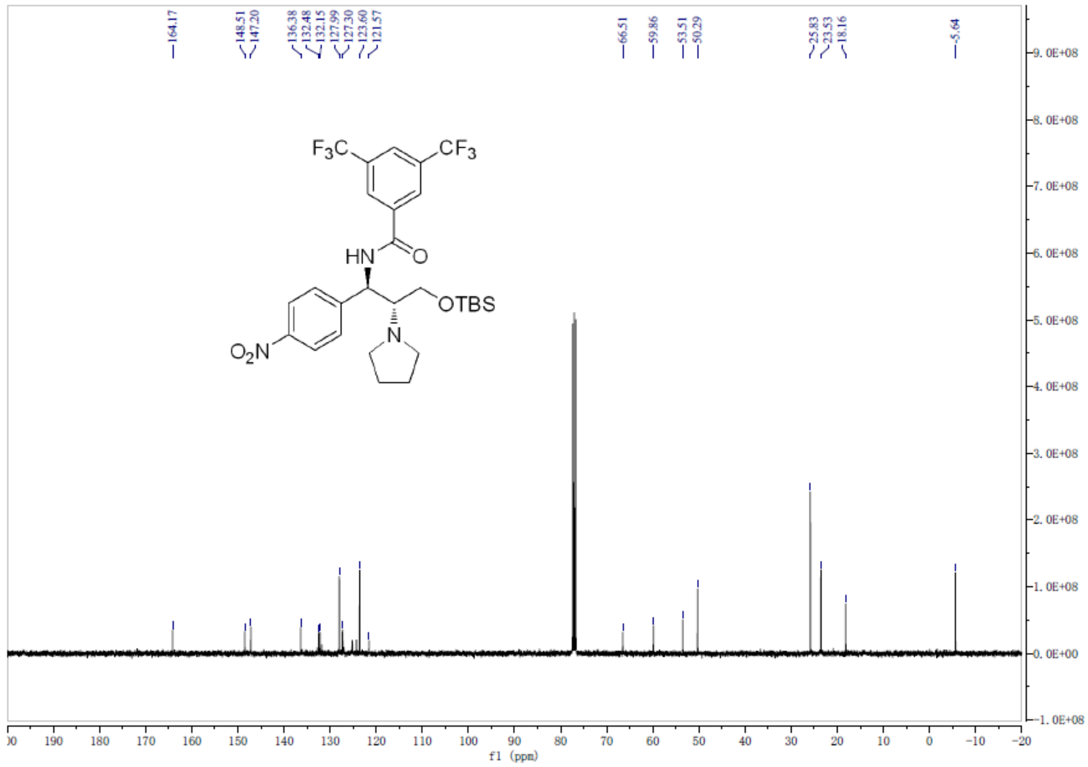

164.17
148.51
147.20
136.38
132.48
132.15
127.99
127.30
123.60
121.57
66.51
59.86
53.51
50.29
25.83
23.53
18.16
-5.64
F3C
CF3
HN
O
OTBS
N
O2N
f1 (ppm)

HRMS

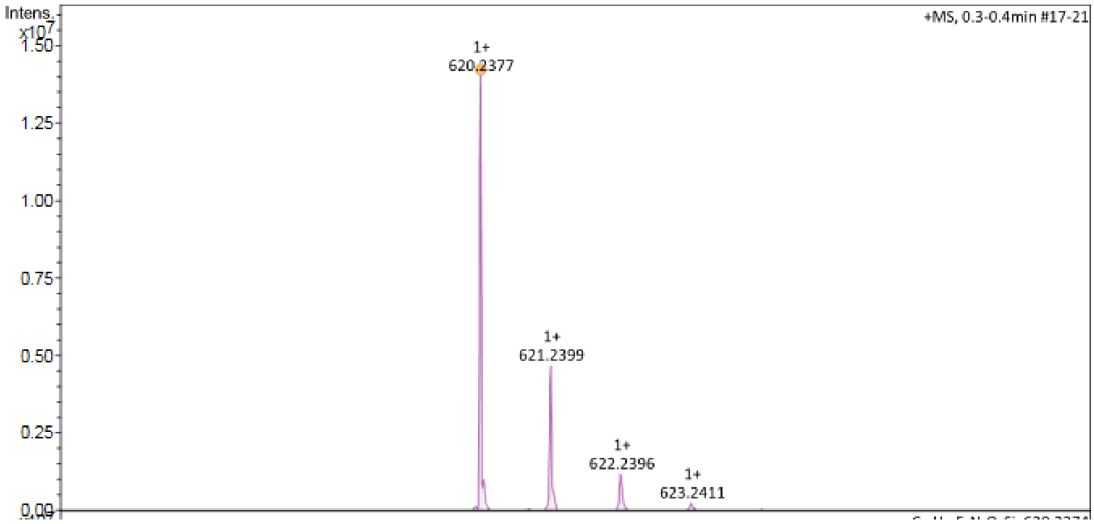

Intens.
x10^7
+MS, 0.3-0.4min #17-21
1+
620.2377
1+
621.2399
1+
622.2396
1+
623.2411

_N_-((1_R_,2_R_)-3-((_tert_-Butyldimethylsilyl)oxy)-1-(4-nitrophenyl)-2-(piperidin-1-yl)-propyl)-3,5-bis(trifluoromethyl)benzamide (7o)

^{1}H NMR

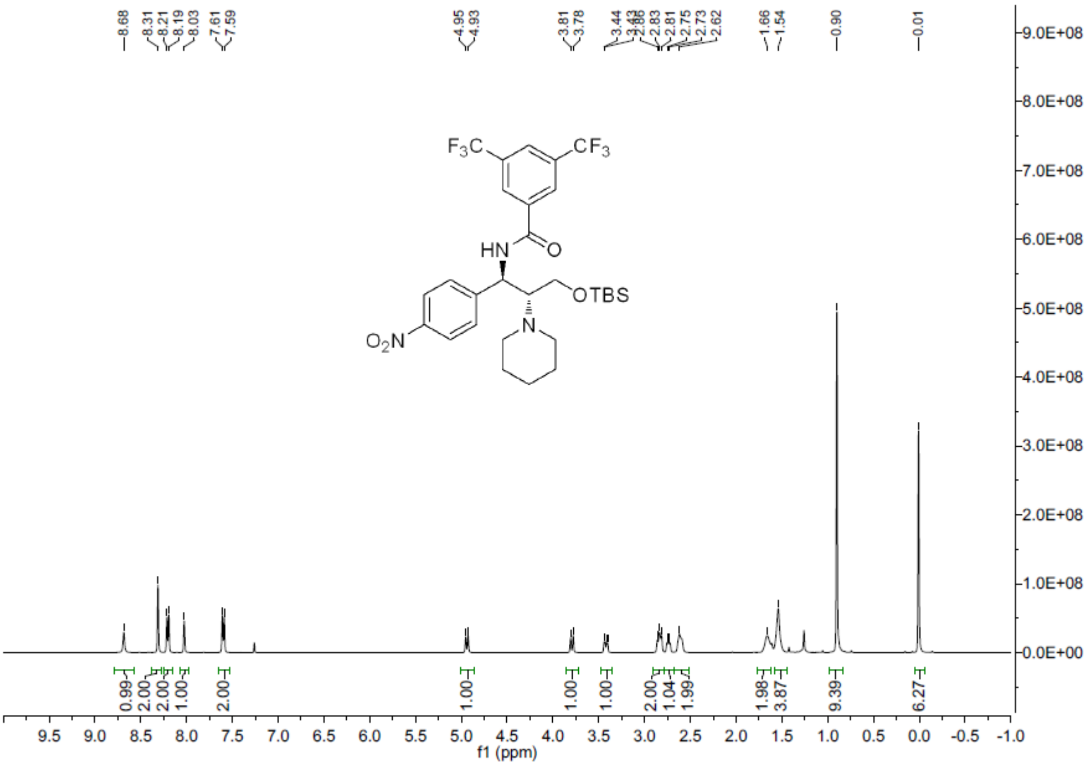

^{13}C NMR

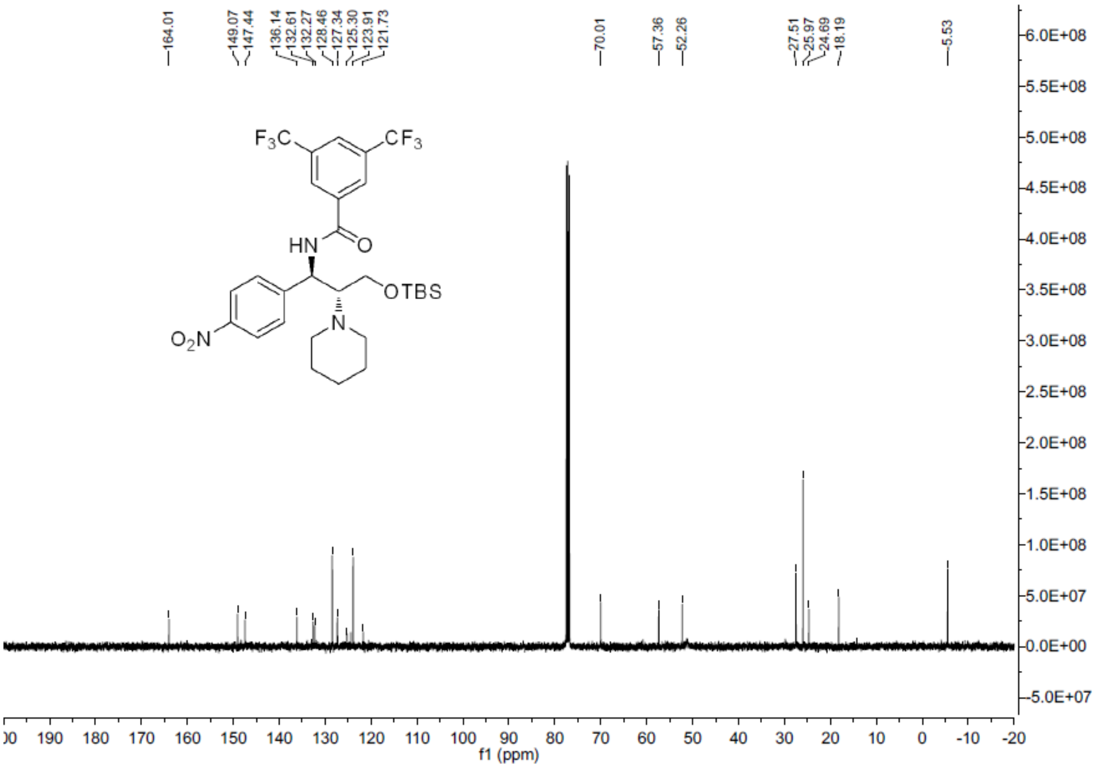

HRMS

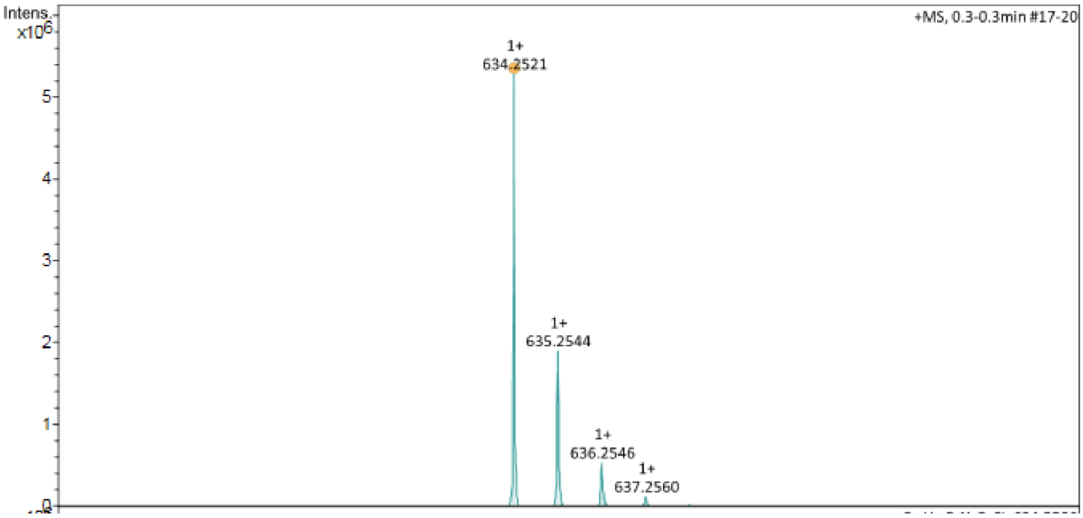

N-((1R,2R)-3-((*tert*-Butyldimethylsilyl)oxy)-2-morpholino-1-(4-nitrophenyl)-propyl)-3,5-bis(trifluoromethyl)benzamide (7p)

^{1}H NMR

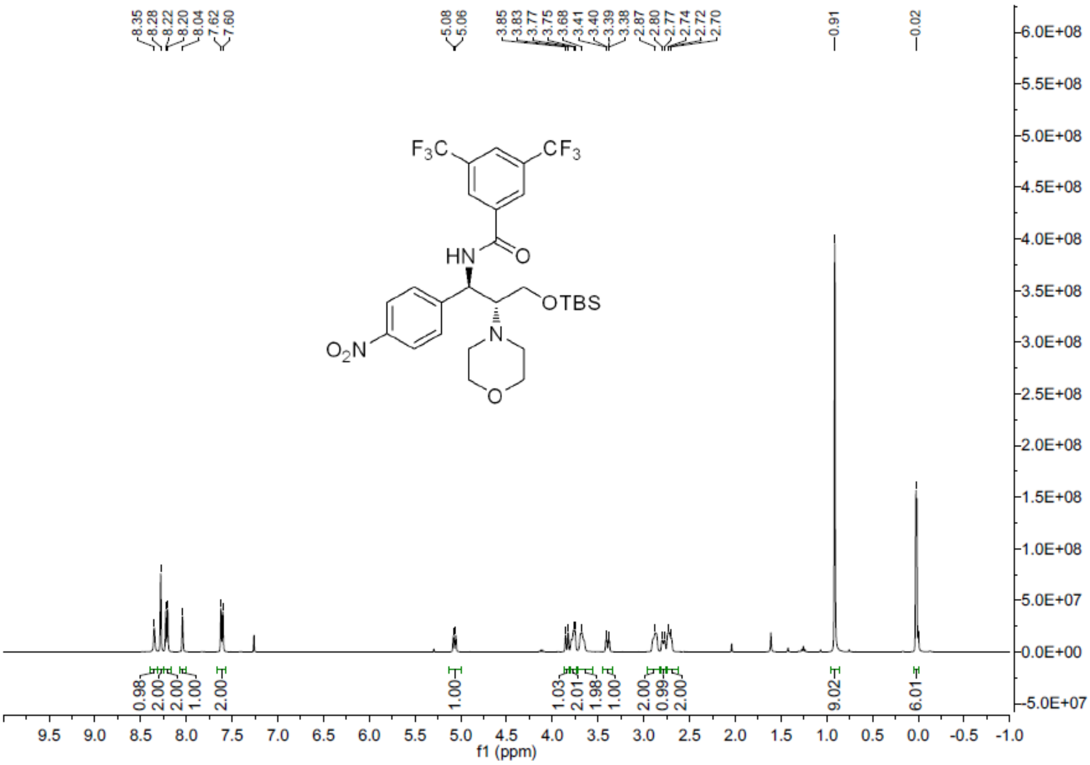

^{13}C NMR

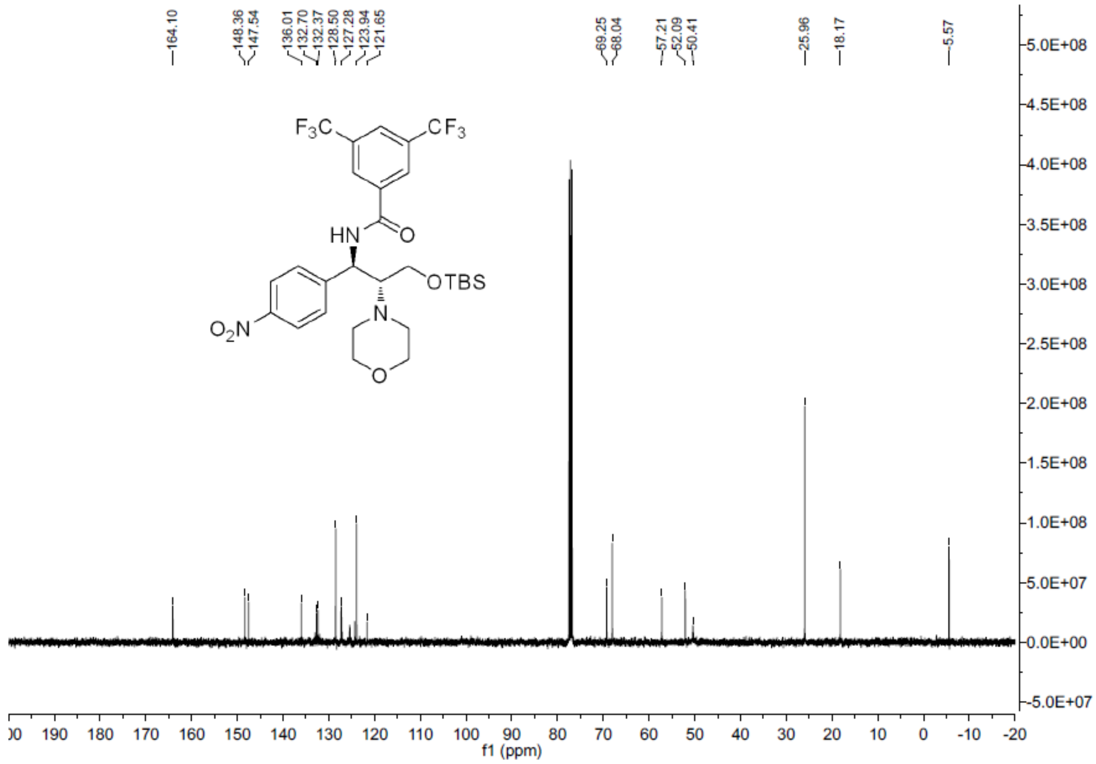

164.10
148.36
147.54
136.01
132.70
132.37
128.50
127.28
123.94
121.65
69.25
68.04
57.21
52.09
50.41
25.96
18.17
-5.57
F3C
CF3
HN
O
OTBS
N
O
O2N
f1 (ppm)

HRMS

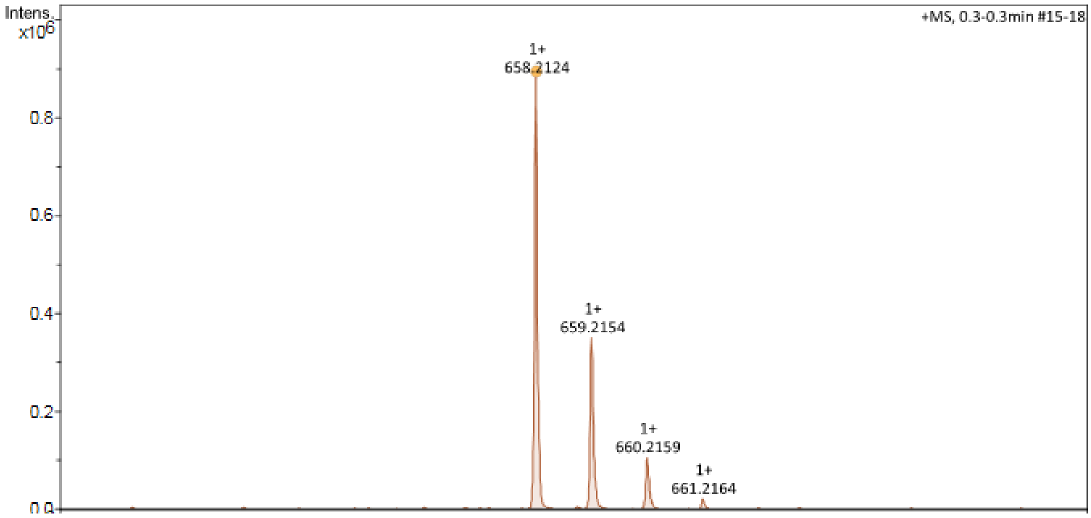

Intens.
x10^6
+MS, 0.3-0.3min #15-18
1+
658.2124
1+
659.2154
1+
660.2159
1+
661.2164

(1*S*,6*R*)-6-(Methoxycarbonyl)cyclohex-3-enecarboxylic acid (9a)

^{1}H NMR

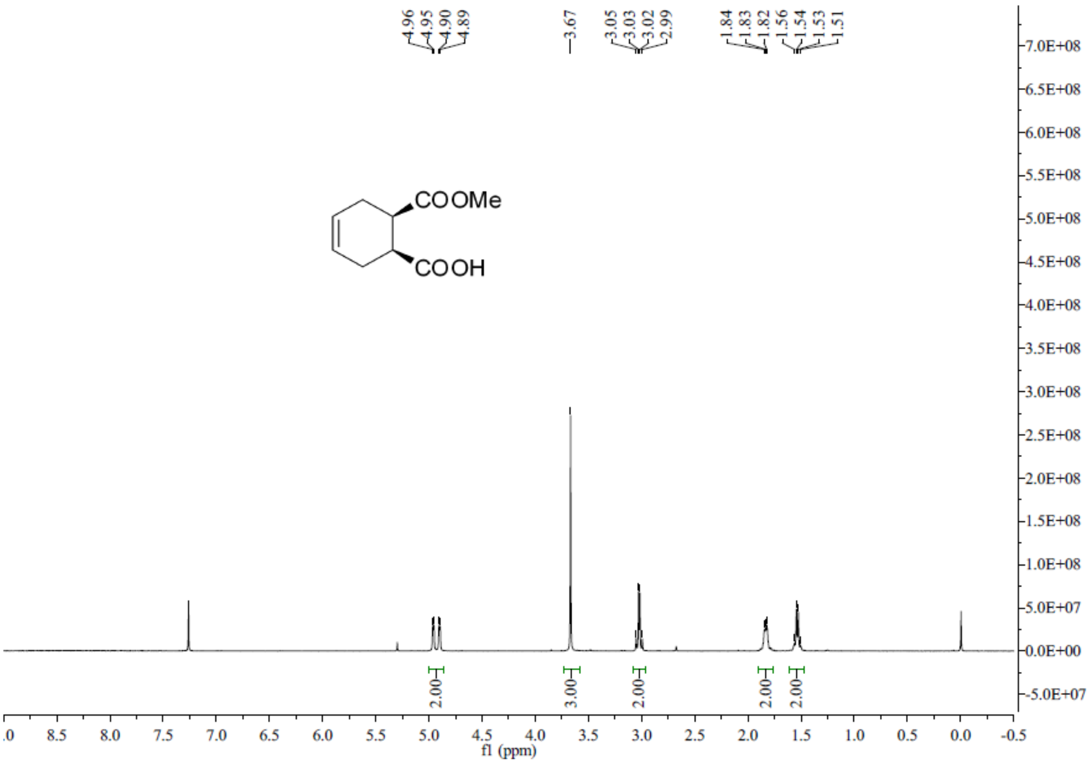

^{13}C NMR

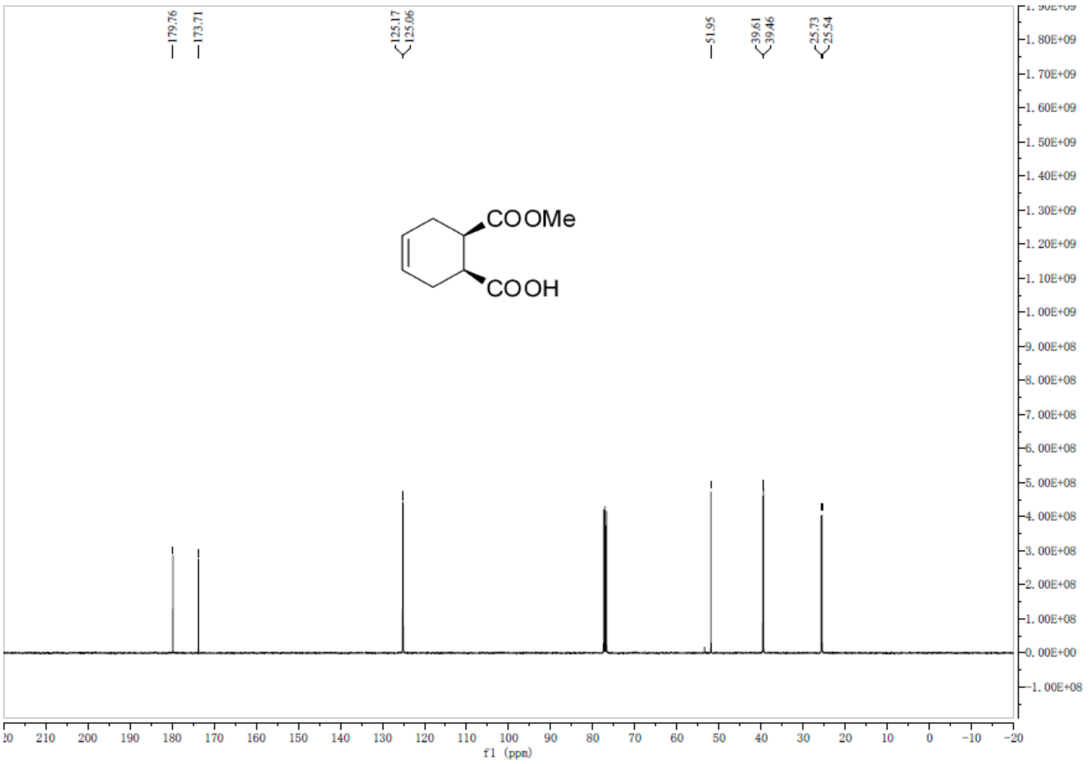

(1*S*,2*R*)-2-(Methoxycarbonyl)cyclohexanecarboxylic acid (9b)

^{1}H NMR

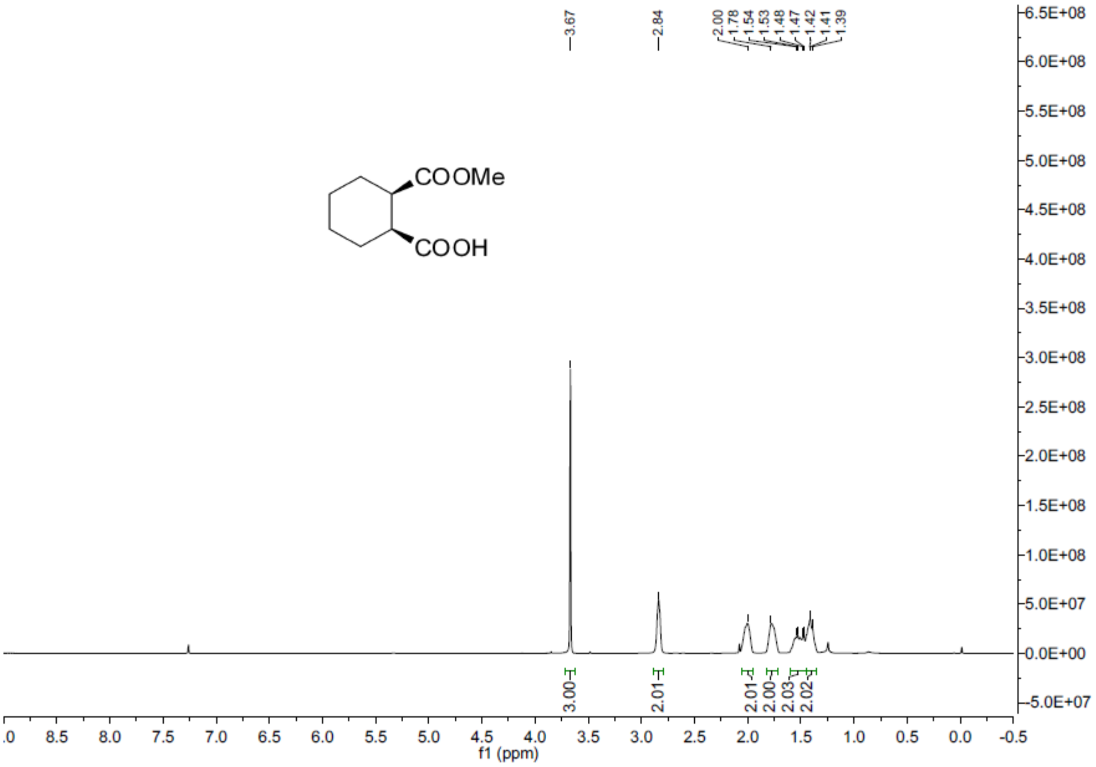

^{13}C NMR

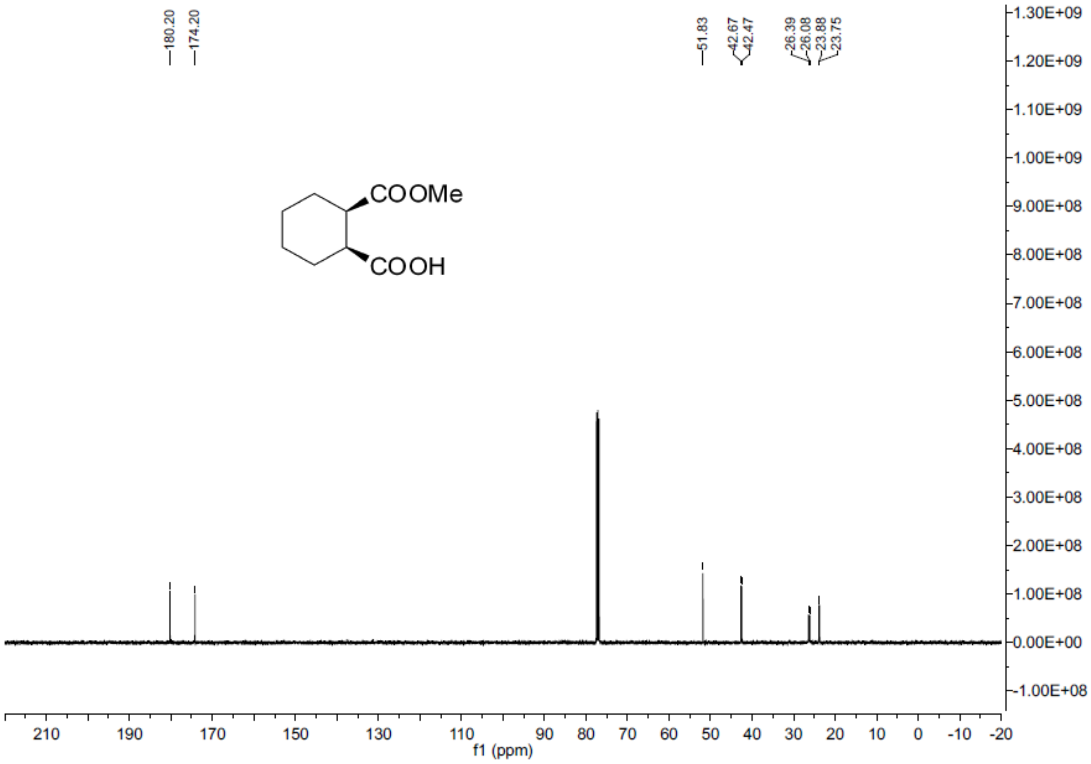

(1*S*,2*R*,3*S*,4*R*)-3-(Methoxycarbonyl)-7-oxabicyclo[2.2.1]hept-5-ene-2-carboxylic acid (9c)

^{1}H NMR

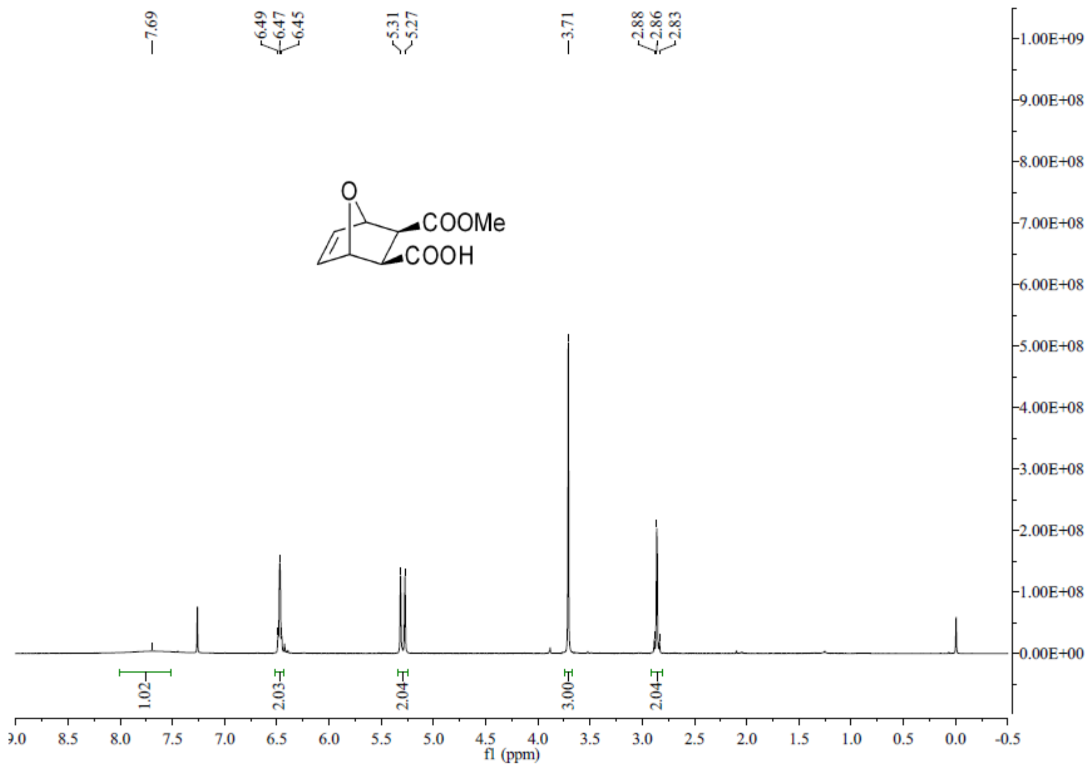

^{13}C NMR

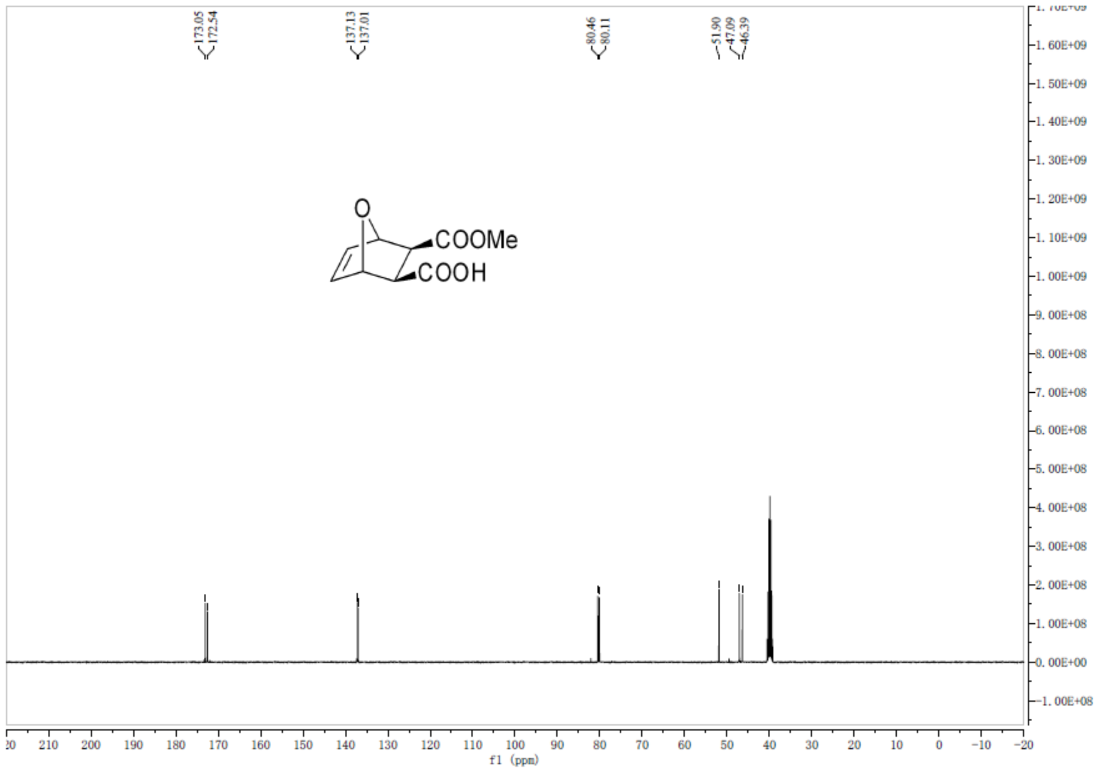

(1*R*,2*S*,3*R*,4*S*)-3-(Methoxycarbonyl)bicyclo[2.2.1]hept-5-ene-2-carboxylic acid (9d)

^{1}H NMR

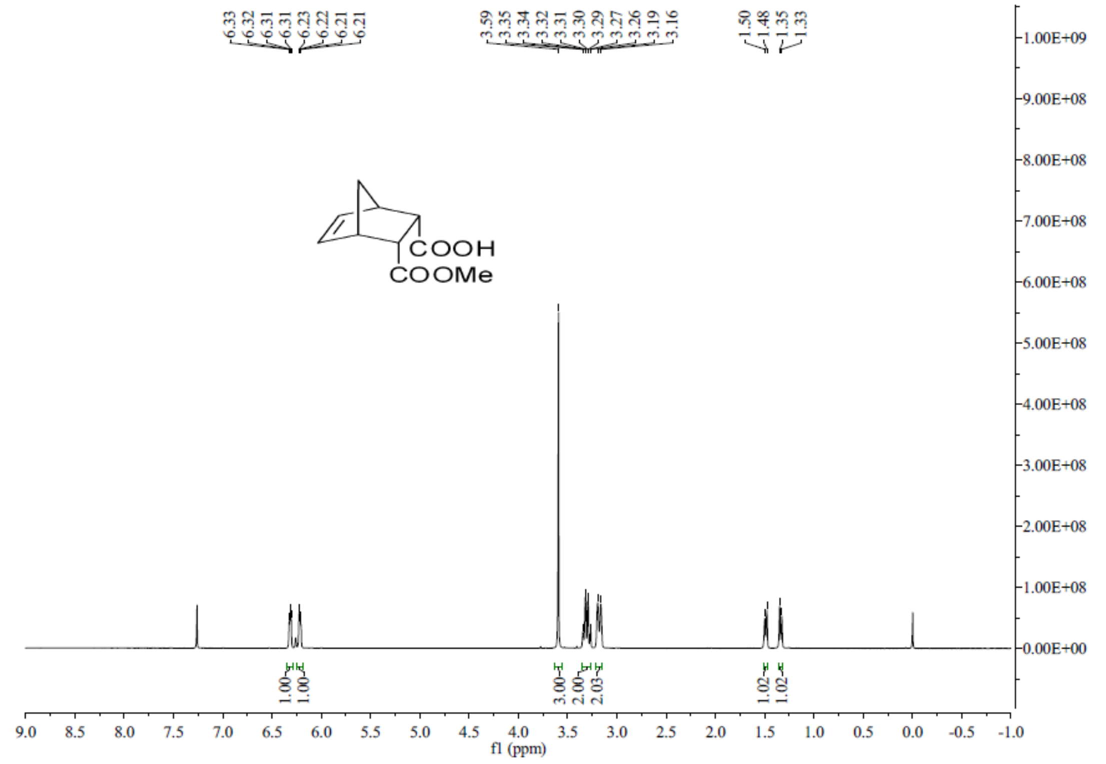

^{13}C NMR

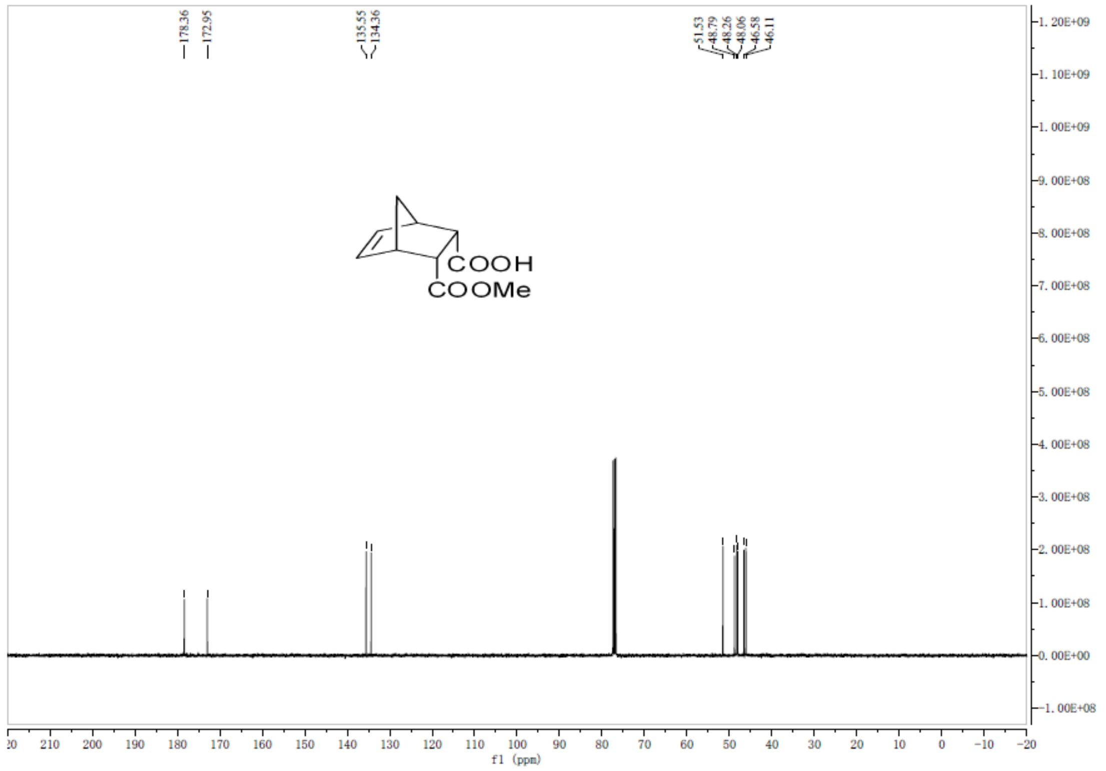

(*R*)-5-Methoxy-3-methyl-5-oxopentanoic acid (9e)

^{1}H NMR

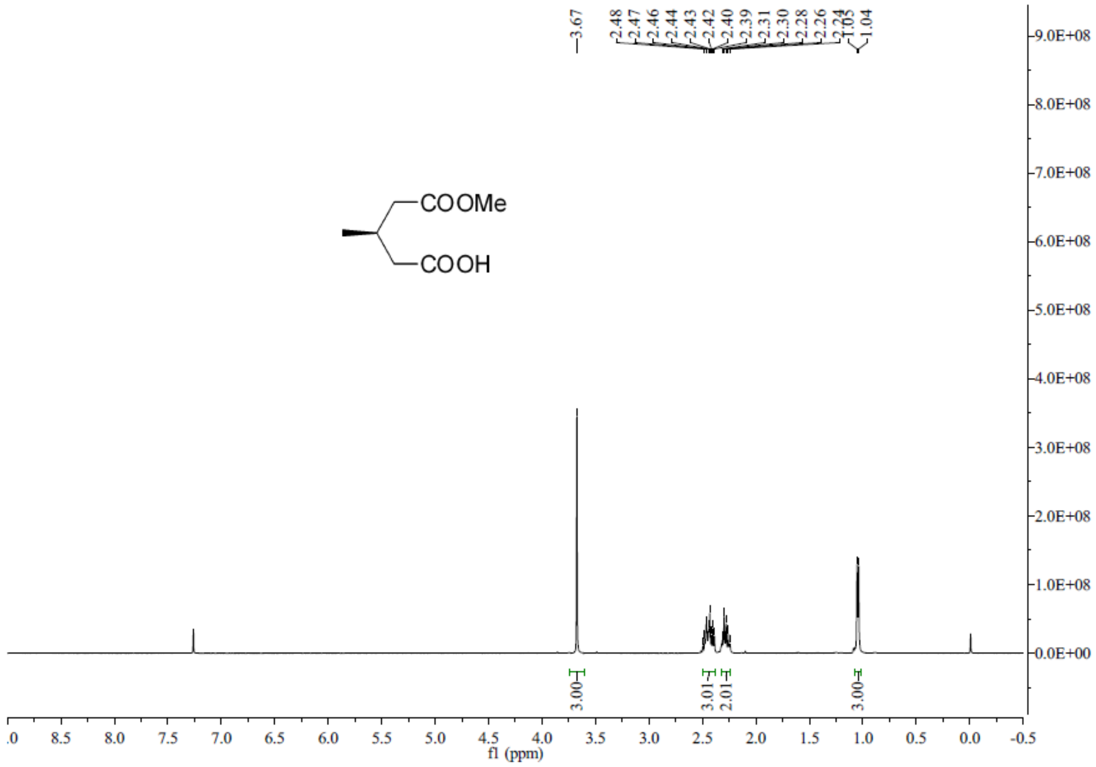

^{13}C NMR

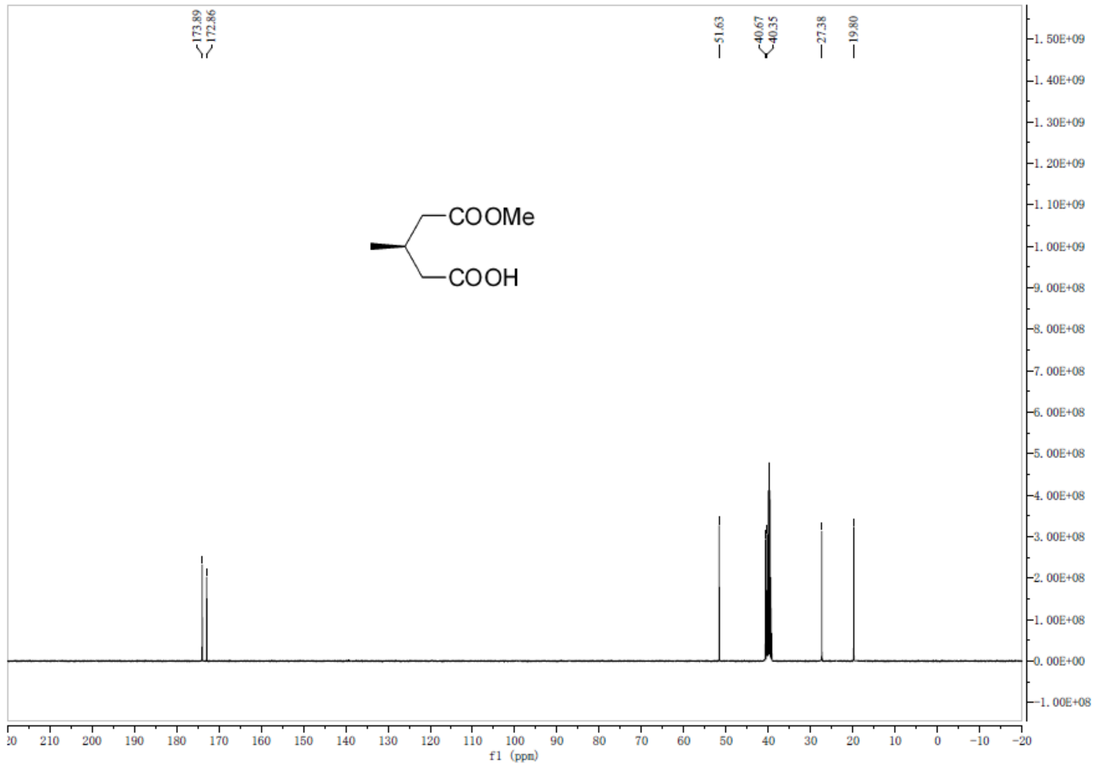

(*R*)-3-Ethyl-5-methoxy-5-oxopentanoic acid (9f)

^{1}H NMR

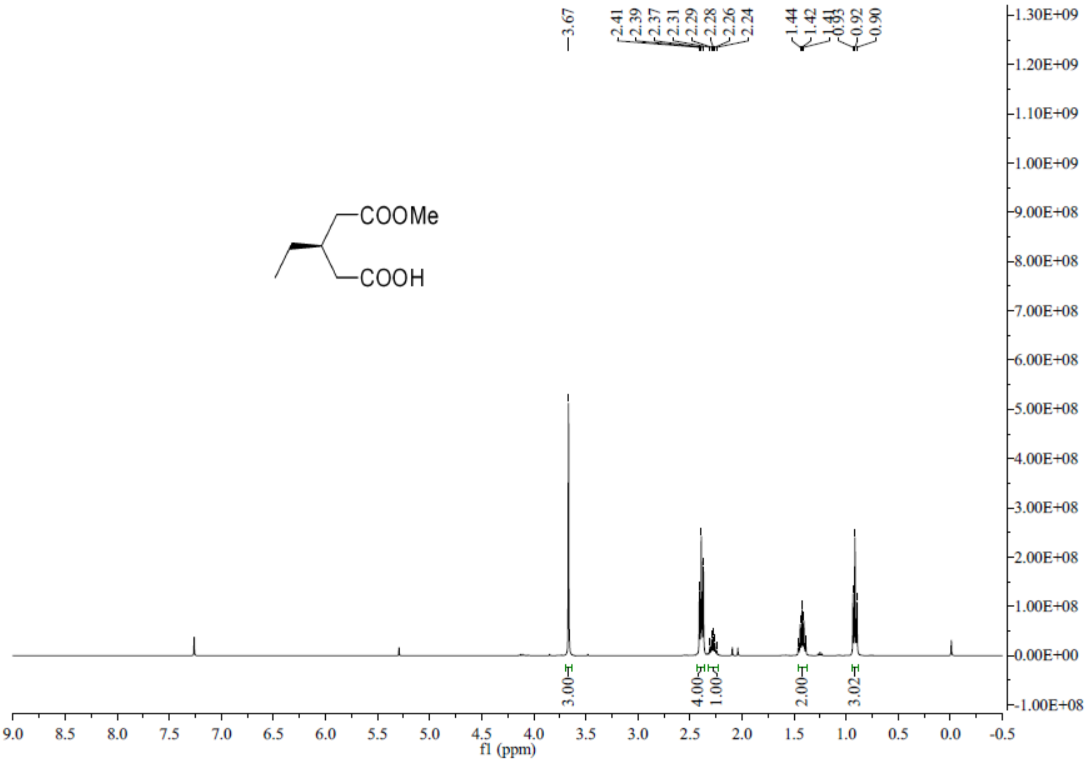

^{13}C NMR

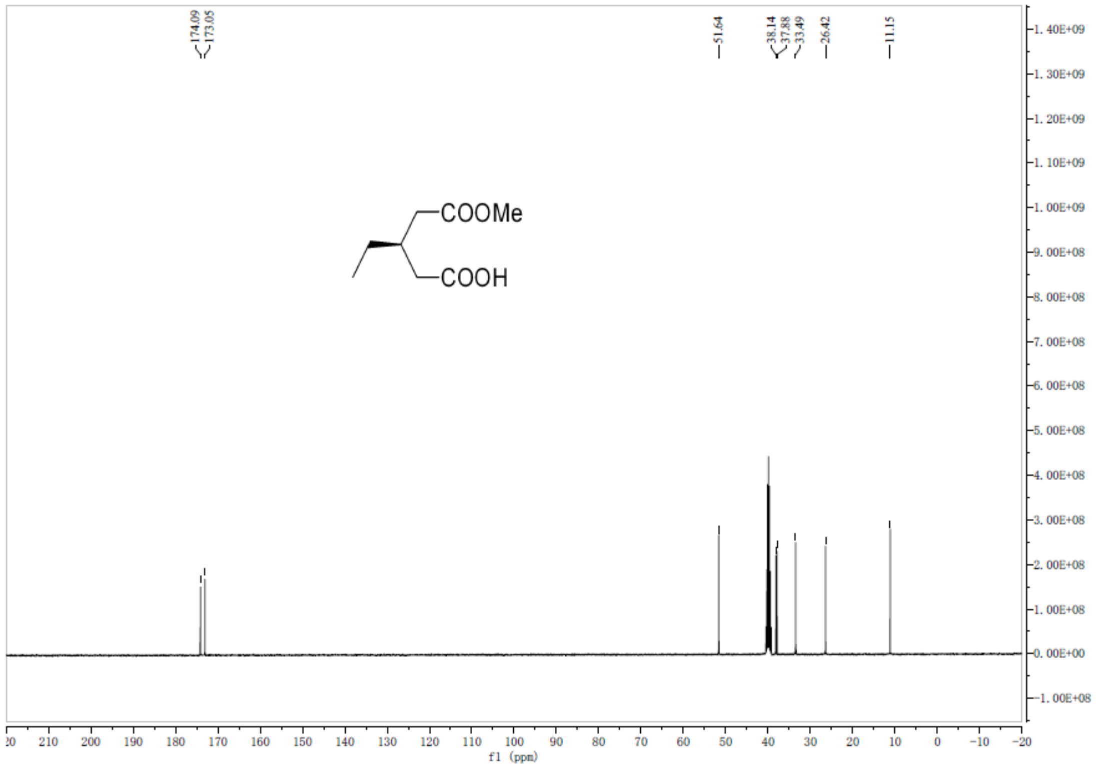

(*R*)-3-Isopropyl-5-methoxy-5-oxopentanoic acid (9g)

^{1}H NMR

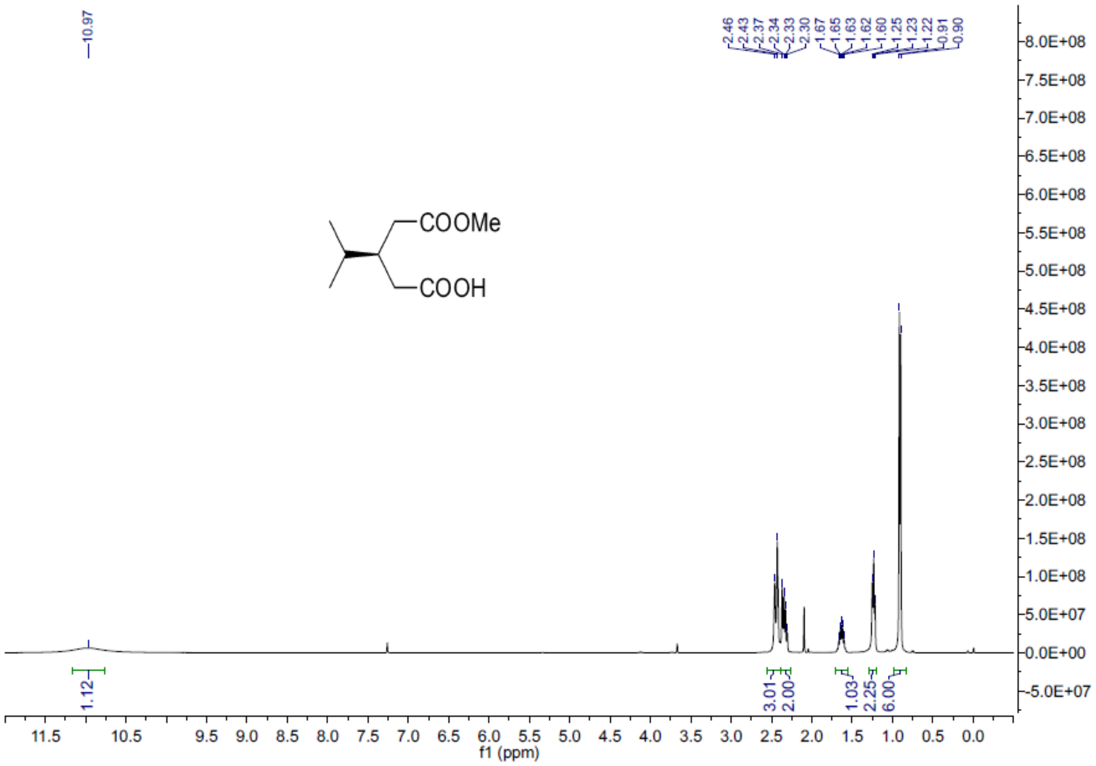

^{13}C NMR

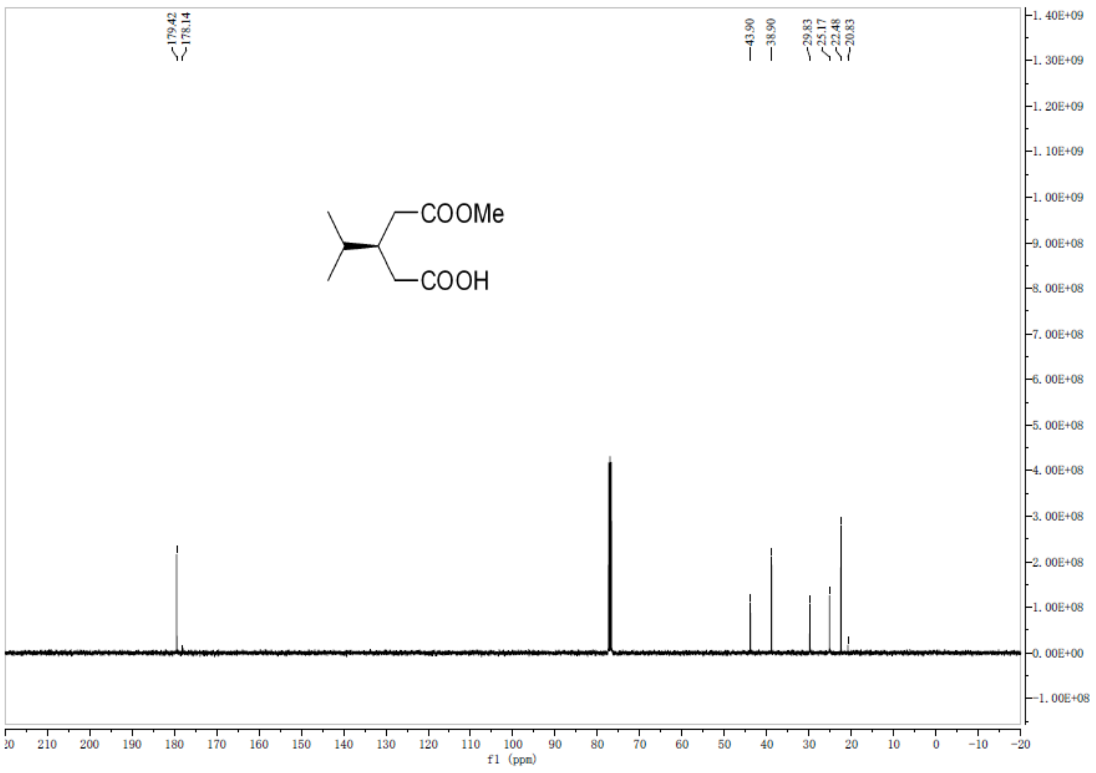

(*R*)-3-(Allyloxy)-5-methoxy-5-oxopentanoic acid (9h)

^{1}H NMR

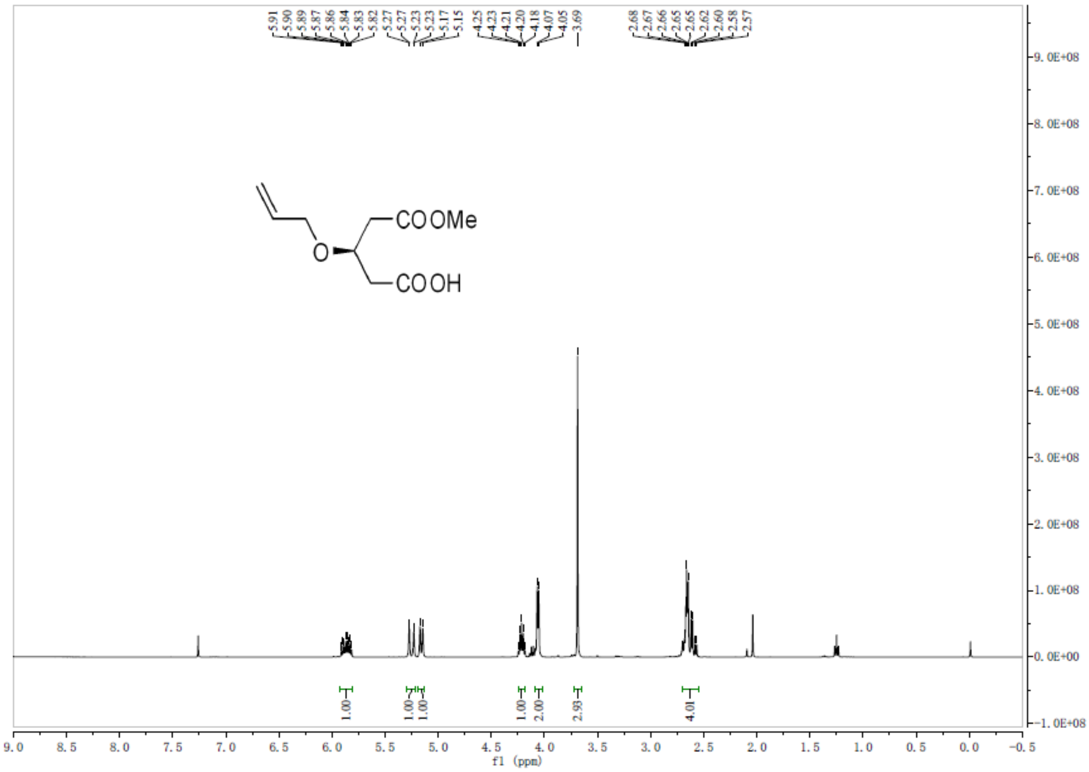

^{13}C NMR

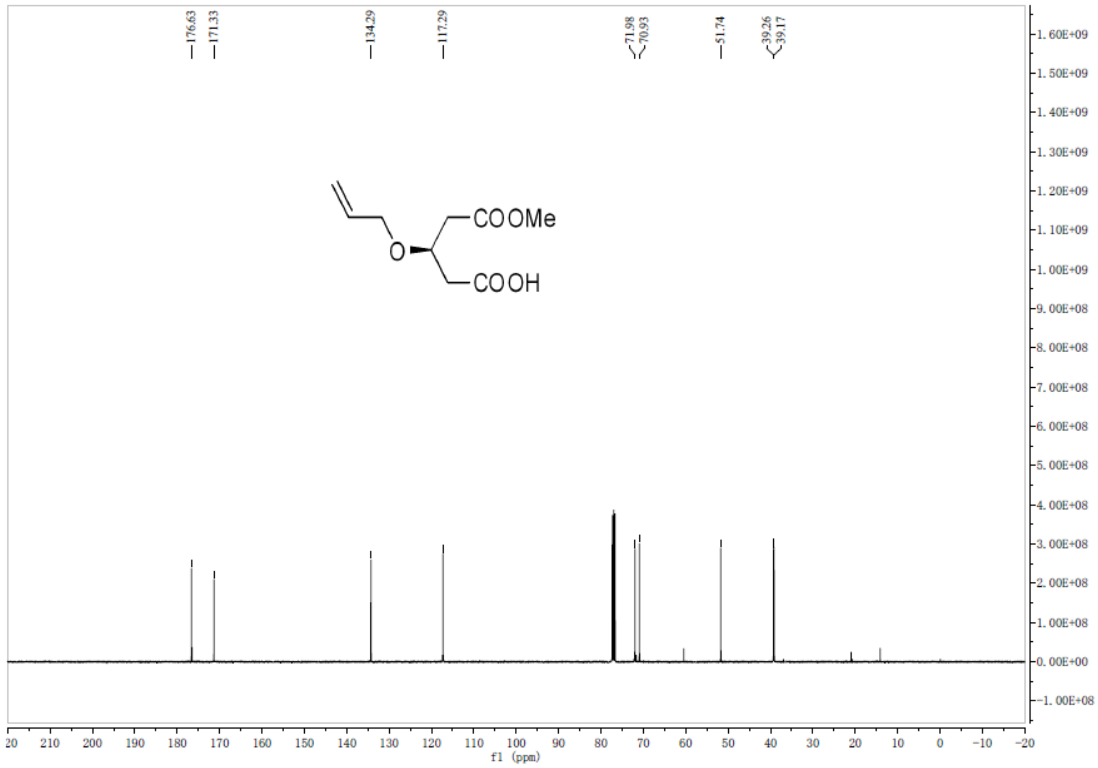

HRMS

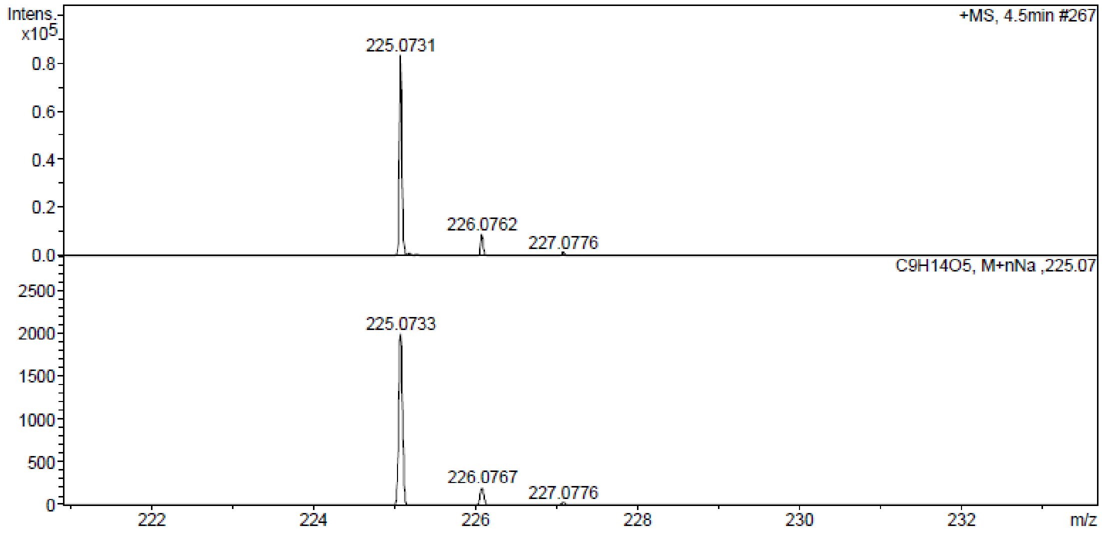

(*R*)-5-Methoxy-5-oxo-3-phenylpentanoic acid (9i)

^{1}H NMR

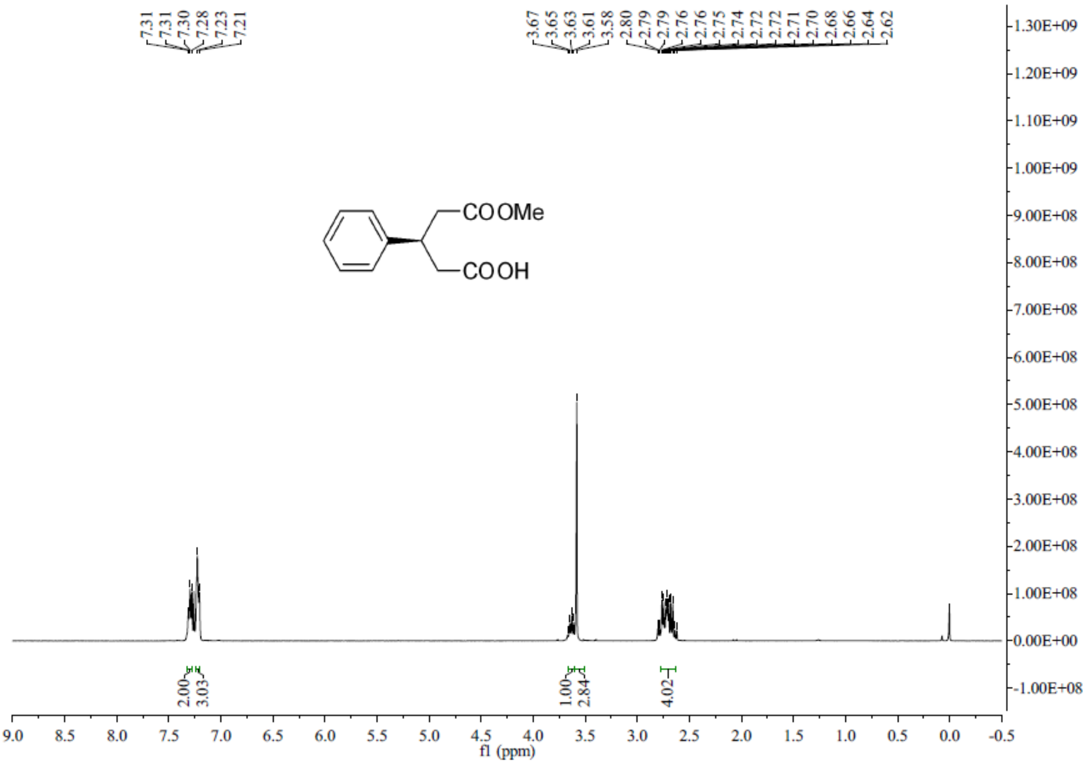

^{13}C NMR

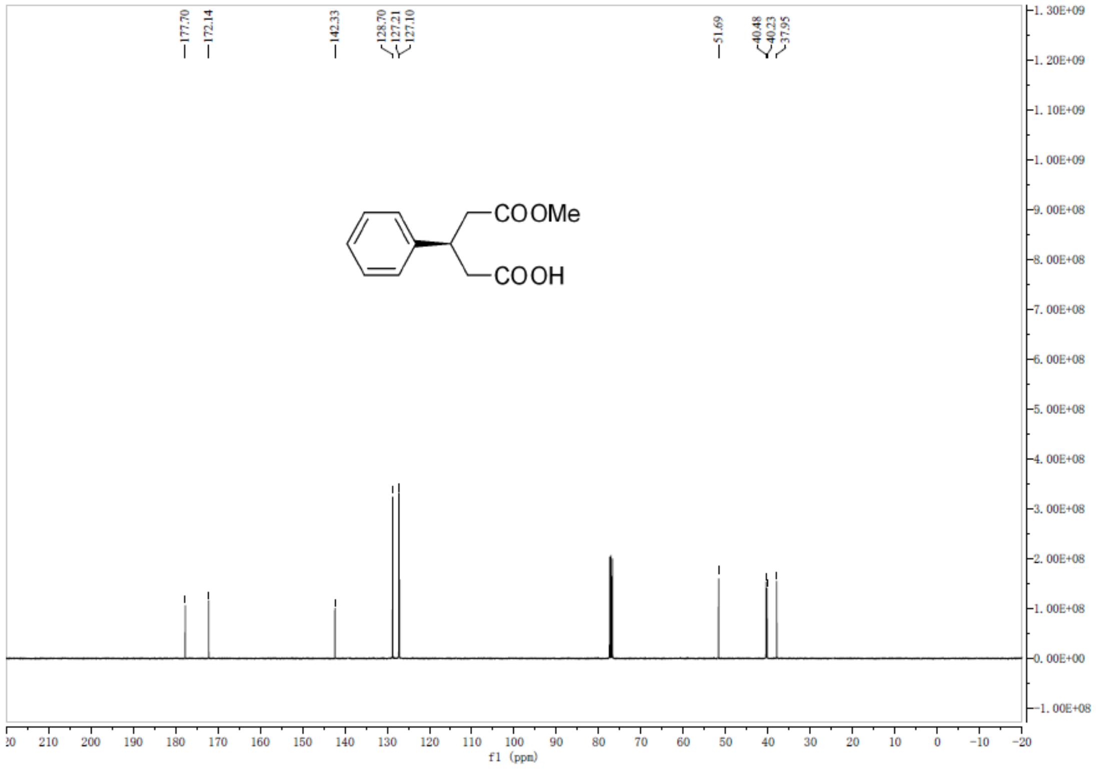

(*R*)-3-(4-chlorophenyl)-5-methoxy-5-oxopentanoic acid (9j)

^{1}H NMR

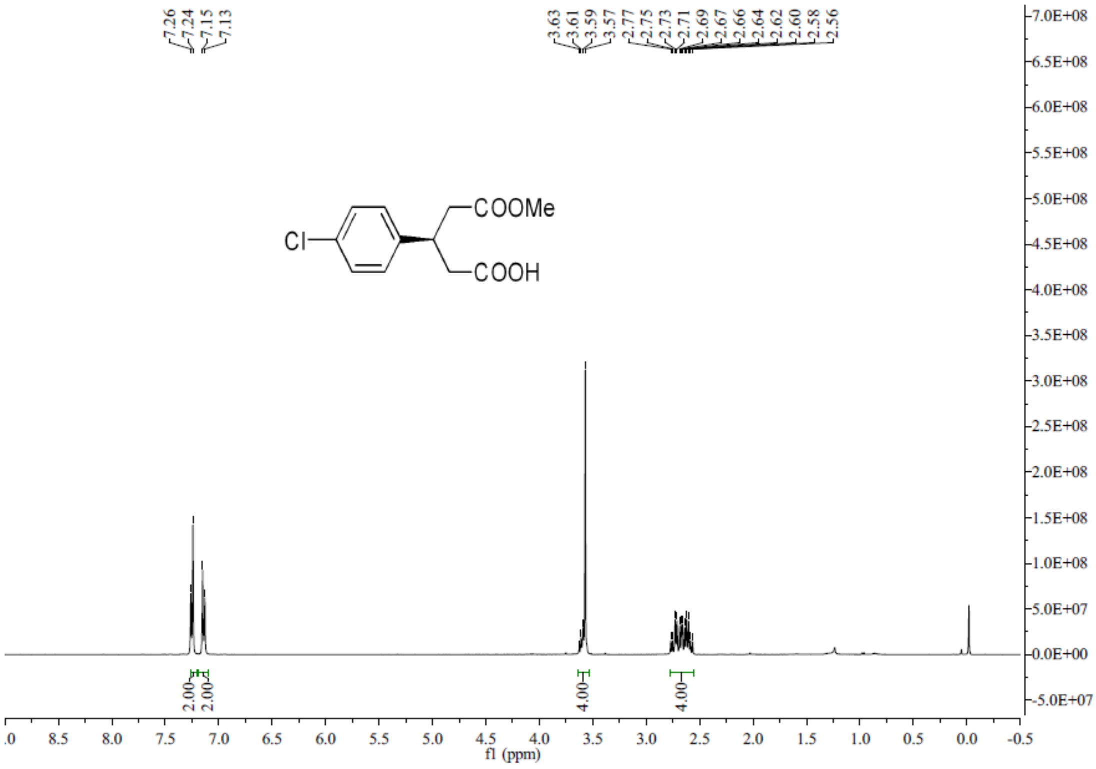

^{13}C NMR

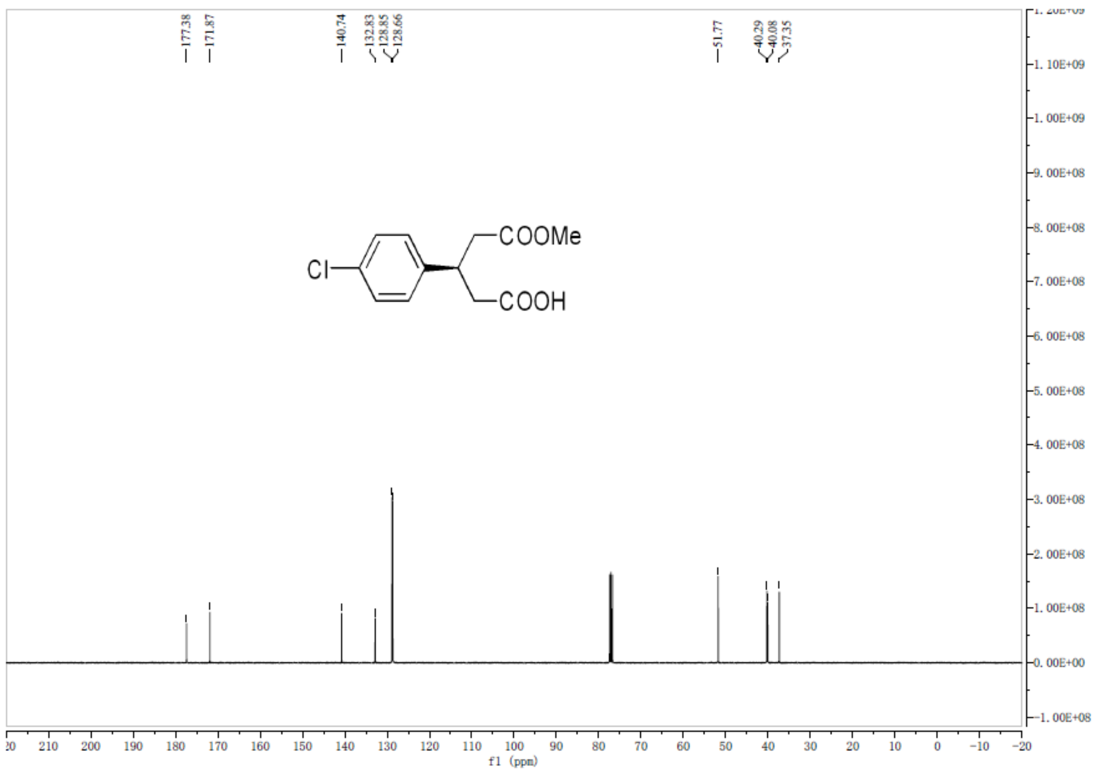

(*R*)-5-Methoxy-5-oxo-3-(p-tolyl)pentanoic acid (9k)

^{1}H NMR

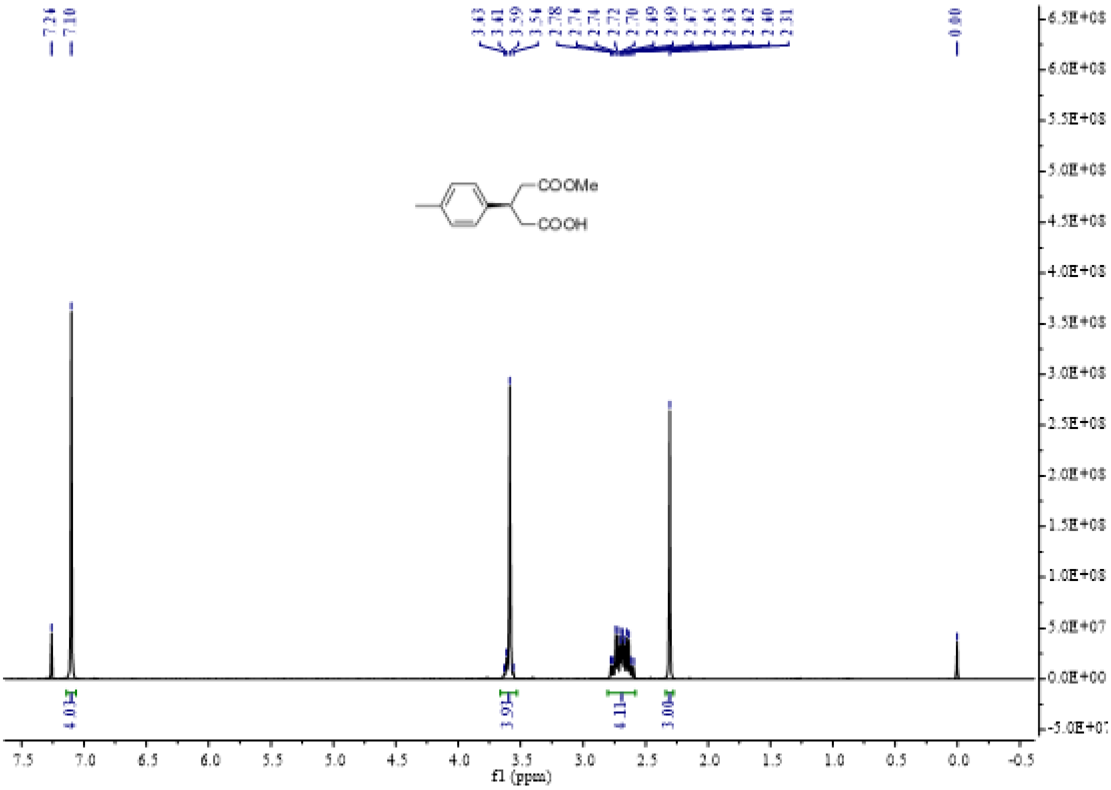

^{13}C NMR

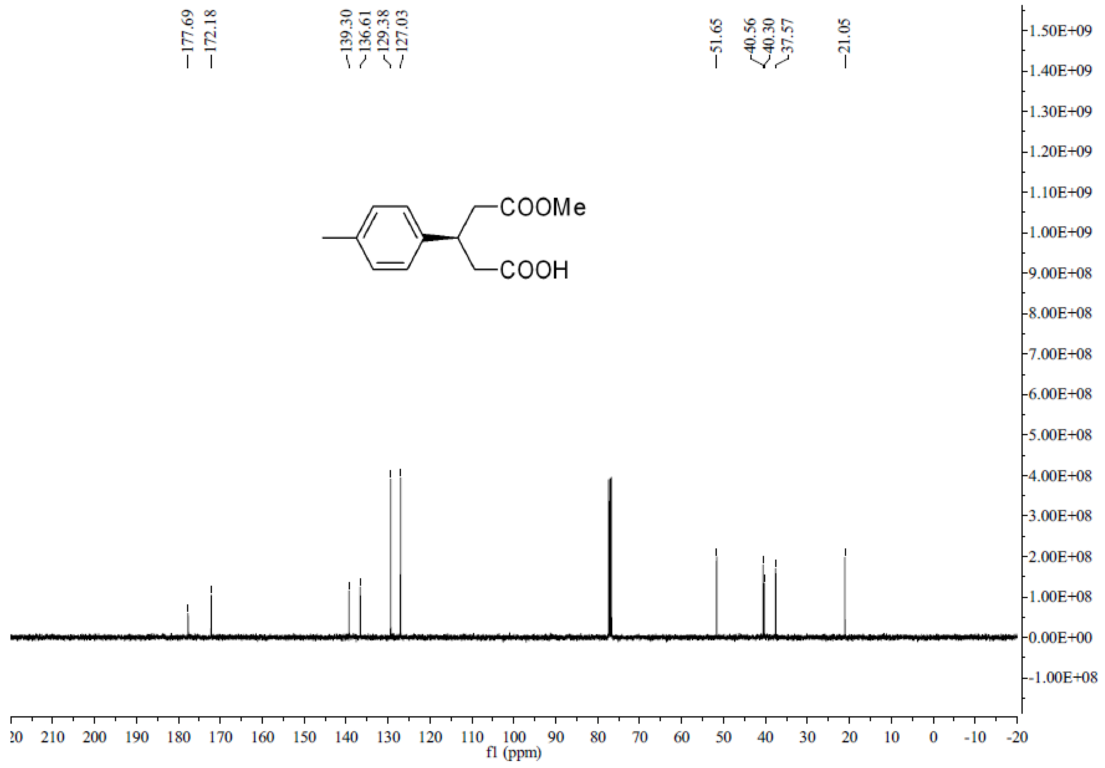

(*R*)-5-Methoxy-3-(4-methoxyphenyl)-5-oxopentanoic acid (9l)

^{1}H NMR

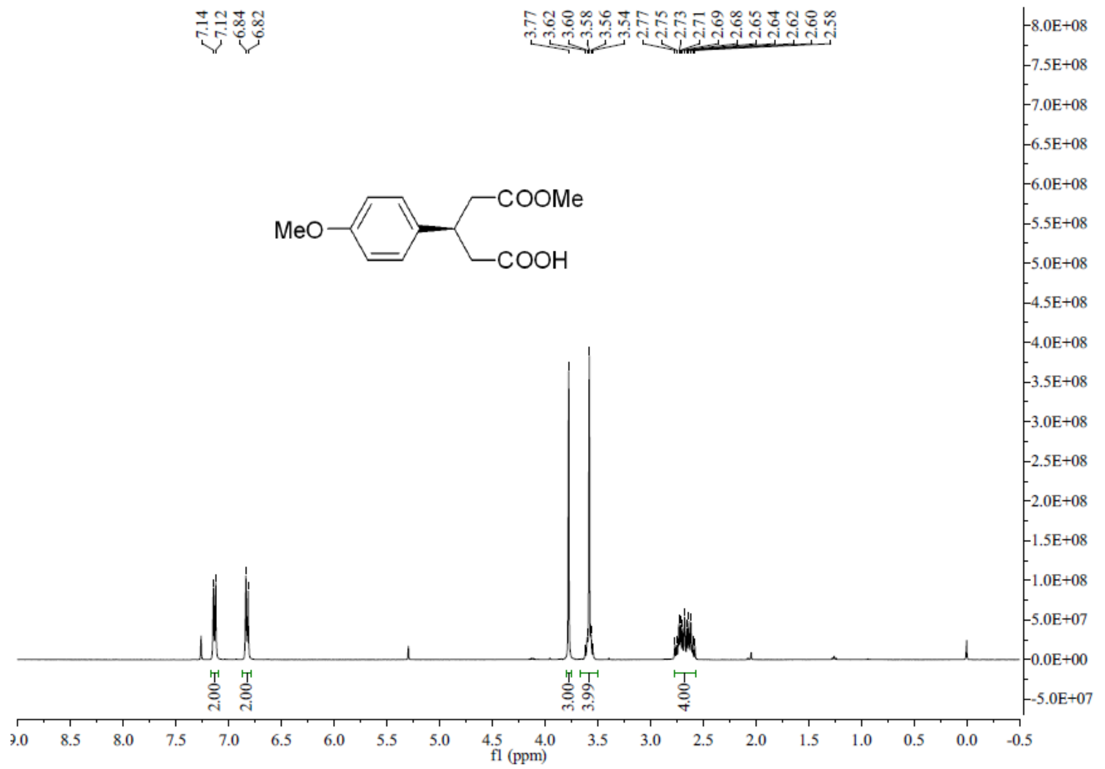

^{13}C NMR

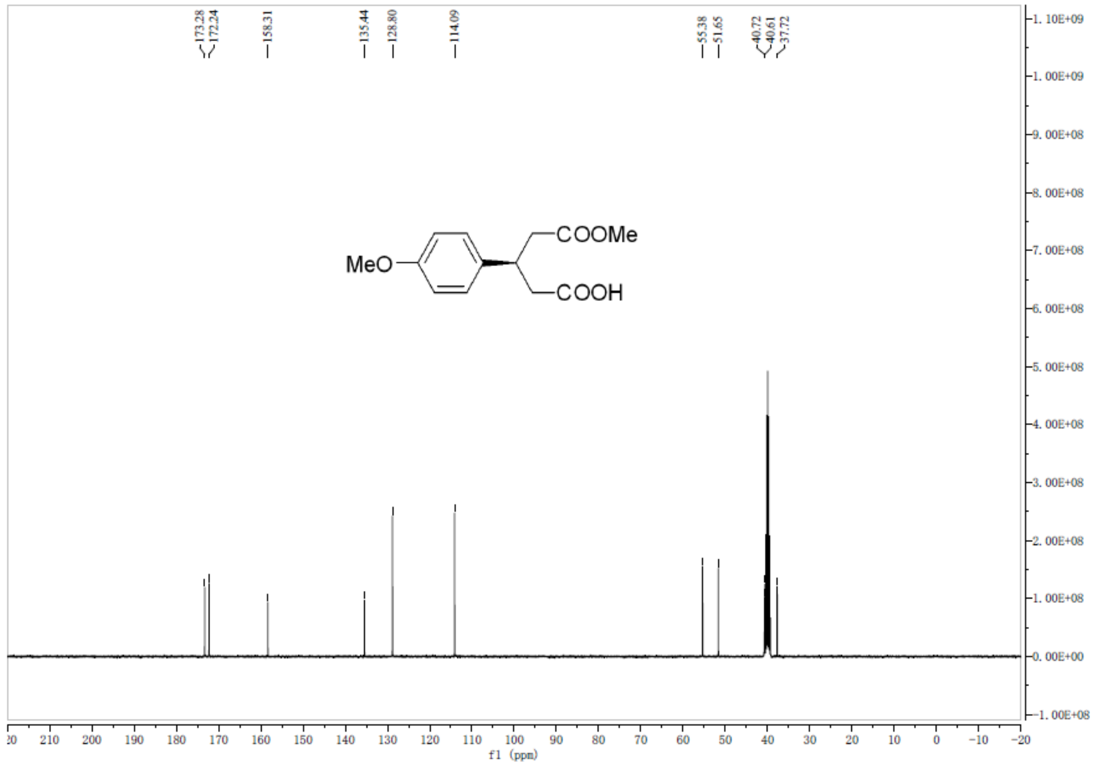

(1*S*,6*R*)-6-(Ethoxycarbonyl)cyclohex-3-enecarboxylic acid (9m)

^{1}H NMR

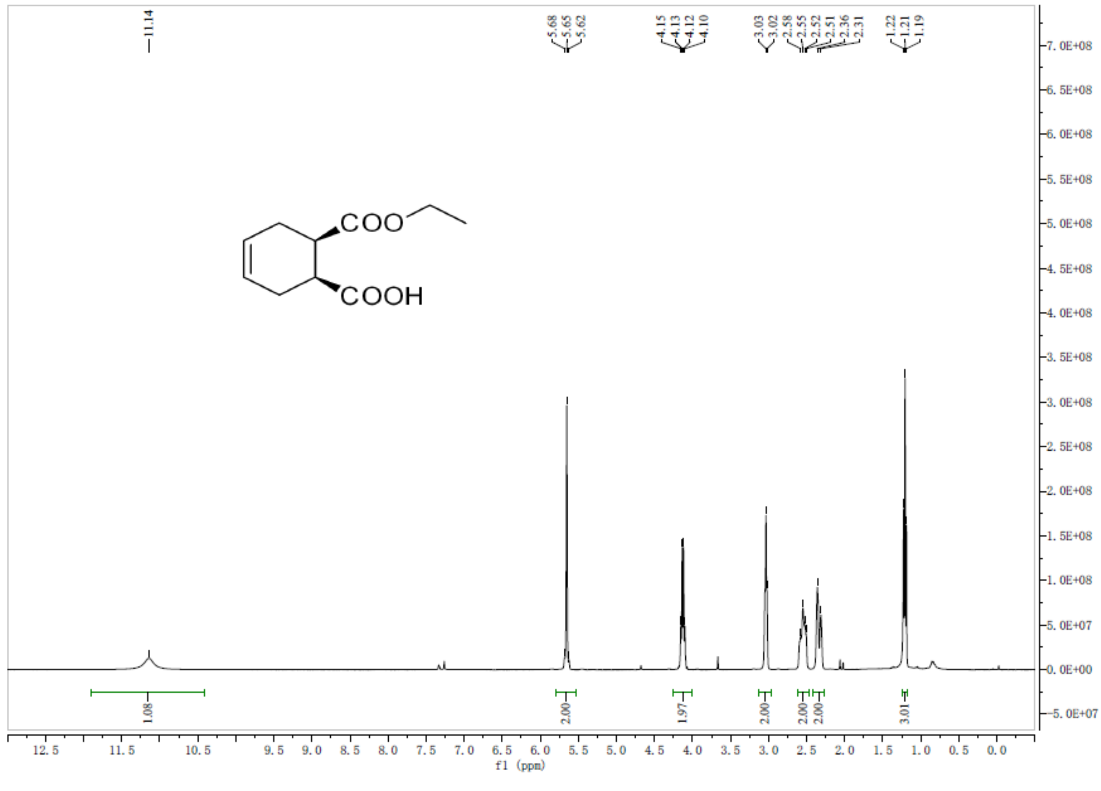

^{13}C NMR

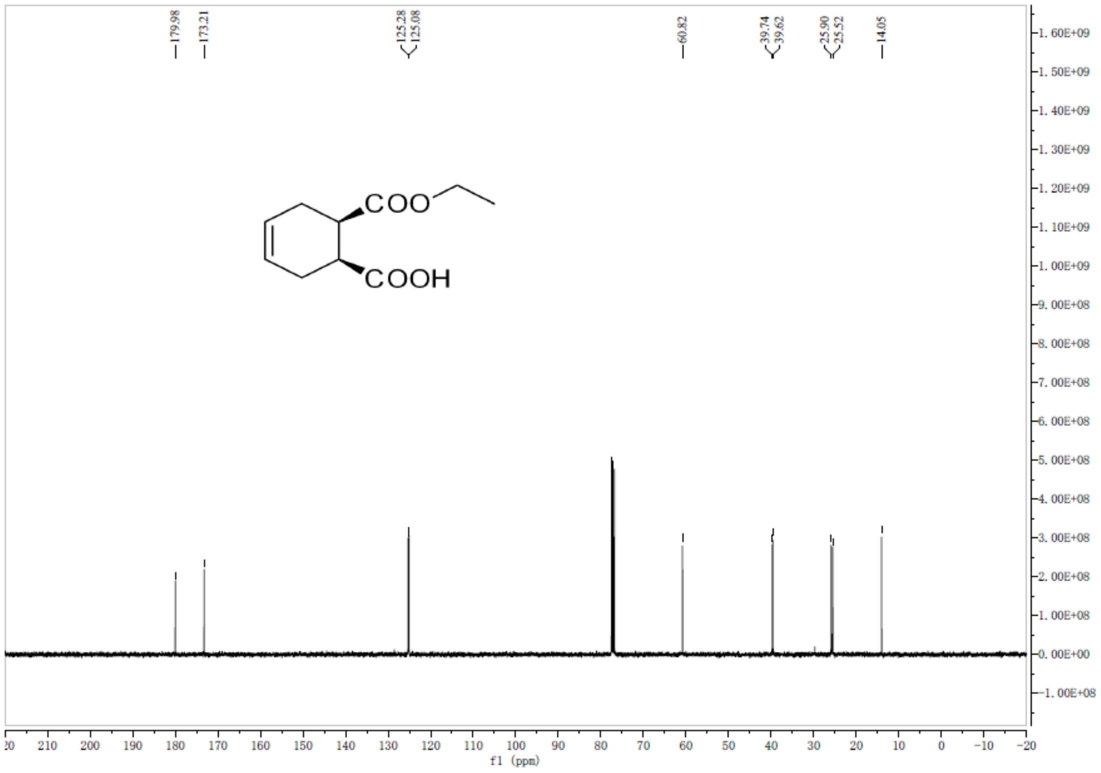

(1*S*,6*R*)-6-(Isopropoxycarbonyl)cyclohex-3-enecarboxylic acid (9n)

^{1}H NMR

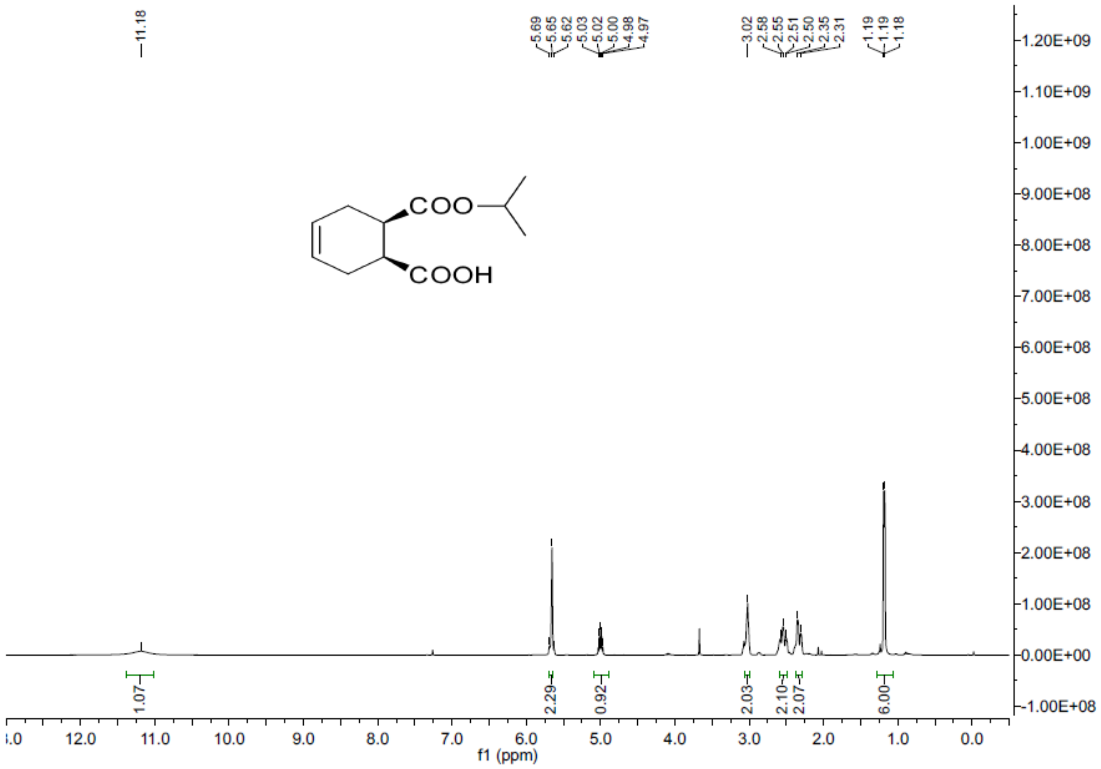

^{13}C NMR

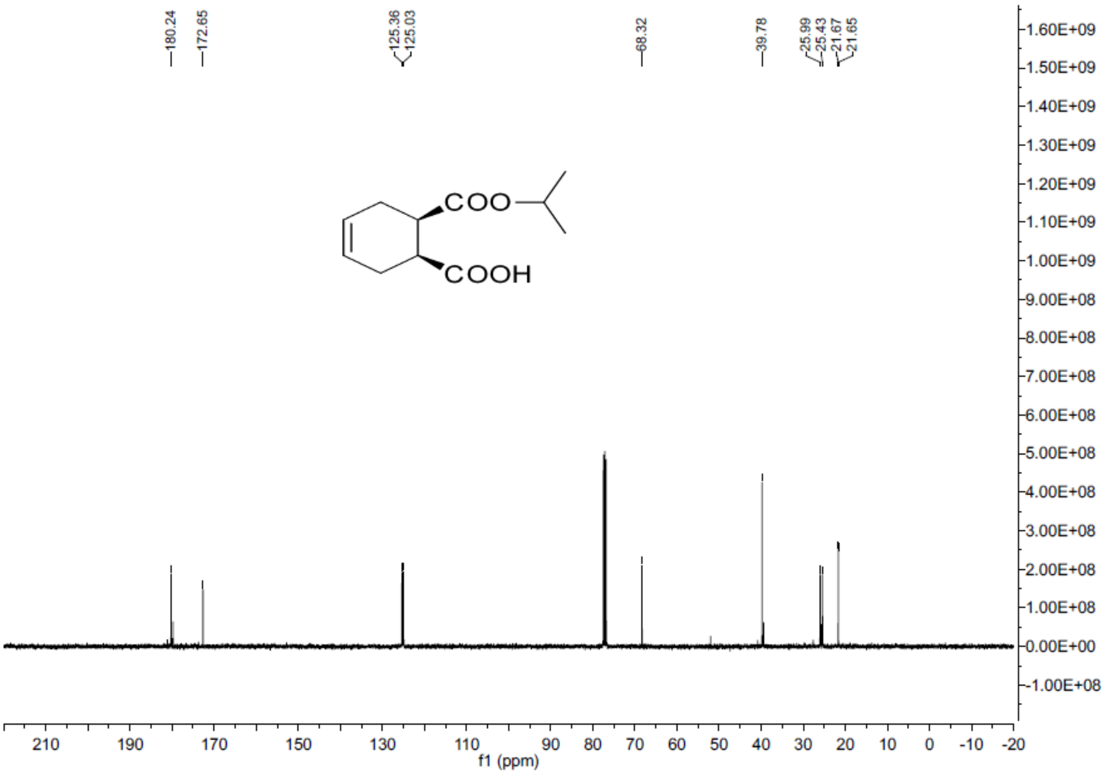

(1*S*,6*R*)-6-((Allyloxy)carbonyl)cyclohex-3-enecarboxylic acid (9o)

^{1}H NMR

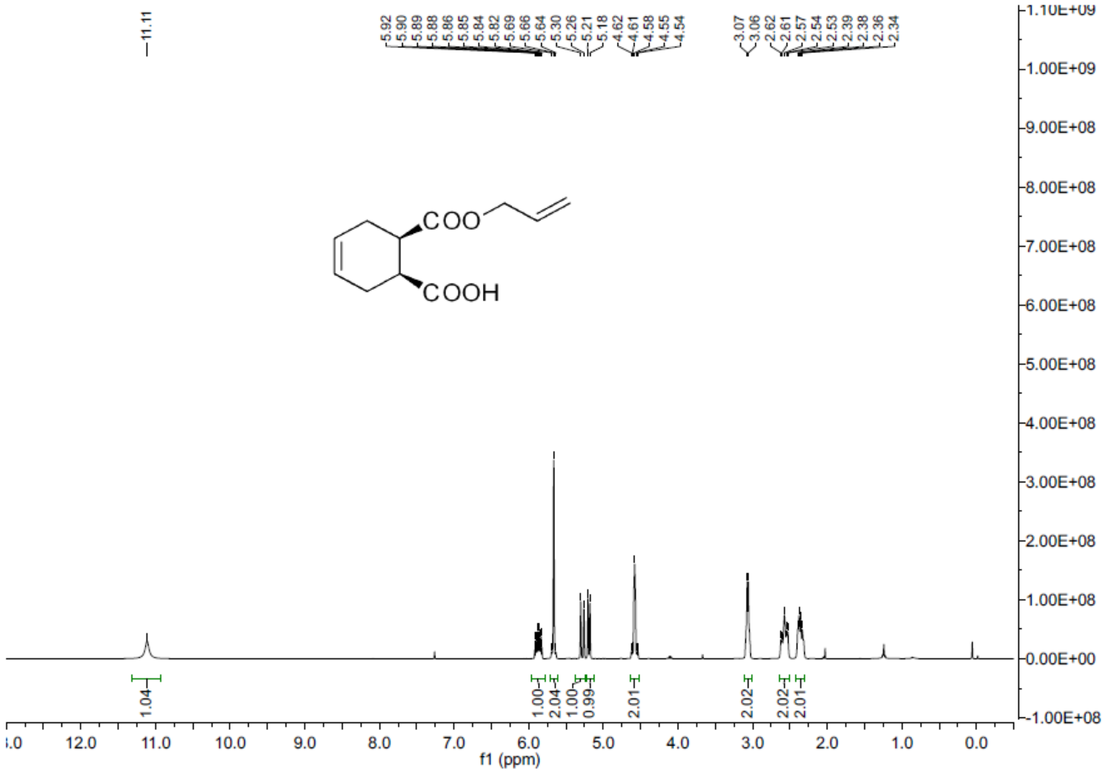

^{13}C NMR

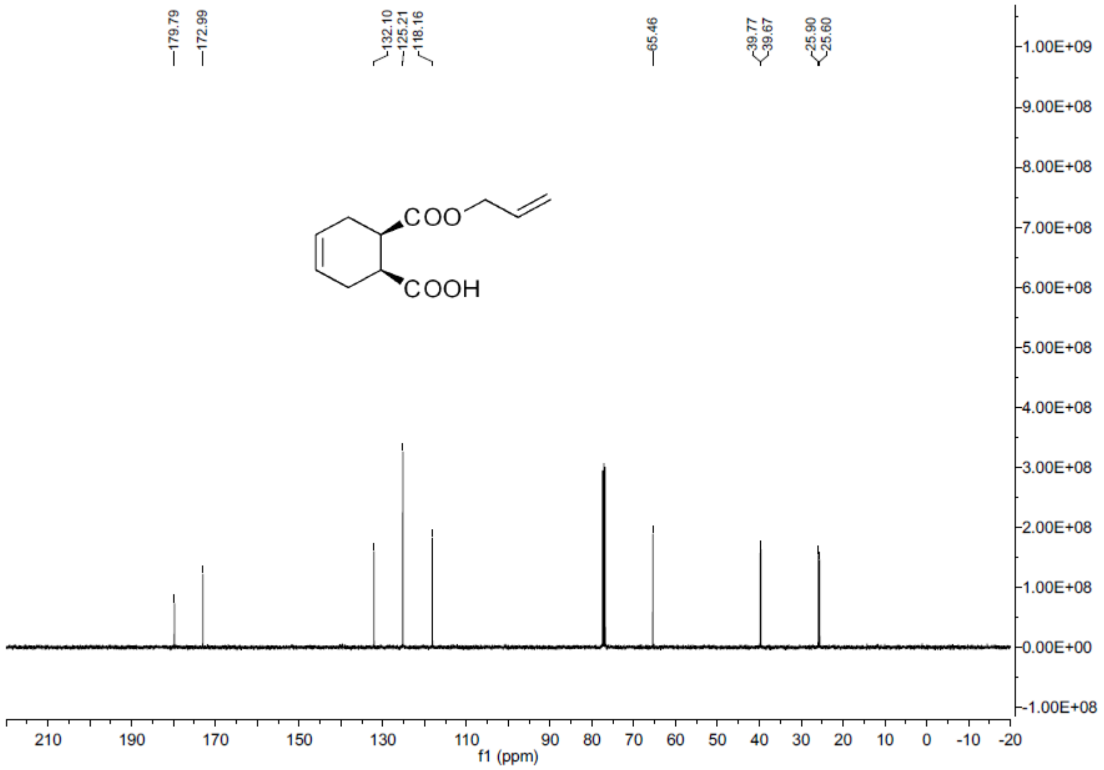

(1*S*,6*R*)-6-((Benzyloxy)carbonyl)cyclohex-3-enecarboxylic acid (9p)

^{1}H NMR

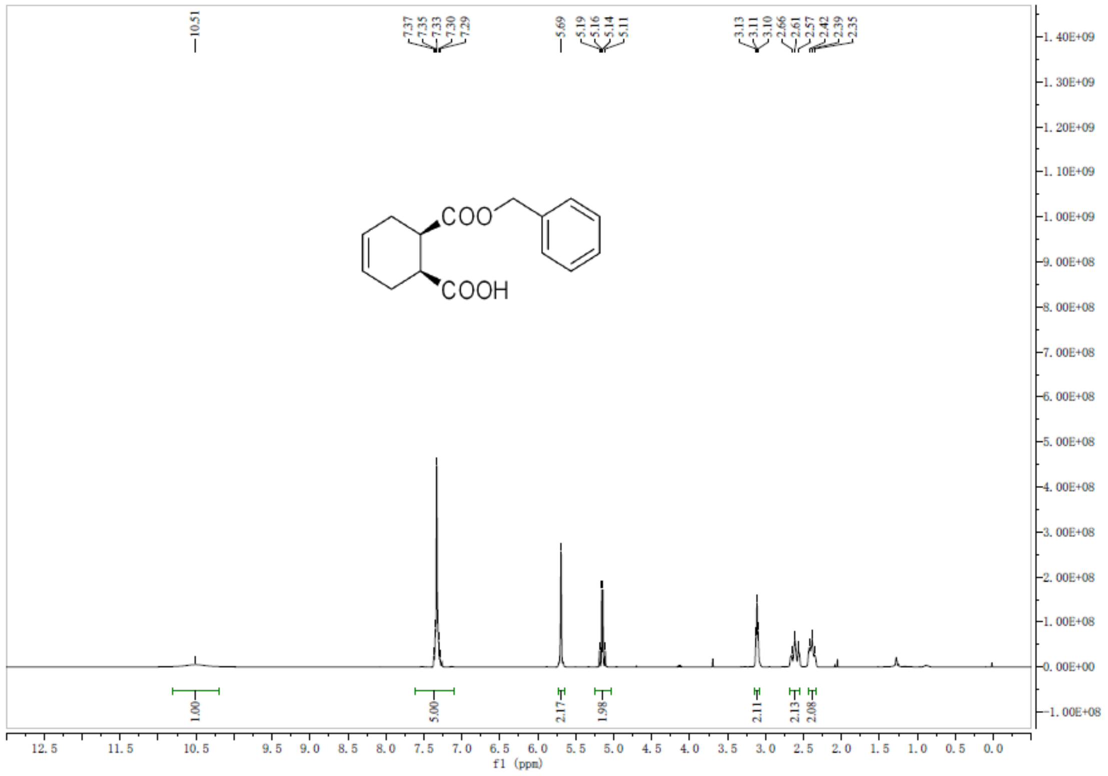

^{13}C NMR

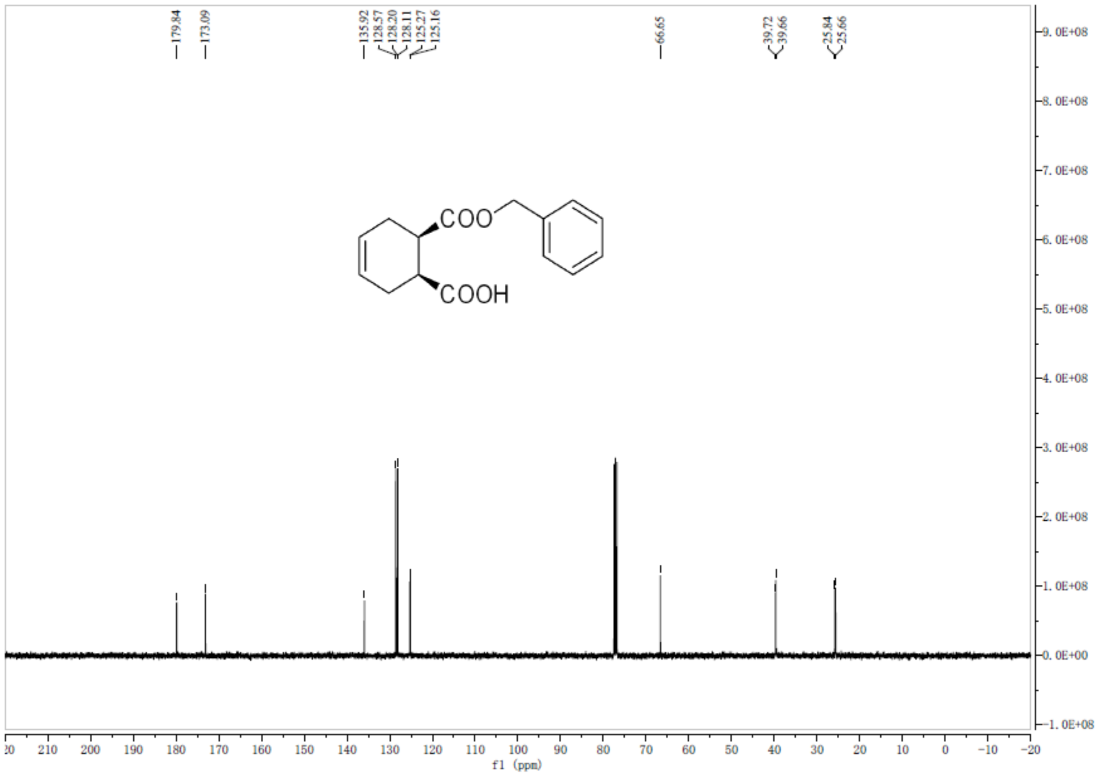

(1*S*,6*R*)-6-((Cinnamyloxy)carbonyl)cyclohex-3-enecarboxylic acid (9q)

^{1}H NMR

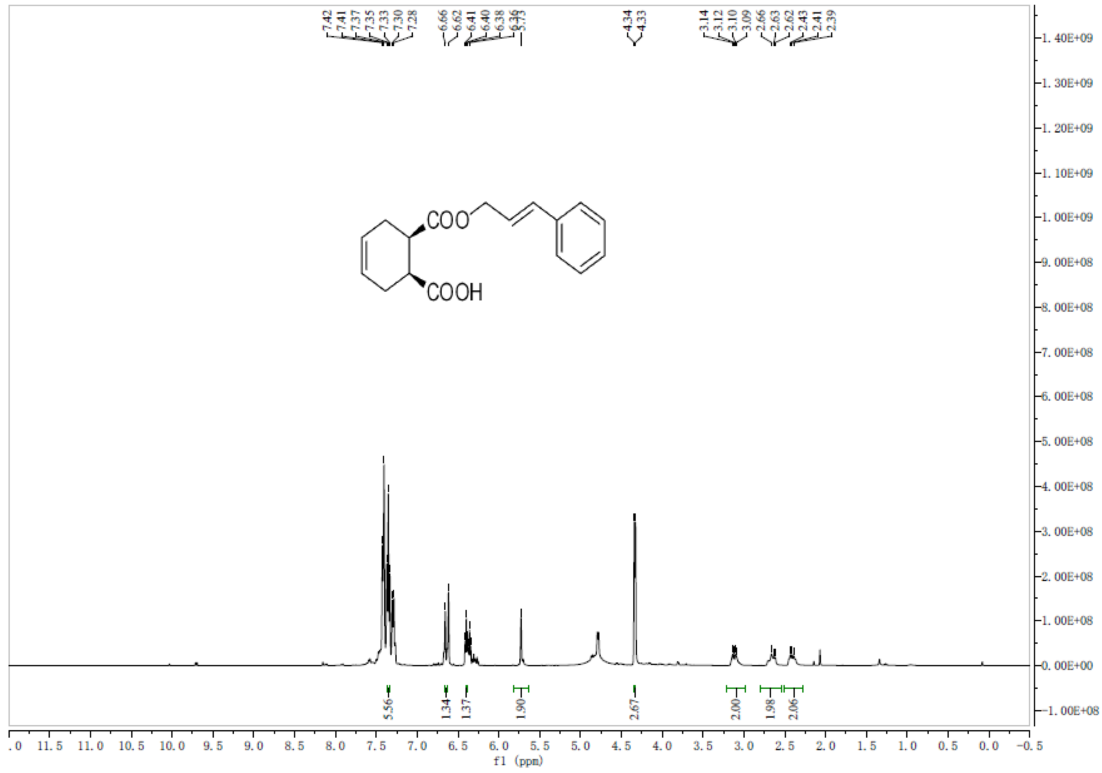

^{13}C NMR

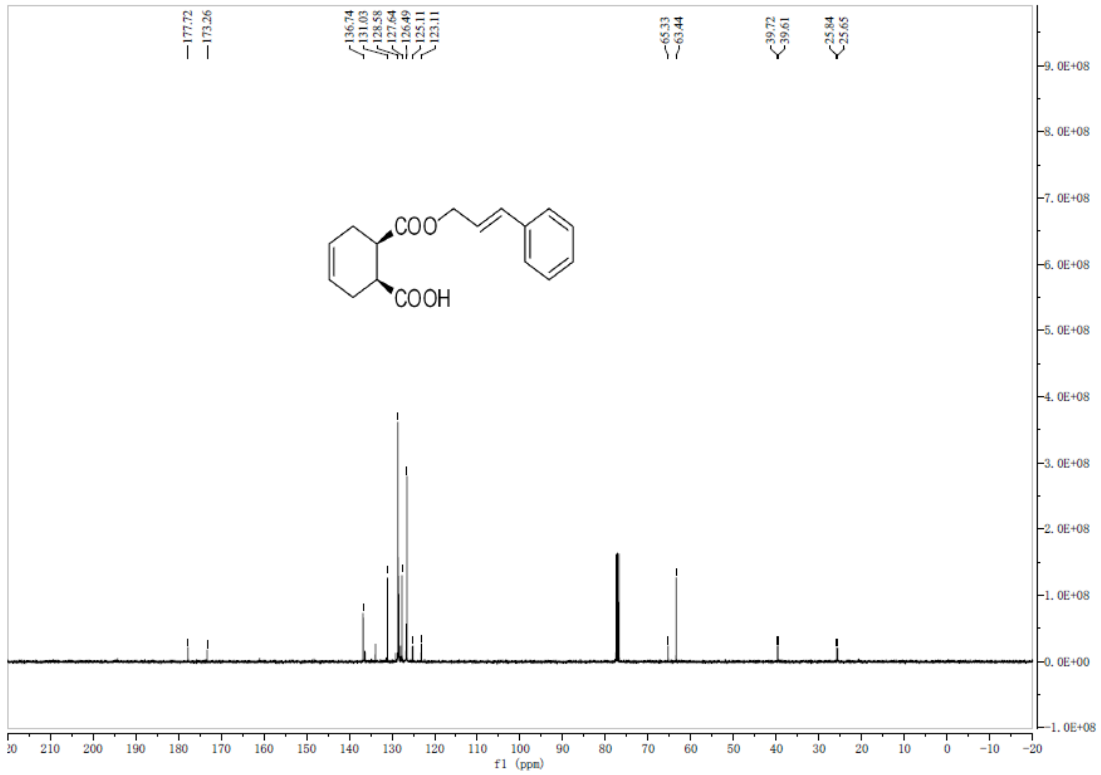

Dimethyl 3-(allyloxy)pentanedioate (14)

^{1}H NMR

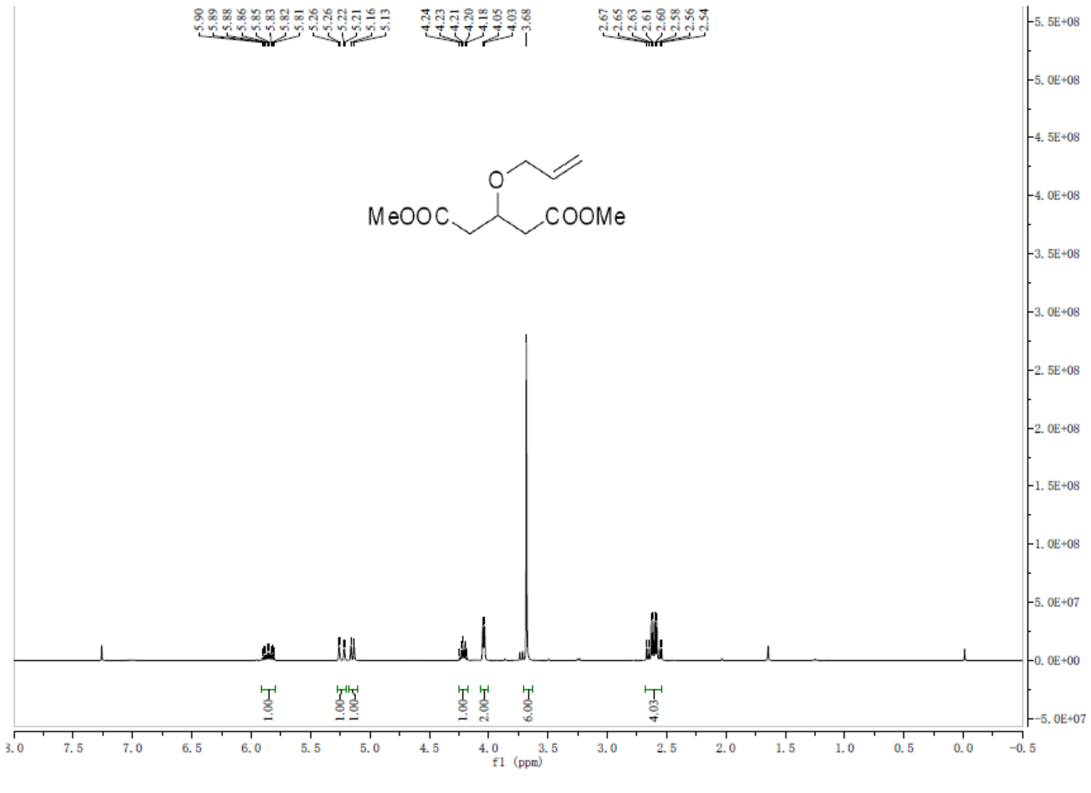

^{13}C NMR

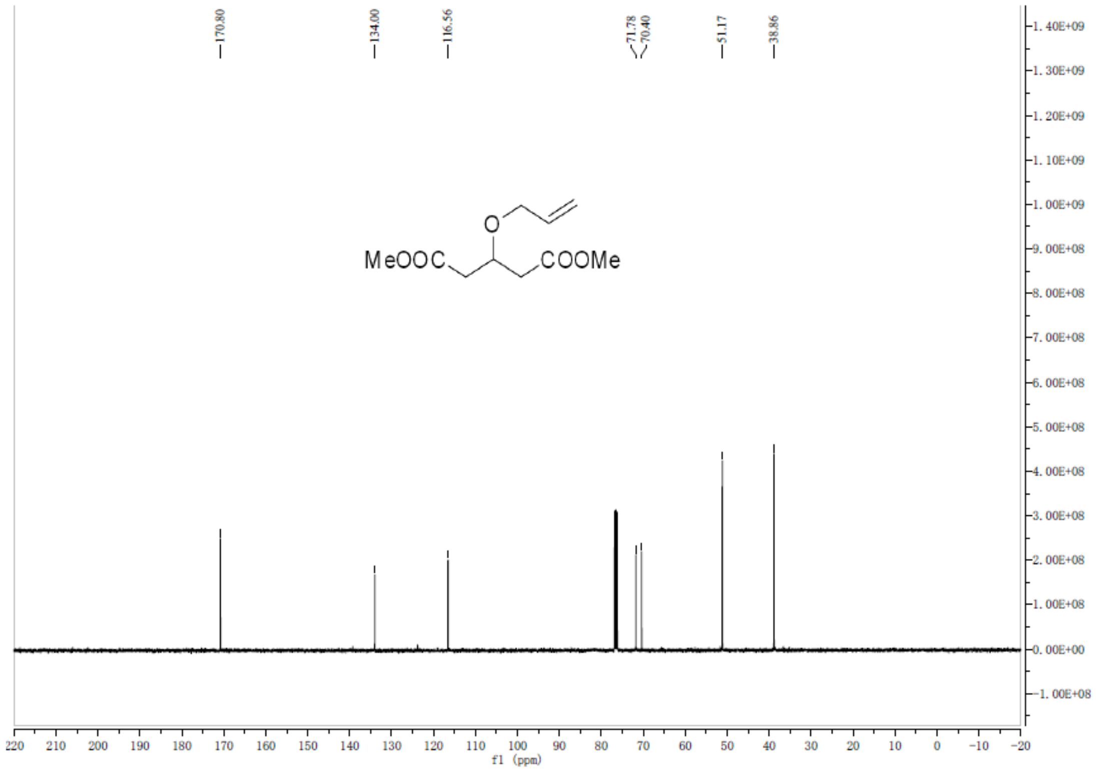
170.80
134.00
116.56
71.78
70.40
51.17
38.86
MeOOC
O
COOMe
f1 (ppm)

HRMS

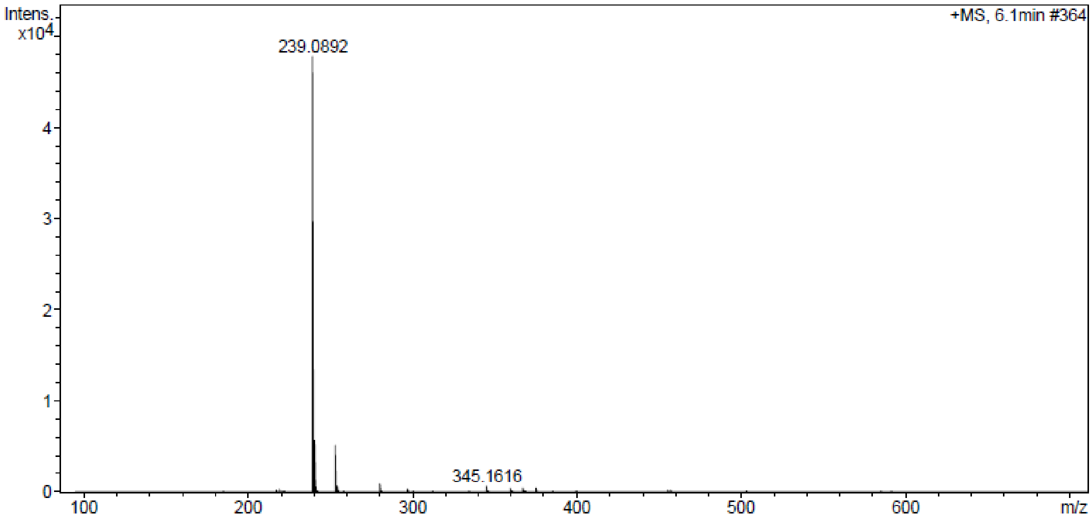
Intens.
x10⁴
+MS, 6.1min #364
239.0892
345.1616
m/z

3-(Allyloxy)pentanedioic acid (15)

^{1}H NMR

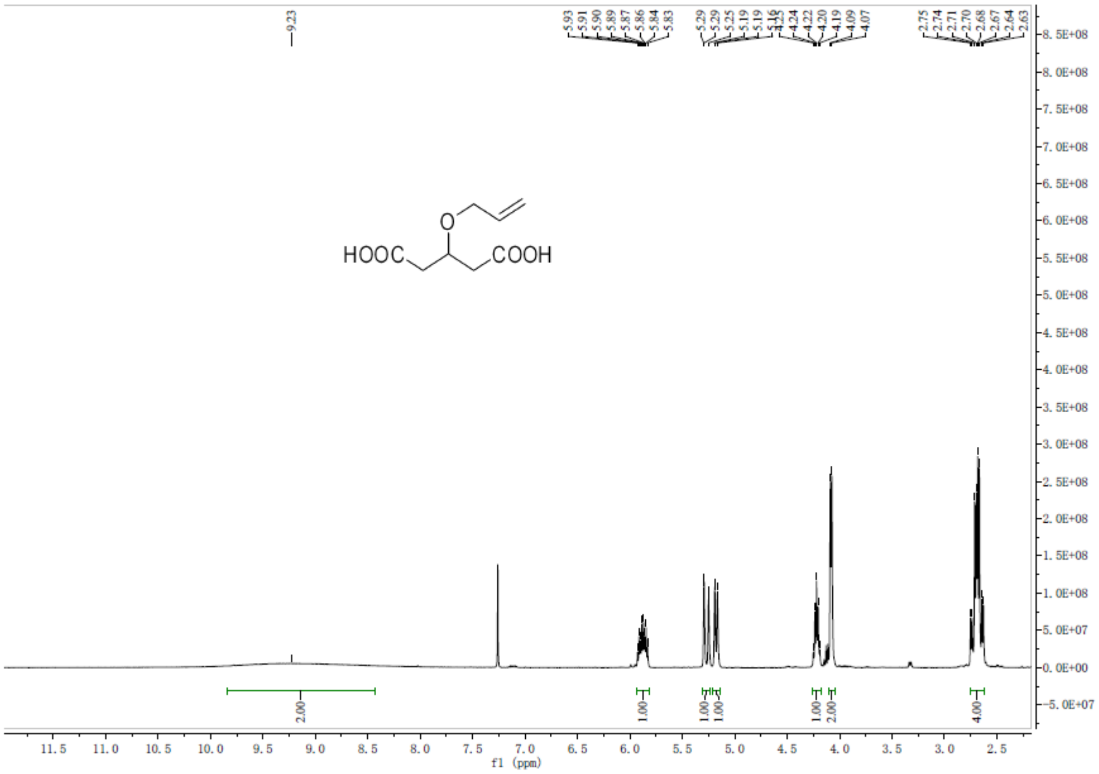

^{13}C NMR

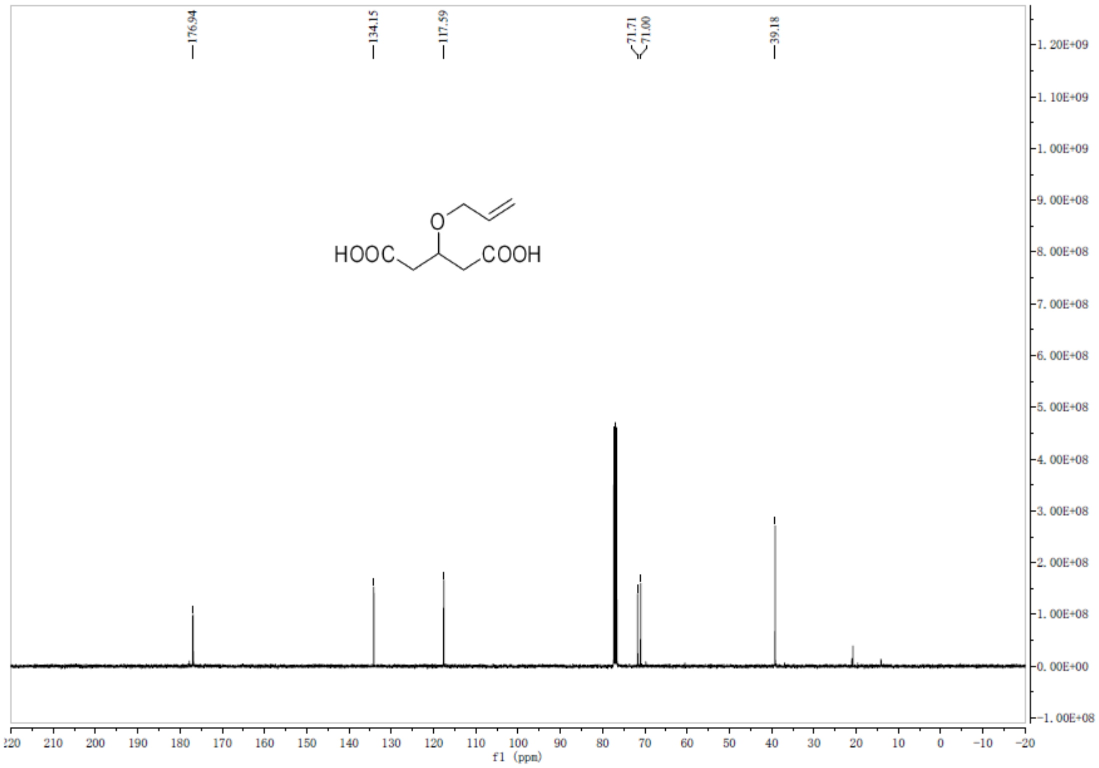

HRMS

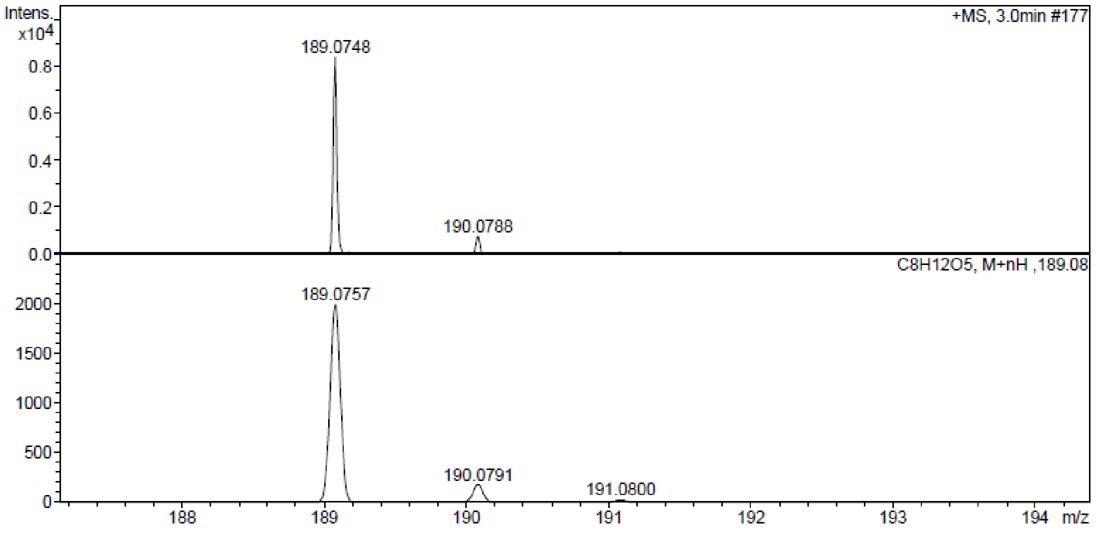

3-(Allyloxy)glutaric anhydride (8h)

^{1}H NMR

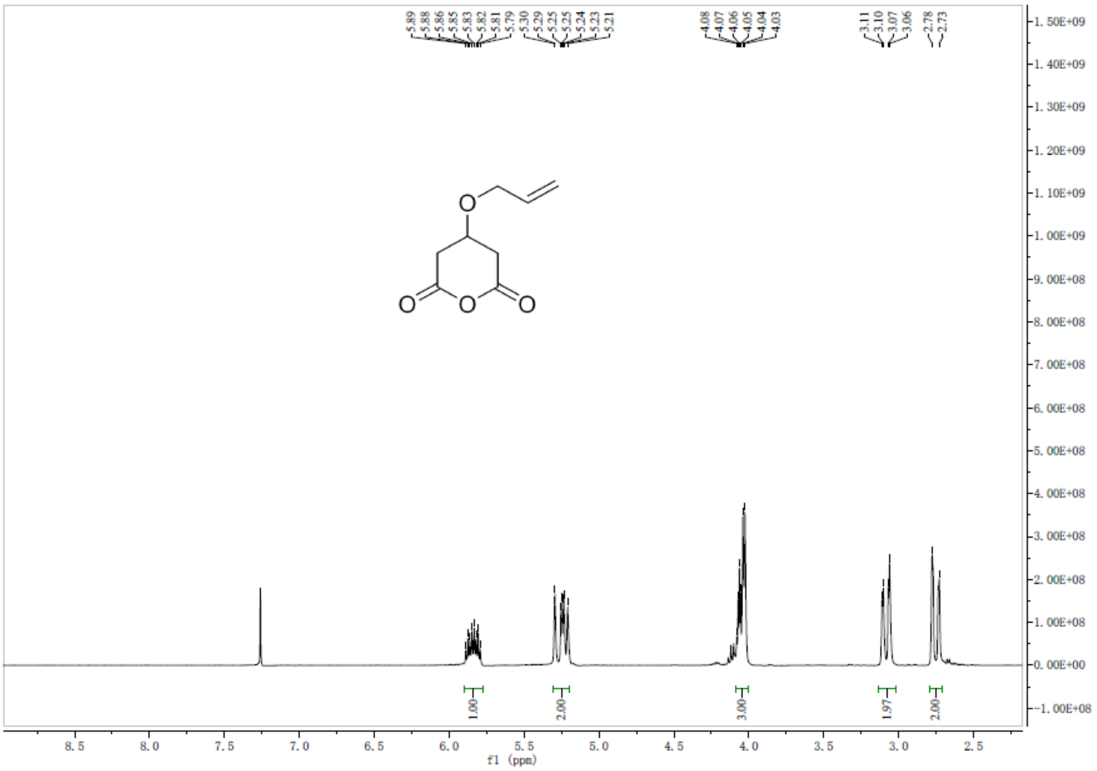

^{13}C NMR

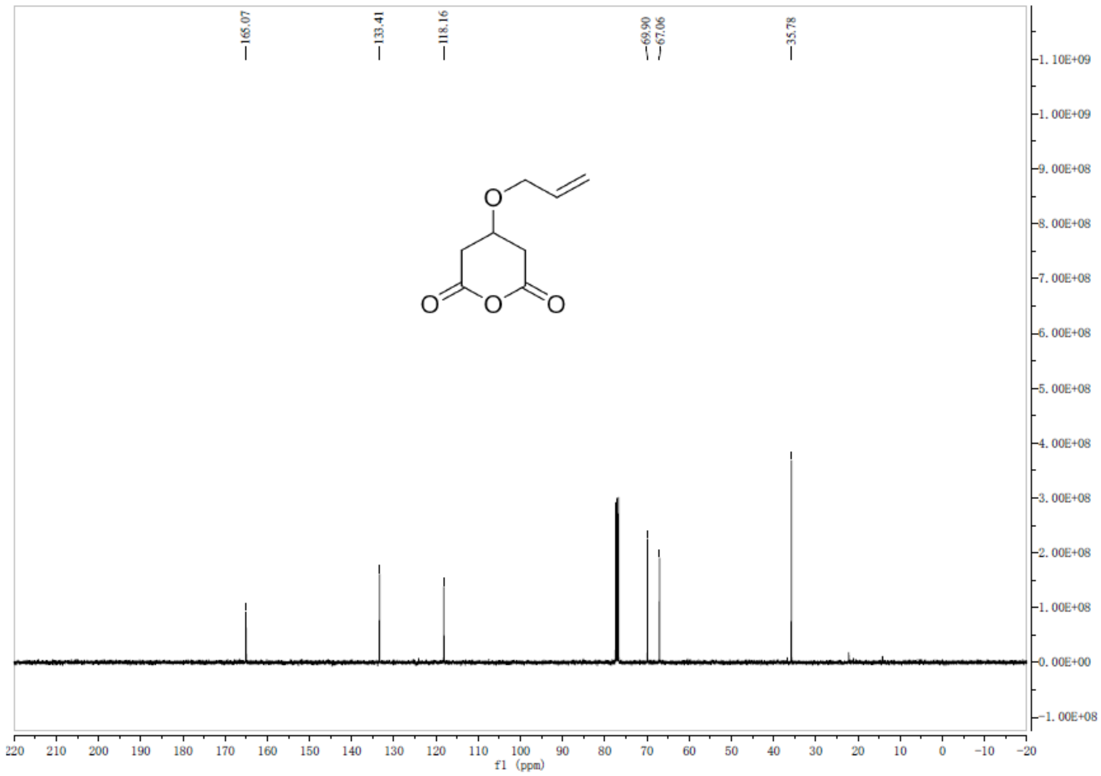

165.07
133.41
118.16
69.90
67.06
35.78
f1 (ppm)

HRMS

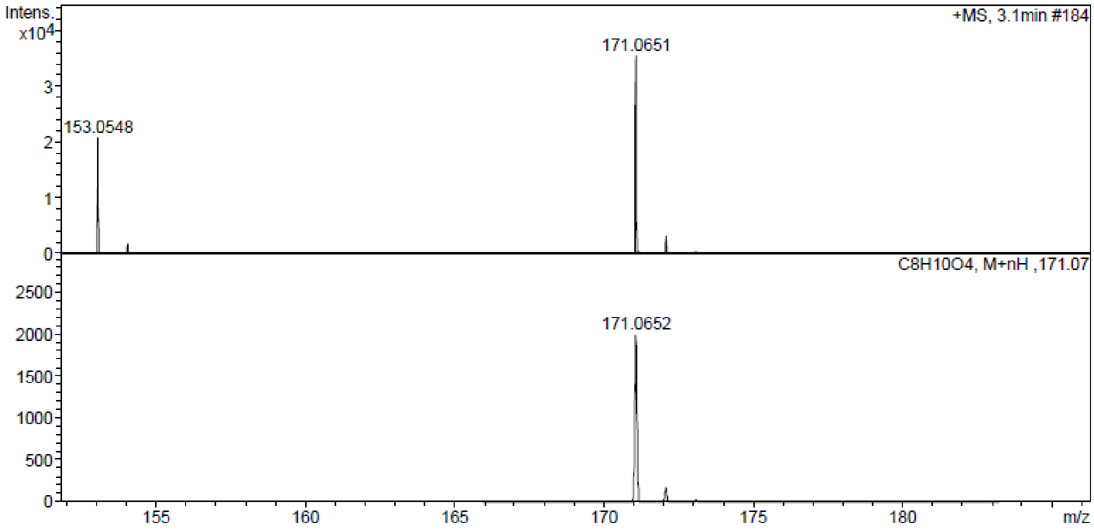

Intens.
x10^4
+MS, 3.1min #184
171.0651
153.0548
C8H10O4, M+nH ,171.07
171.0652
m/z

(*S*)-Methyl-3-(allyloxy)-4-(((benzyloxy)carbonyl)amino)butanoate (11)

^{1}H NMR

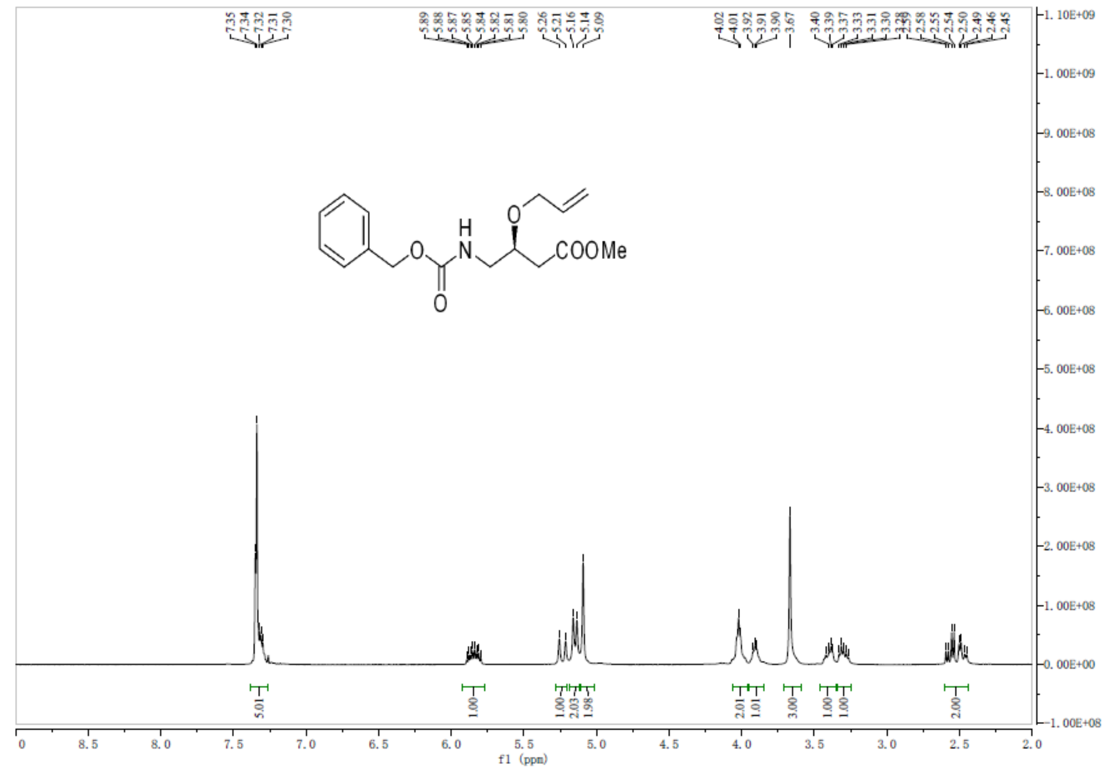

^{13}C NMR

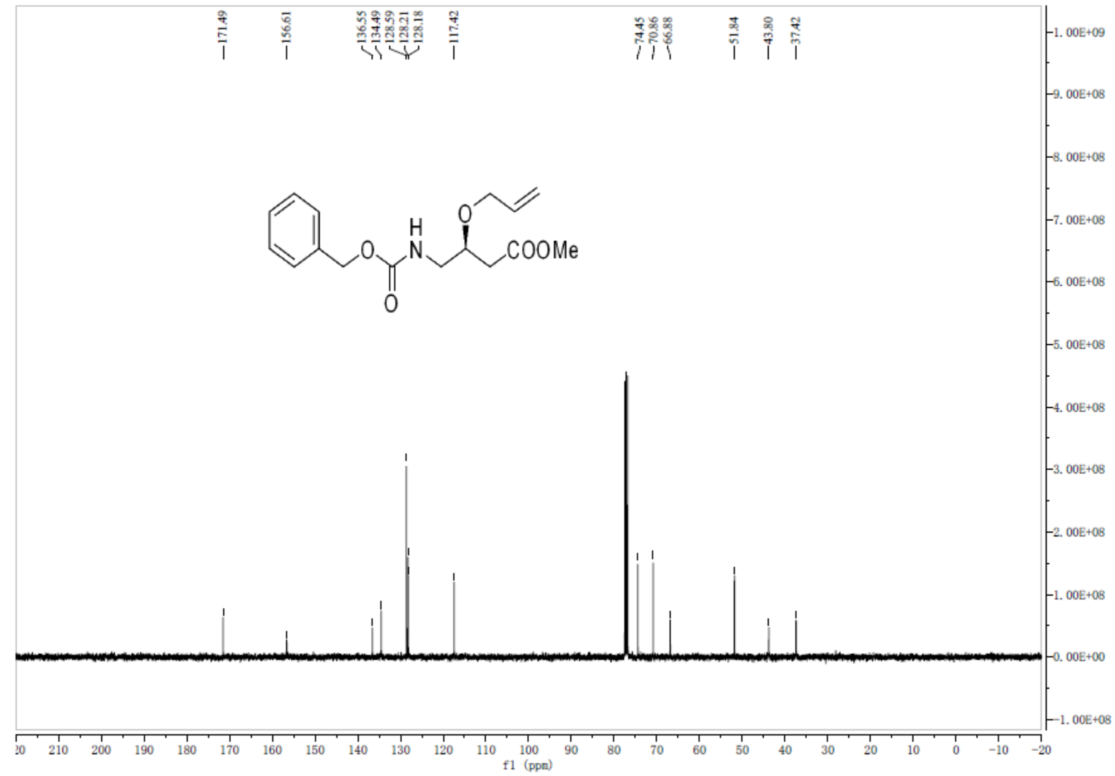

HRMS

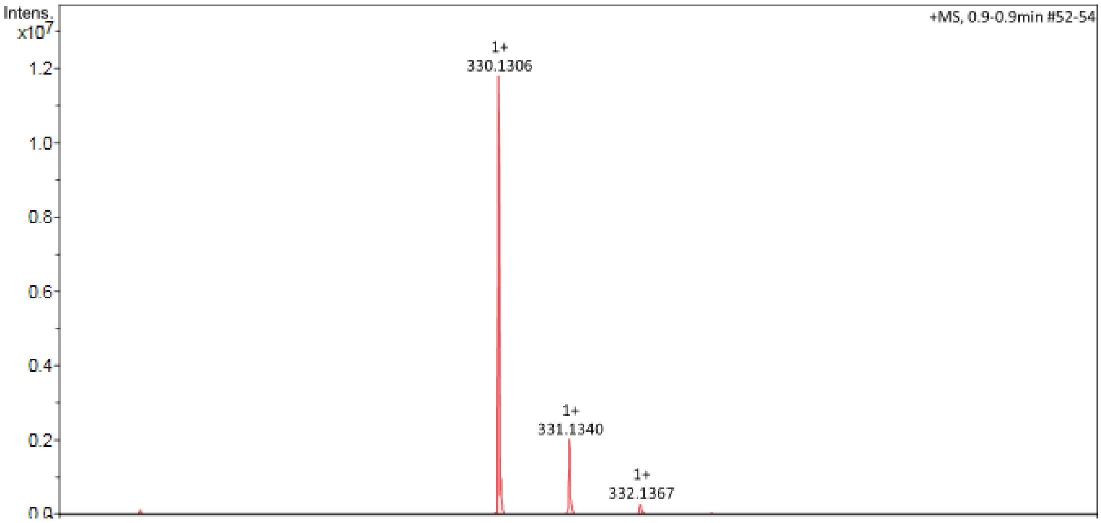

(*S*)-Methyl 4-(((benzyloxy)carbonyl)amino)-3-hydroxybutanoate (12)

^{1}H NMR

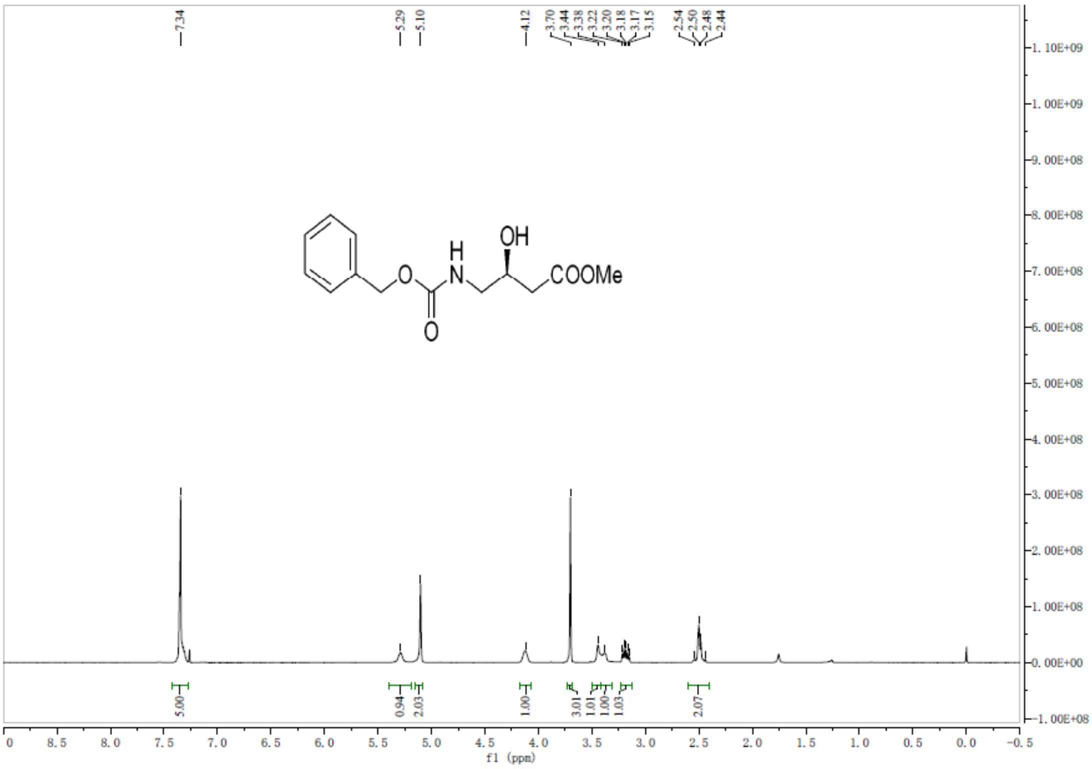

^{13}C NMR

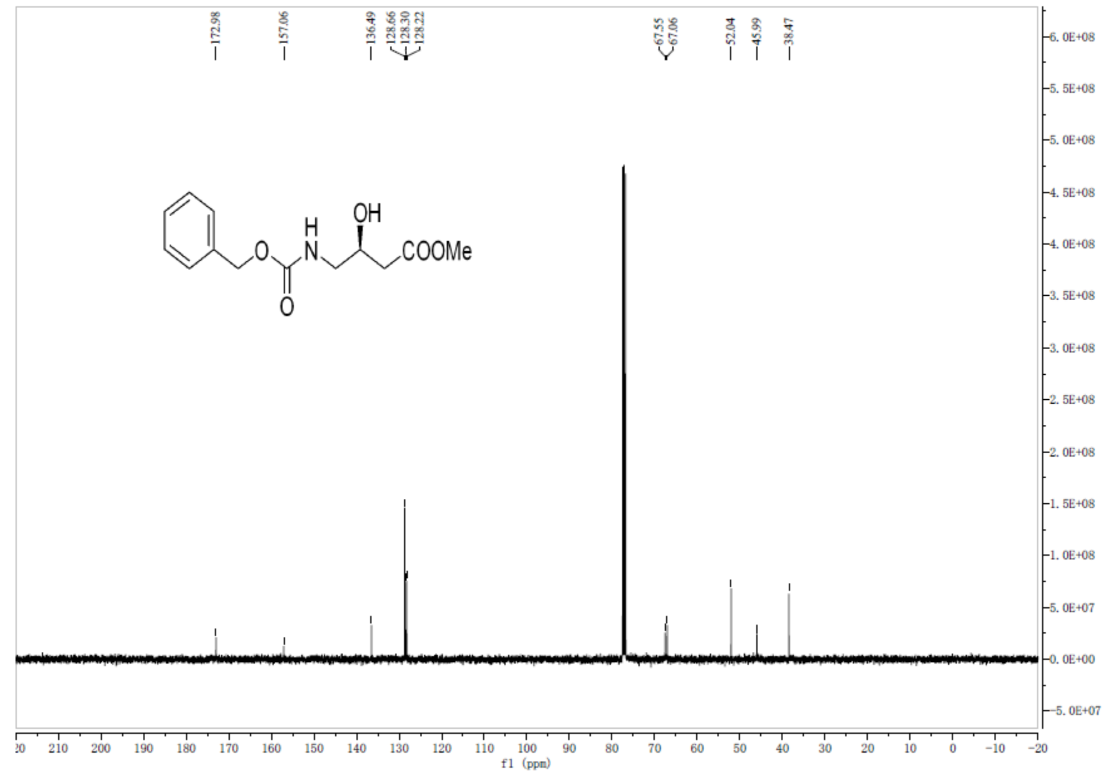

(*S*)-GABOB (13)

^{1}H NMR

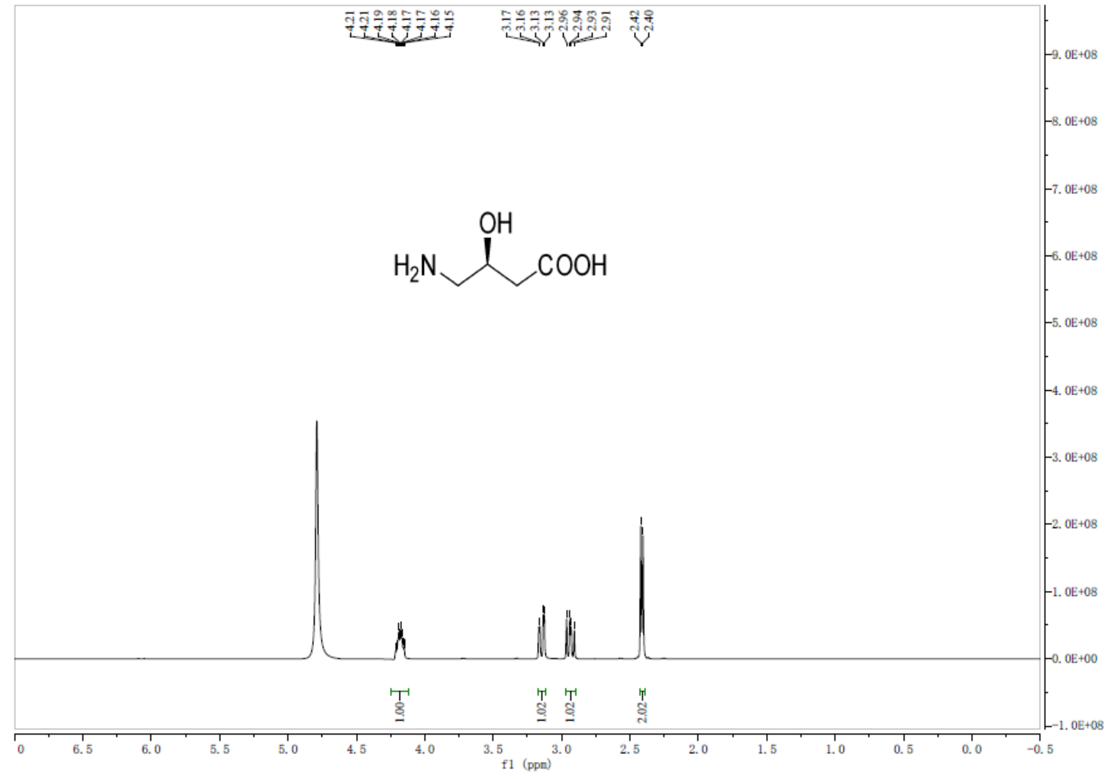

^{13}C NMR

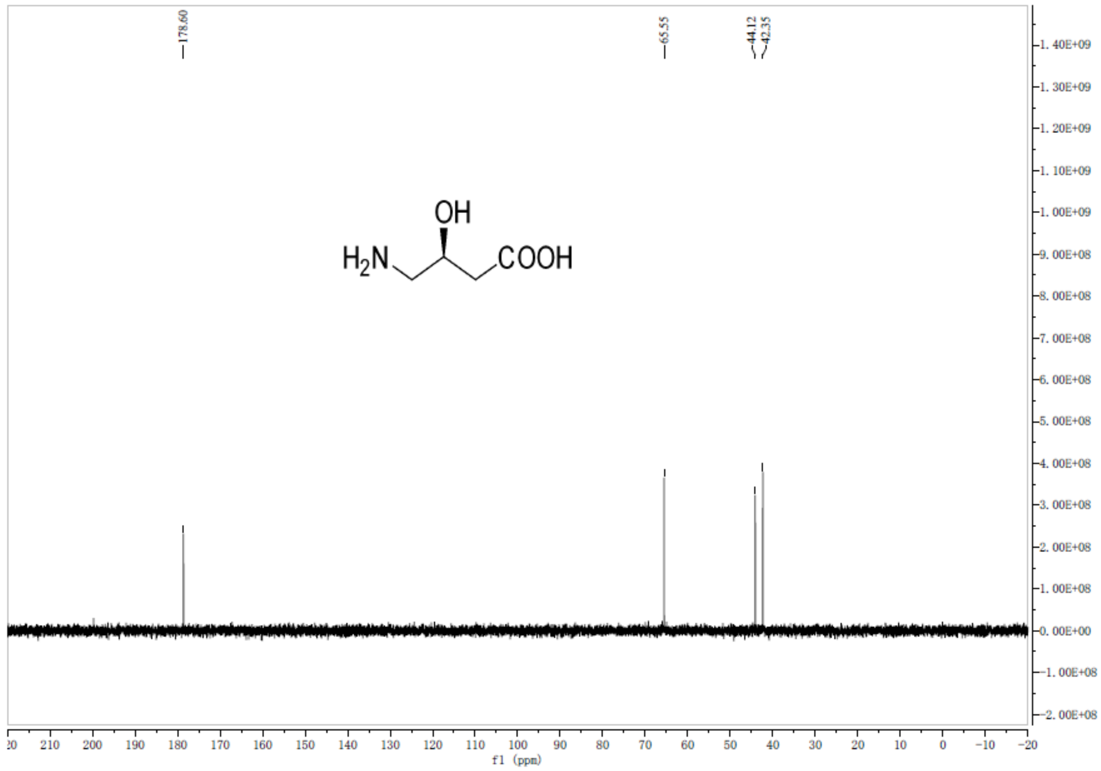

HRMS

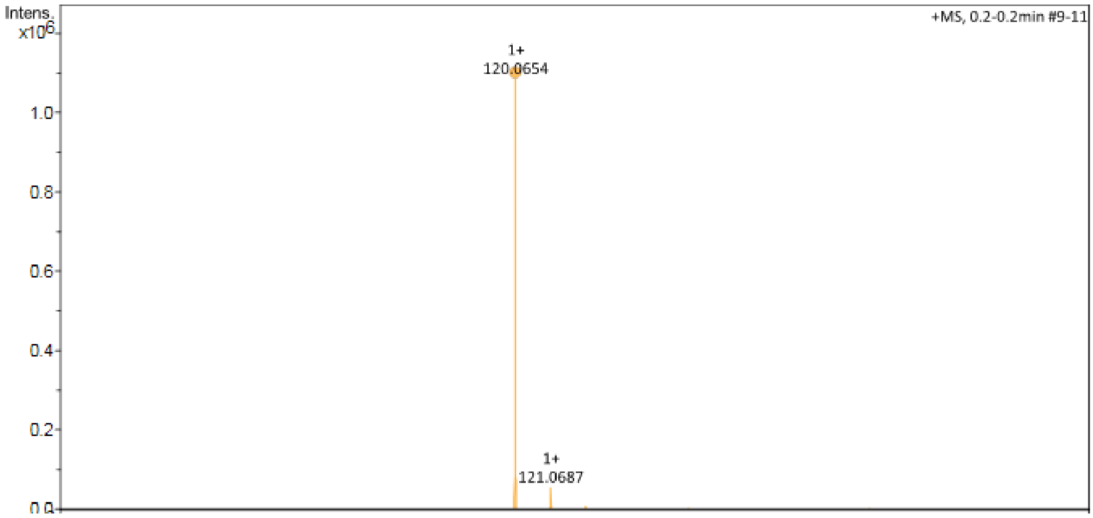

Chiral HPLC:

(1*S*,6*R*)-6-(Methoxycarbonyl)cyclohex-3-enecarboxylic acid (9a)

Chiral HPLC (Chiralcel OD-H column), Hexane/*i*-PrOH = 93/7, Flow rate: 0.5 mL/min, UV detection at 220 nm, T = 30°C, retention time: t(major) = 33.1 min, t(minor) = 45.3 min.

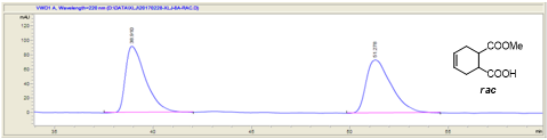

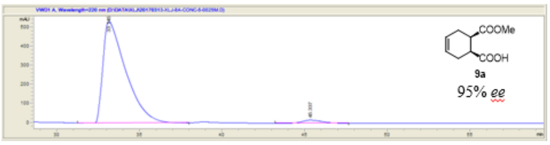

Entry	Time	Peak area	Peak height	Peak width	Symmetric factor	Peak area%
1	38.91	6532.5	92.2	1.0816	0.441	49.899
2	51.278	6558.9	73.7	1.3092	0.523	50.101

Entry	Time	Peak area	Peak height	Peak width	Symmetric factor	Peak area%
1	33.145	51055.2	533.2	1.4091	0.326	97.239
2	45.337	1449.7	16.8	1.3058	0.691	2.761

(1*S*,2*R*)-2-(Methoxycarbonyl)cyclohexanecarboxylic acid (9b)

Chiral HPLC (Chiralcel AD-H column), Hexane/*i*-PrOH = 93/7, Flow rate: 0.5 mL/min, UV detection at 220 nm, T = 30°C, retention time: t(minor) = 23.2 min, t(major) = 29.9 min.

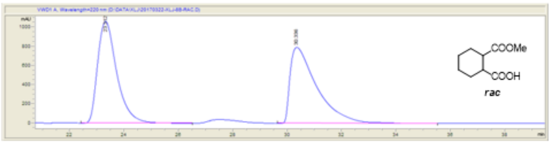

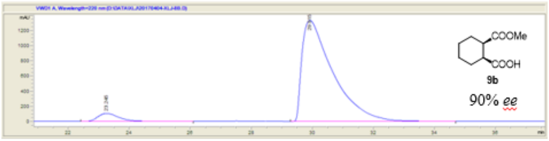

Entry	Time	Peak area	Peak height	Peak width	Symmetric factor	Peak area%
1	23.312	52213.9	1065.4	0.7538	0.651	48.956
2	30.336	54440.7	793.9	1.009	0.302	51.044

Entry	Time	Peak area	Peak height	Peak width	Symmetric factor	Peak area%
1	23.245	5106.3	105.2	0.7502	0.635	5.134
2	29.915	94362.4	1335.7	1.0212	0.313	94.866

(1*S*,2*R*,3*S*,4*R*)-3-(Methoxycarbonyl)-7-oxabicyclo[2.2.1]hept-5-ene-2-carboxylic acid (9c)

Chiral HPLC (Chiralcel OD-H column), Hexane/*i*-PrOH = 85/15, Flow rate: 0.5 mL/min, UV detection at 220 nm, T = 30°C, retention time: t(major) = 23.8 min, t(minor) = 37.8 min.

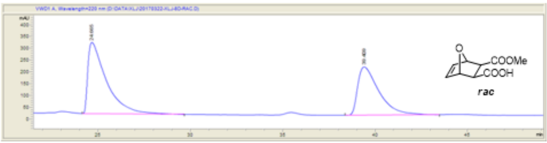

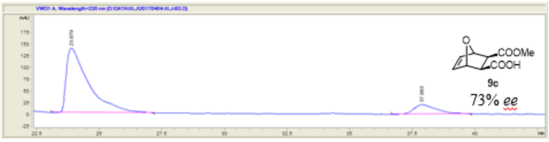

Entry	Time	Peak area	Peak height	Peak width	Symmetric factor	Peak area%
1	24.655	21192.5	301.3	1.0076	0.254	56.593
2	39.409	16254.5	203.6	1.1914	0.401	43.407

Entry	Time	Peak area	Peak height	Peak width	Symmetric factor	Peak area%
1	23.879	8489.2	137.3	0.8673	0.267	86.657
2	37.883	1307.2	20.4	0.9131	0.565	13.343

(1*R*,2*S*,3*R*,4*S*)-3-(Methoxycarbonyl)bicyclo[2.2.1]hept-5-ene-2-carboxylic acid (9d)

Chiral HPLC (Chiralcel AD-H column), Hexane/*i*-PrOH = 96/4, Flow rate: 0.5 mL/min, UV detection at 220 nm, T = 30°C, retention time: t(minor) = 47.8 min, t(major) = 50.7 min.

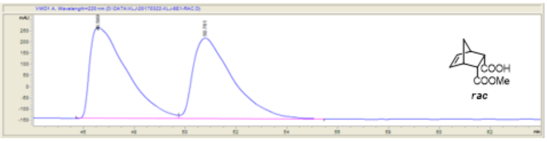

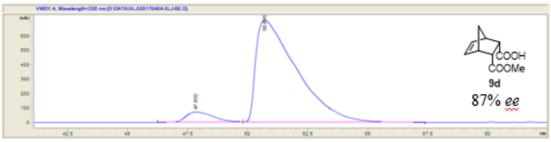

Entry	Time	Peak area	Peak height	Peak width	Symmetric factor	Peak area%	Entry	Time	Peak area	Peak height	Peak width	Symmetric factor	Peak area%
1	46.569	40886.6	406.5	1.4644	0.265	51.193	1	47.832	5769.2	71.4	1.275	0.545	6.456
2	50.781	38981.2	360.2	1.5524	0.416	48.807	2	50.695	83595.6	704.6	1.6271	0.257	93.544

(*R*)-5-Methoxy-3-methyl-5-oxopentanoic acid (9e)

Chiral HPLC (Chiralcel AD-H column), Hexane/*i*-PrOH = 96/4, Flow rate: 0.5 mL/min, UV detection at 220 nm, T = 30°C, retention time: t(minor) = 38.3 min, t(major) = 44.1 min.

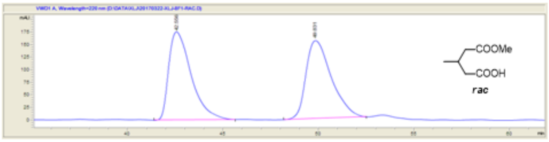

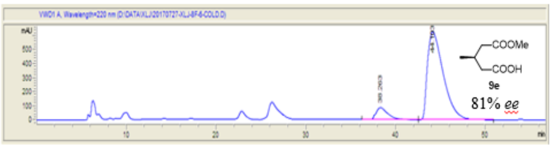

Entry	Time	Peak area	Peak height	Peak width	Symmetric factor	Peak area%	Entry	Time	Peak area	Peak height	Peak width	Symmetric factor	Peak area%
1	42.556	14359.8	176	1.2731	0.5	49.920	1	38.263	8261.4	88.7	1.3905	0.512	9.587
2	49.831	14405.7	155.6	1.3534	0.562	50.080	2	44.103	77907.3	635.3	1.8827	0.465	90.413

(*R*)-3-Ethyl-5-methoxy-5-oxopentanoic acid (9f)

Chiral HPLC (Chiralcel AD-H column), Hexane/*i*-PrOH = 94/6, Flow rate: 0.5 mL/min, UV detection at 220 nm, T = 30°C, retention time: t(minor) = 26.7 min, t(major) = 30.3 min.

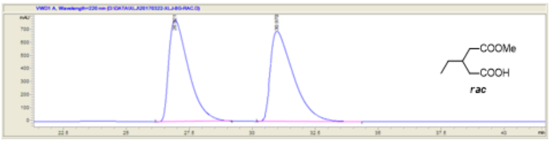

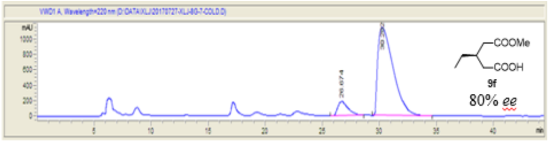

Entry	Time	Peak area	Peak height	Peak width	Symmetric factor	Peak area%	Entry	Time	Peak area	Peak height	Peak width	Symmetric factor	Peak area%
1	26.921	42630.3	779.7	0.8209	0.423	49.262	1	26.674	11525.9	184.9	0.9433	0.538	9.690
2	30.97	43908.2	692.6	0.9216	0.372	50.738	2	30.282	107424.3	1140	1.4514	0.374	90.310

(*R*)-3-Isopropyl-5-methoxy-5-oxopentanoic acid (9g)

Chiral HPLC (Chiralcel IA-H column), Hexane/*i*-PrOH = 94/6, Flow rate: 0.5 mL/min, UV detection at 220 nm, T = 30°C, retention time: t(minor) = 24.4 min, t(major) = 31.0 min.

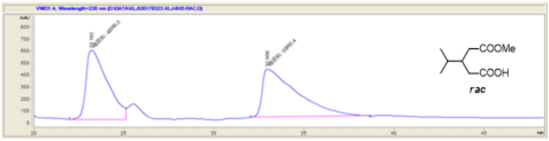

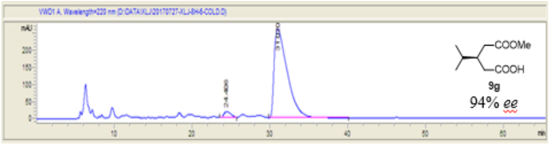

Entry	Time	Peak area	Peak height	Peak width	Symmetric factor	Peak area%	Entry	Time	Peak area	Peak height	Peak width	Symmetric factor	Peak area%
1	23.192	48390.3	579	1.3929	0.349	47.324	1	24.406	963.8	17.1	0.8791	0.567	3.006
2	32.956	53862.4	402.2	2.2321	0.177	52.676	2	31.02	31101.6	263.7	1.72	0.347	96.994

(*R*)-3-(Allyloxy)-5-methoxy-5-oxopentanoic acid (9h)

Chiral HPLC (Chiralcel AD-H column), Hexane/*i*-PrOH = 95/5, Flow rate: 0.6 mL/min, UV detection at 220 nm, T = 30°C, retention time: t(minor) = 50.4 min, t(major) = 58.8 min.

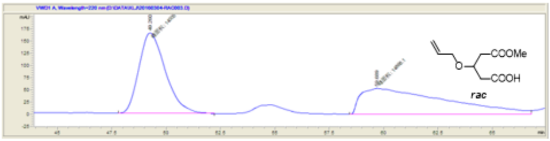

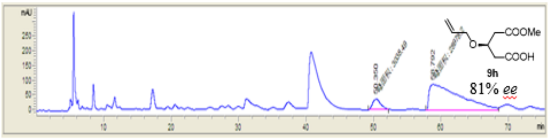

Entry	Time	Peak area	Peak height	Peak width	Symmetric factor	Peak area%
1	49.26	14005	165	1.4149	0.701	48.505
2	59.689	14868.1	53.7	4.6156	0.23	51.495

Entry	Time	Peak area	Peak height	Peak width	Symmetric factor	Peak area%
1	50.35	3038.5	37.2	1.3626	0.709	9.490
2	58.792	28978.2	88.6	5.451	0.131	90.510

(*R*)-5-Methoxy-5-oxo-3-phenylpentanoic acid (9i)

Chiral HPLC (Chiralcel AD-H column), Hexane/*i*-PrOH = 90/10, Flow rate: 0.5 mL/min, UV detection at 220 nm, T = 30°C, retention time: t(minor) = 43.2 min, t(major) = 49.2 min.

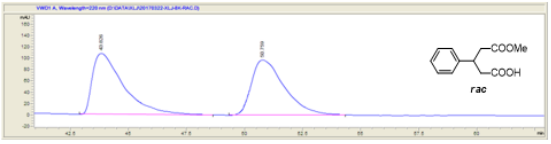

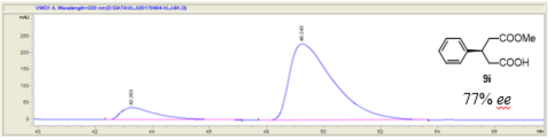

Entry	Time	Peak area	Peak height	Peak width	Symmetric factor	Peak area%
1	43.826	10090.4	108.1	1.3476	0.367	51.235
2	50.759	9603.9	97.6	1.4637	0.481	48.765

Entry	Time	Peak area	Peak height	Peak width	Symmetric factor	Peak area%
1	43.263	3143.3	36.4	1.2708	0.391	11.420
2	49.243	24380.3	228.7	1.6055	0.376	88.580

(*R*)-3-(4-Chlorophenyl)-5-methoxy-5-oxopentanoic acid (9j)

Chiral HPLC (Chiralcel OD-H column), Hexane/*i*-PrOH = 92/8, Flow rate: 0.5 mL/min, UV detection at 220 nm, T = 30°C, retention time: t(minor) = 47.7 min, t(major) = 53.3 min.

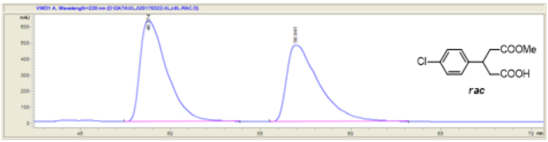

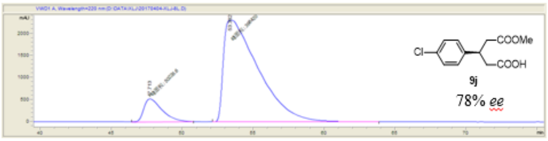

Entry	Time	Peak area	Peak height	Peak width	Symmetric factor	Peak area%
1	48.774	64414.3	629	1.5588	0.419	51.982
2	56.941	59502.5	474.5	1.787	0.358	48.018

Entry	Time	Peak area	Peak height	Peak width	Symmetric factor	Peak area%
1	47.713	50226.6	535.1	1.5644	0.474	11.195
2	53.382	398419.8	2307.4	2.8779	0.235	88.805

(*R*)-5-Methoxy-5-oxo-3-(*p*-tolyl)pentanoic acid (9k)

Chiral HPLC (Chiralcel IA-H column), Hexane/*i*-PrOH = 95/5, Flow rate: 1.0 mL/min, UV detection at 220 nm, T = 30°C, retention time: t(minor) = 41.1 min, t(major) = 47.8 min.

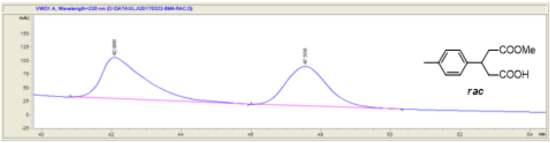

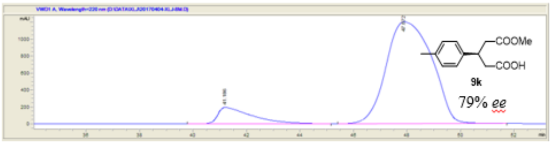

Entry	Time	Peak area	Peak height	Peak width	Symmetric factor	Peak area%
1	42.088	6702.8	75.9	1.2607	0.404	52.517
2	47.558	6060.2	72.7	1.3091	0.832	47.483

Entry	Time	Peak area	Peak height	Peak width	Symmetric factor	Peak area%
1	41.186	17155.6	193.4	1.2798	0.259	10.685
2	47.872	143428	1207.9	1.6013	0.632	89.315

(*R*)-5-Methoxy-3-(4-methoxyphenyl)-5-oxopentanoic acid (9l)

Chiral HPLC (Chiralcel AD-H column), Hexane/*i*-PrOH = 93/7, Flow rate: 0.6 mL/min, UV detection at 220 nm, T = 30°C, retention time: t(minor) = 87.91 min, t(major) = 103.9 min.

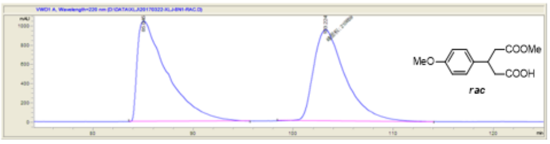

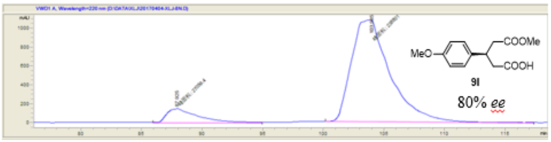

Entry	Time	Peak area	Peak height	Peak width	Symmetric factor	Peak area%
1	85.045	215612.3	1038.6	2.829	0.245	50.581
2	103.224	210659.3	946.2	3.7107	0.577	49.419

Entry	Time	Peak area	Peak height	Peak width	Symmetric factor	Peak area%
1	87.925	27059.4	149	3.0272	0.469	10.176
2	103.935	238801.3	1072.6	3.7106	0.963	89.822

(1*S*,6*R*)-6-(Ethoxycarbonyl)cyclohex-3-enecarboxylic acid (9m)

Chiral HPLC (Chiralcel IA-H column), Hexane/*i*-PrOH = 94/6, Flow rate: 0.5 mL/min, UV detection at 220 nm, T = 30°C, retention time: t(minor) = 25.7 min, t(major) = 29.5 min.

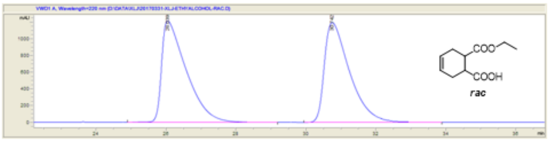

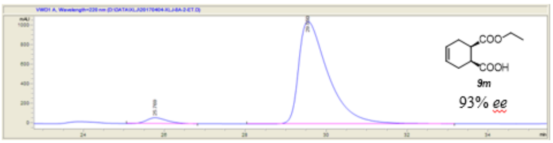

Entry	Time	Peak area	Peak height	Peak width	Symmetric factor	Peak area%
1	26.039	59988.7	1226.1	0.7162	0.308	49.876
2	30.742	60287.5	1201.5	0.7554	0.422	50.124

Entry	Time	Peak area	Peak height	Peak width	Symmetric factor	Peak area%
1	25.769	2073.5	59.1	0.5384	0.693	3.776
2	29.55	52839.6	1047.8	0.7543	0.425	96.224

(1*S*,6*R*)-6-(Isopropoxycarbonyl)cyclohex-3-enecarboxylic acid (9n)

Chiral HPLC (Chiralcel IA-H column), Hexane/*i*-PrOH = 94/6, Flow rate: 0.5 mL/min, UV detection at 220 nm, T = 30°C, retention time: t(minor) = 30.4 min, t(major) = 36.1 min.

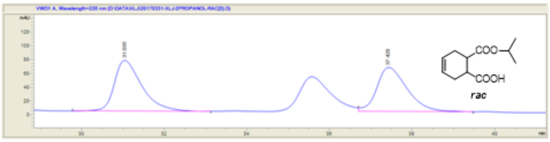

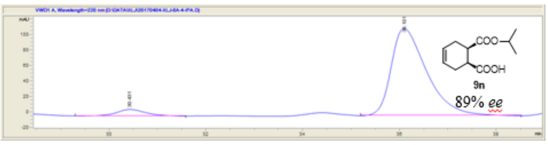

Entry	Time	Peak area	Peak height	Peak width	Symmetric factor	Peak area%
1	31.035	3401.2	73.5	0.6956	0.569	50.842
2	37.42	3288.6	64.2	0.7824	0.622	49.158

Entry	Time	Peak area	Peak height	Peak width	Symmetric factor	Peak area%
1	30.431	351.5	8.4	0.6174	0.826	5.766
2	36.101	5744.2	112.6	0.7721	0.538	94.234

(1*S*,6*R*)-6-((Allyloxy)carbonyl)cyclohex-3-enecarboxylic acid (9o)

Chiral HPLC (Chiralcel IA-H column), Hexane/*i*-PrOH = 94/6, Flow rate: 0.5 mL/min, UV detection at 220 nm, T = 30°C, retention time: t(minor) = 30.3 min, t(major) = 36.2 min.

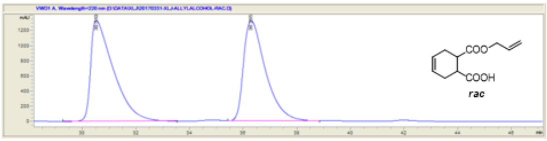

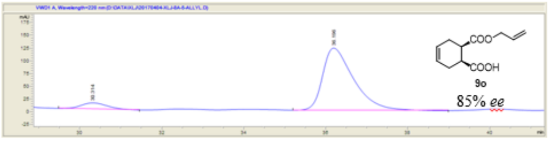

Entry	Time	Peak area	Peak height	Peak width	Symmetric factor	Peak area%
1	30.51	78338.6	1336.4	0.8559	0.3	50.991
2	36.285	75294.9	1321.7	0.8626	0.495	49.009

Entry	Time	Peak area	Peak height	Peak width	Symmetric factor	Peak area%
1	30.314	539.9	12	0.7029	0.779	7.709
2	36.196	6463.7	122.7	0.7786	0.52	92.291

(1*S*,6*R*)-6-((Benzyloxy)carbonyl)cyclohex-3-enecarboxylic acid (9p)

Chiral HPLC (Chiralcel AD-H column), Hexane/*i*-PrOH = 95/5, Flow rate: 0.2 mL/min, UV detection at 210 nm, T = 30°C, retention time: t(major) = 69.6 min, t(minor) = 75.6 min.

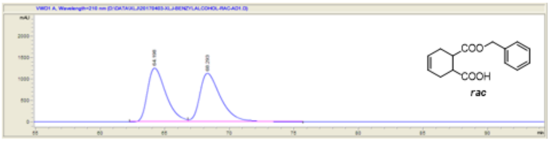

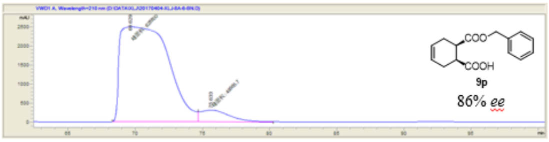

Entry	Time	Peak area	Peak height	Peak width	Symmetric factor	Peak area%
1	64.198	124487.3	1252.9	1.4882	0.588	49.338
2	68.293	127826.7	1132.9	1.5914	0.549	50.662

Entry	Time	Peak area	Peak height	Peak width	Symmetric factor	Peak area%
1	69.629	628799.9	2495.1	4.2003	0.277	92.693
2	75.633	49565.7	316.5	2.6102	0.492	7.307

(1*S*,6*R*)-6-((Cinnamyloxy)carbonyl)cyclohex-3-enecarboxylic acid (9q)

Chiral HPLC (Chiralcel AD-H column), Hexane/*i*-PrOH = 94/6, Flow rate: 0.5 mL/min, UV detection at 254 nm, T = 30°C, retention time: t(minor) = 31.1 min, t(major) = 34.6 min.

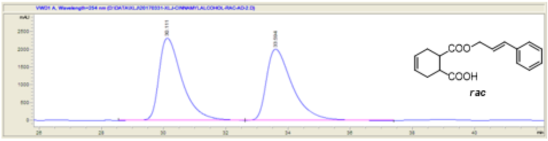

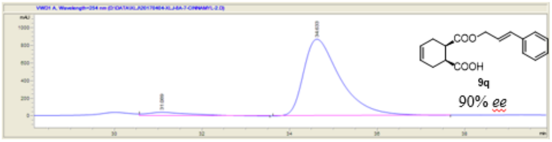

Entry	Time	Peak area	Peak height	Peak width	Symmetric factor	Peak area%
1	30.111	119187.9	2309	0.7802	0.52	50.760
2	33.594	115617.1	2001.5	0.8709	0.511	49.240

Entry	Time	Peak area	Peak height	Peak width	Symmetric factor	Peak area%
1	31.089	2662.8	38.1	0.9612	0.463	5.026
2	34.633	50316.5	865	0.8716	0.53	94.974
